# Health status of transgender people globally: A systematic review of research on disease burden and correlates

**DOI:** 10.1371/journal.pone.0299373

**Published:** 2024-03-11

**Authors:** Ayden I. Scheim, Ashleigh J. Rich, Dougie Zubizarreta, Mannat Malik, Kellan E. Baker, Arjee J. Restar, Leigh Ann van der Merwe, June Wang, Bianca Beebe, Kathleen Ridgeway, Stefan D. Baral, Tonia Poteat, Sari L. Reisner

**Affiliations:** 1 Department of Epidemiology and Biostatistics, Drexel University Dornsife School of Public Health, Philadelphia, Pennsylvania, United States of America; 2 Department of Epidemiology and Biostatistics, Schulich School of Medicine and Dentistry, Western University, London, Ontario, Canada; 3 Department of Social Medicine, University of North Carolina-Chapel Hill, Chapel Hill, North Carolina, United States of America; 4 Department of Epidemiology, Harvard T.H. Chan School of Public Health, Boston, Massachusetts, United States of America; 5 Department of Health Behaviour, Gillings School of Public Health, University of North Carolina-Chapel Hill, Chapel Hill, North Carolina, United States of America; 6 Whitman-Walker Institute, Washington, District of Columbia, United States of America; 7 Department of Epidemiology, School of Public Health, University of Washington, Seattle, Washington, United States of America; 8 Social, Health and Empowerment Feminist Collective of Transgender Women of Africa (S.H.E.), East London, South Africa; 9 Johns Hopkins University, Baltimore, Maryland, United States of America; 10 School of Public Health, Johns Hopkins University, Baltimore, Maryland, United States of America; 11 Department of Epidemiology, School of Public Health, Johns Hopkins University, Baltimore, Maryland, United States of America; 12 Division of Endocrinology, Diabetes and Hypertension, Brigham and Women’s Hospital, Boston, Massachusetts, United States of America; Children’s Mercy Hospital Kansas, UNITED STATES

## Abstract

**Background and objectives:**

Transgender and gender diverse (trans) health research has grown rapidly, highlighting the need to characterize the scientific evidence base. We conducted a systematic review of peer-reviewed research on disease burden and correlates in trans adolescents and adults over a 20-month period to identify knowledge gaps and assess methodological characteristics including measurement of gender identity, community engagement, and study quality.

**Data sources, eligibility criteria, and synthesis methods:**

We searched seven databases using terms related to (a) transgender populations and (b) health or disease. Eligible studies were in English, French, or Spanish and reported original quantitative data on mental health or substance use conditions, infectious diseases, or non-communicable conditions in at least 25 trans individuals aged 15+. Quality assessment was performed in duplicate on a 10% sample of articles and findings were summarized using narrative synthesis.

**Results:**

The 328 included studies were conducted in 45 countries, with most from North America (54%) and limited research from South Asia (3%), Sub-Saharan Africa (3%), and the Middle East and North Africa (2%). Most studies used cross-sectional designs (73%) and convenience sampling (65%). Only 30% of studies reported any form of community engagement. Mental health and substance use disorders were the most studied area (77% of studies) and non-communicable conditions the least (16%). Available data indicated that trans populations experience high disease burden with considerable heterogeneity within and across settings. Of 39 articles assessed for quality, 80% were rated as fair, 18% as poor, and 3% as good quality.

**Conclusions and implications:**

Geographic, gender-specific, and topical gaps remain in trans health, but we found more research from African countries, with transmasculine people, and on non-communicable conditions than previous syntheses. Areas for growth in trans health research include community engagement, non-binary health, chronic and age-related conditions, and health determinants.

**Registration:**

PROSPERO CRD42021234043.

## Introduction

Transgender, non-binary, and other gender diverse people with a gender identity that differs from their assigned sex at birth (collectively referred to as *trans* people herein) represent a substantial and growing proportion of the global population. Current estimates suggest approximately 0.6% of adults and 2.7% of children and adolescents identify as transgender; more expansive definitions of *trans*, inclusive of non-binary and gender non-conforming persons, encompass up to 4.5% of adults and 8.4% of children and adolescents [1–5].

Trans people’s health and well-being is undermined by systemic oppression manifested in intersectional stigmas, criminalization, and barriers to care [6, 7]. Anti-trans stigma has also contributed to historical erasure of trans people in health research, policy, and practice [8], leading to limited availability of data on trans people’s health. In the past decade, however, there has been rapid growth in both trans-focused research and inclusion of measures to identify trans persons in broader studies. A seminal review of quantitative trans health studies from 2008–2014 identified 116 studies from 30 countries, most of which were published in 2013 or 2014 [6]. The review identified knowledge gaps given that most extant research was from the United States (U.S.), with some published literature available in all geographic regions except for Africa, where only one study (in South Africa) was found. In addition, included studies were largely descriptive prevalence studies focused on mental health, sexual and reproductive health (primarily HIV), and/or substance use, and few studies reported data on transmasculine people.

We undertook a systematic review of peer-reviewed research on health burden and correlates in trans populations from 2020 to mid-2021. In this article, we aim to characterize the current state of the science on the health status of trans populations globally by synthesizing geographic, gender-specific, and topical foci and gaps, as well as methodological characteristics relevant for future research and data collection efforts, including measurement of gender identity, community engagement, and study quality. Finally, we provide recommendations to ensure continued growth in the availability, scope, relevance, and real-world impact of data on trans people’s health globally.

## Methods

### Search strategy and selection criteria

We conducted a systematic review of peer-reviewed literature published from 1 January 2020 to 16 August 2021 in English, French, or Spanish. The protocol was registered in PROSPERO (CRD42021234043). The initial protocol included articles published beginning 1 January 2015, but the inclusion criteria were later narrowed due to feasibility given the high volume of publications. We searched Ovid MEDLINE, Embase, PsycInfo, CINAHL Plus, Web of Science, SciELO, and Global Index Medicus using terms related to (a) transgender populations and (b) health or disease. The Ovid MEDLINE search strategy is included as an appendix (S1 Table). We also hand-searched reference lists of systematic reviews on trans health published from 1 January 2020 to 20 July 2022 for articles published within our review’s timeframe.

Studies eligible for inclusion reported original quantitative data on the burden of disease or chronic health conditions in at least 25 trans individuals. For the purposes of this review, we defined *trans* to include individuals who self-identified as transgender, non-binary, and/or as neither a man nor woman, or who were identified as such by study investigators. Intersex persons were only included in this definition if they held a trans identity. We defined disease burden or chronic health condition burden to include prevalence or incidence of any self-reported, laboratory-confirmed, or provider-diagnosed mental health or substance use, infectious, or non-communicable condition or symptomatology. We excluded studies that reported only symptom scale scores (versus prevalence estimates) and studies that were about children, which we defined as studies in which the majority of the sample, or the mean age, was under 15 years. Studies including cisgender persons were eligible if data on trans persons were disaggregated. There were no restrictions on study design. We excluded conference abstracts, non-peer-reviewed literature, qualitative studies, reviews, editorials, and case reports.

References were imported into Endnote version 20 (Clarivate, Philadelphia) and deduplicated before upload to Covidence (Veritas Health Innovation, Melbourne), where all screening and extraction were conducted. Two of five reviewers independently performed title and abstract screening for each record. At this stage, articles were excluded if there was no indication in the abstract that they might report quantitative data on the burden of disease among trans people; if reviewers were unsure about a record, it was retained for full-text screening. A senior author resolved conflicts at this stage. Next, full-text screening was performed in duplicate, with conflicts resolved through discussion at weekly meetings. We documented reasons for exclusion at this stage.

### Data analysis

Data extraction was conducted using a pilot-tested, standardized form in Covidence. We decided *a priori* to extract the first 10% of articles in duplicate before proceeding to single-reviewer extraction; these articles were selected by sorting Covidence records by recency (of any action performed on the record, e.g., importation) and thus are expected to be representative of the larger dataset. Data extraction fields included bibliographic information; study characteristics, including setting and design, sampling method and size, method for ascertaining trans identity/status, theoretical frameworks employed, and reported use of community engagement approaches; sample characteristics, including age range, sex assigned at birth, gender, and sample sizes for each sex/gender group; outcome(s), including ascertainment method and estimates of prevalence or incidence; and exposures statistically significantly associated with outcome(s) at p<0.05. We extracted data on theoretical frameworks and community engagement to evaluate the extent to which research in the field addresses the social determinants of trans health and incorporates a social justice orientation. Four theoretical frameworks commonly employed in trans health research were pre-specified in the extraction form, in addition to a free-text option. Minority stress theory focuses on stressors and resilience factors unique to sexual and gender minority identities [9–11]. The gender affirmation model posits that unmet gender affirmation needs result in identity threat, contributing to behavioral health risks [12, 13]. Intersectionality attends to the impacts of multiple, interlocking systems of oppression, such as transphobia and racism [14–16]. Finally, the syndemics framework examines synergies between co-occurring, socially-produced health conditions in marginalized populations [17].

We performed quality assessment in duplicate for the same 10% subset of articles using the National Institutes of Health–National Heart, Lung, and Blood Institute study quality assessment tool for observational cohort and cross-sectional studies [18]. The tool includes 14 questions assessing risk of bias (ROB) related to sampling and recruitment, exposure and outcome measurement, and confounding. Reviewers were instructed to consider ROB in relation to the study’s primary research question(s), regardless of whether the study’s primary outcomes were extracted for this review. Overall study quality was rated as good, fair, or poor based on reviewers’ assessment of the potential ROB for each study, considering both checklist responses and severity of identified weaknesses. Conflicts in data extraction and quality assessment were resolved through discussion at weekly meetings, and by the first author if consensus could not be reached.

At the time of data extraction, health outcomes were classified using groupings aligned with ICD-11 classifications [19]. We then further grouped outcomes into categories of mental health (including neurodevelopmental conditions) or substance use disorders, infectious diseases, and non-communicable conditions. Correlates of health outcomes were coded into domains after extraction by one of three reviewers who met regularly to develop, pilot-test, and inductively refine the coding framework. The final set of domains included community, social support, and resilience factors; demographics and socioeconomic position; gender affirmation and dysphoria/incongruence; health behaviors, status, and healthcare use; minority stressors (e.g., stigma) and violence; and laws and policies.

We used narrative synthesis methods and descriptive statistics to summarize results [20]. First, we characterized study-level characteristics including study design, sampling methods, trans status ascertainment methods, trans sample size, inclusion of cisgender comparison groups, theoretical frameworks, and geographic distribution of studies. Next, we charted outcomes data across all studies, stratified by outcome category. Narrative synthesis was conducted at both the study and datapoint level. We treated each prevalence or incidence estimate for a particular condition and gender group as a datapoint. Drawing on the descriptions provided by study authors, we classified the gender of study participants as transfeminine (trans women and other trans people assigned male at birth [AMAB]), transmasculine (trans men and other trans people assigned female at birth [AFAB]), non-binary AMAB, non-binary AFAB, or non-binary with unspecified sex assigned at birth. For example, a study reporting on depression and anxiety (separately) among both transfeminine and transmasculine participants would have four datapoints. Finally, correlates of health outcomes were charted by domain and direction of association.

### Ethics statement

Institutional review board approval was not required as this study used only secondary, published data.

## Results

### Study selection

**Fig 1:**
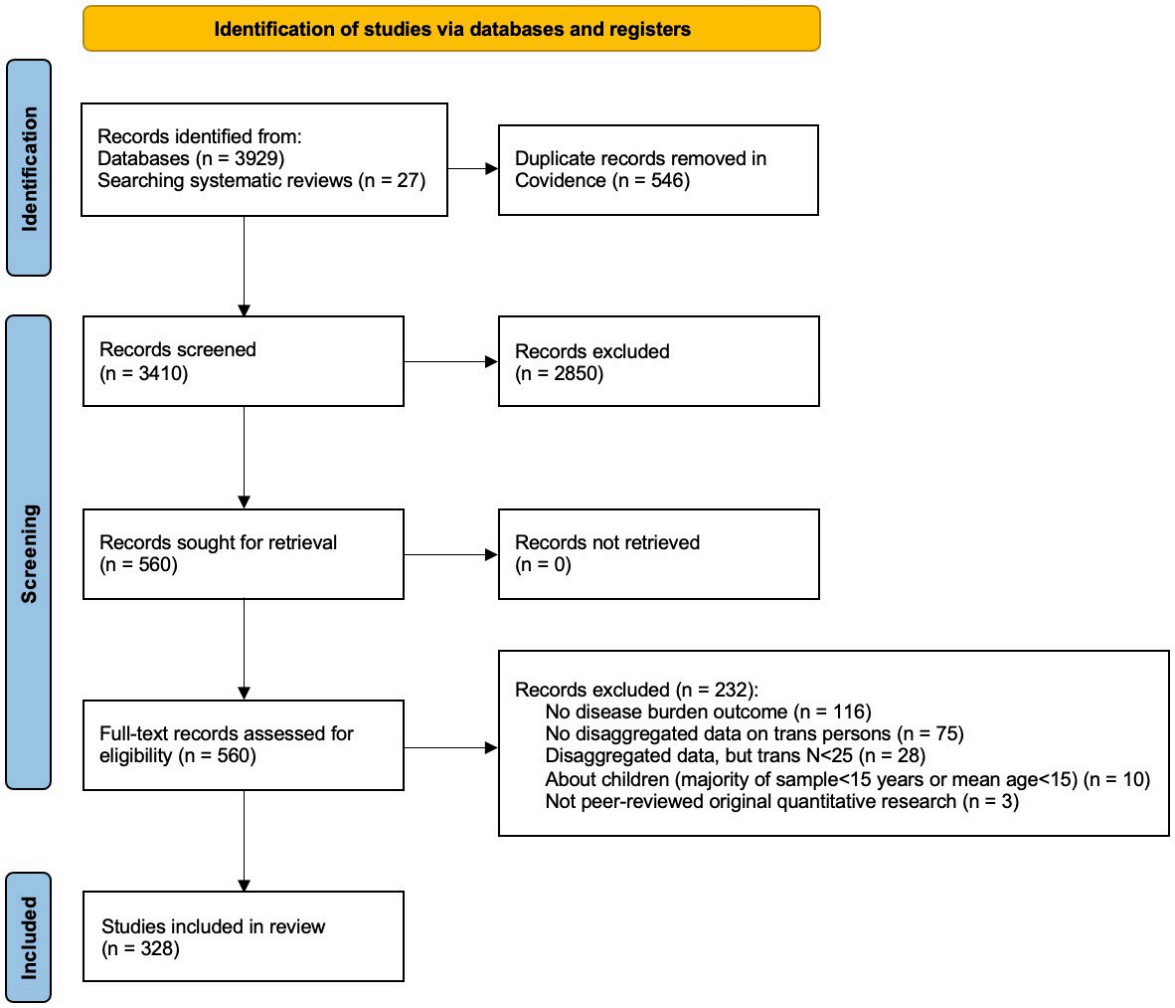
Study selection.

The electronic database search identified 3,929 unique records, of which 561 were assessed for eligibility through full-text screening, and 328 met inclusion criteria (Fig 1). A list of all included studies is available in the supplementary materials (S1 Appendix).

### Characteristics of included studies

As detailed in Table 1, most of the global trans health literature relies on cross-sectional designs (n = 238, 73%), with a scarcity of longitudinal data (14%, n = 43 studies comprised longitudinal cohorts). More than three-fifths of studies used convenience sampling (n = 213, 65%), while 13% (n = 42) sampled through gender clinic records and 10% (n = 32) through electronic medical records. Probability-based sampling or full population censuses were used in 9% (n = 29) of studies, most often population-based surveys. A minority of the literature demonstrated engagement with trans communities, with only approximately one-fifth of studies (n = 68, 21%) reporting any kind of community engagement, most commonly in the context of participant recruitment only (n = 34, 10%) or through establishing study community advisory boards (n = 19, 6%). The median number of trans participants across studies was 234 (IQR: 106–830) and under half (n = 135, 41%) included cisgender comparator groups. Theory plays an important role in the conceptualization and interpretation of health disparities, their underlying mechanisms, and potential interventions [16]; however, there was limited engagement with theory in the global trans health literature. One-quarter of studies included in the review (n = 79, 24%) employed a theoretical framework, most commonly minority stress theory (n = 48). Except for one Spanish-language article [21], all included articles were published in English (not shown).

**Table 1:** Characteristics of included studies (n = 328).

Characteristic	n (%)
**Region**	
East Asia and Pacific	48 (15)
Europe and Central Asia	33 (10)
Latin America and Caribbean	34 (10)
Middle East and North Africa	6 (2)
North America	176 (54)
South Asia	11 (3)
Sub-Saharan Africa	9 (3)
Multi-regional	5 (2)
Unspecified multi-national	5 (2)
**Study design**	
Cross-sectional survey	238 (73)
Cross-sectional survey, repeated	6 (2)
Chart review	32 (10)
Longitudinal cohort, prospective	30 (9)
Longitudinal, retrospective	13 (4)
Case control	1 (<1)
Randomized controlled trial	4 (1)
Non-randomized intervention	1 (<1)
Other/multi design	3 (1)
**Sampling approach**	
Electronic Medical Record, general healthcare	32 (10)
Gender clinic records	42 (13)
Convenience sampling	213 (65)
Population-based survey	24 (7)
Population registry	1 (<1)
Insurance claims	7 (2)
Other probability-based	4 (1)
Multiple methods	5 (2)
**Community engagement**	
None mentioned	260 (79)
For participant recruitment only	34 (10)
Community advisory board or similar	19 (6)
Community-based participatory research	13 (4)
Other/unspecified community engagement	2 (1)
**Measurement of trans status**	
Single-step	68 (21)
Two-step (assigned sex at birth & current gender)	91 (28)
Clinical diagnosis	17 (5)
Receipt or referral for gender-affirming care	38 (12)
Insurance billing codes	20 (6)
Not specified	94 (29)
**Gender spectrum** *	
Transfeminine (assigned male at birth)	96 (29)
Transmasculine (assigned female at birth)	15 (5)
Both	185 (56)
Not specified	32 (10)
**Number of trans participants (median, IQR)**	234 (IQR: 106–830)
**Study includes cisgender comparison group**	135 (41)
**Theoretical framework** *	
None specified	249 (76)
Minority stress	48 (15)
Gender affirmation	7 (2)
Intersectionality	4 (1)
Syndemics	3 (1)
Other	7 (2)
Multiple	10 (3)

*Multiple responses possible; proportions will not sum to 100%.

**Fig 2:**
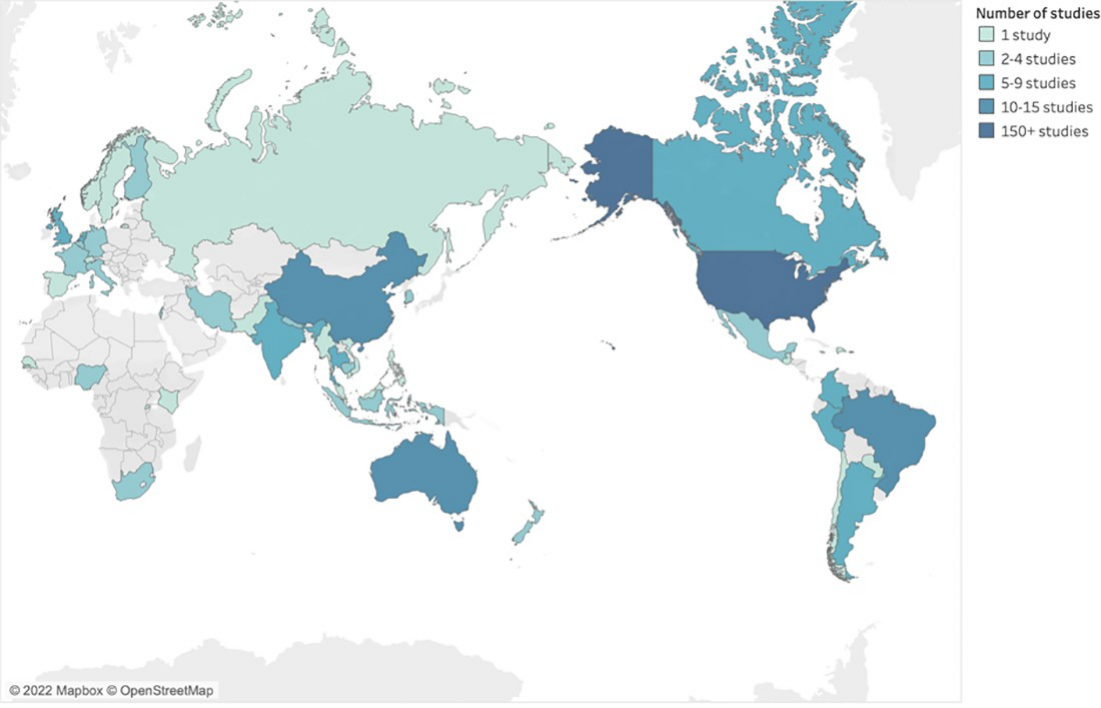
Geographic distribution of studies (n = 328). ©Mapbox, © OpenStreetMap. Map based on Longitude (generated) and Latitude (generated). Color shows details about Heat map group as an attribute. Details are shown for Country. The data is filtered on Heat map group, which excludes Null.

Although studies spanned 45 countries, most were from North America (n = 176, 54%), with limited data from trans populations in South Asia (n = 11, 3%), Sub-Saharan Africa (n = 9, 3%), and the Middle East and North Africa (n = 6, 2%) (Table 1, Fig 2).

Specifically, half of captured studies were conducted in the U.S. (n = 163, 50%), followed by Australia (n = 13, 4%), Brazil (n = 11, 3%), China (n = 10, 3%), Canada (n = 9, 3%), India (n = 8, 2%), the Netherlands (n = 7, 2%), the United Kingdom (n = 7, 2%), and Thailand (n = 6, 2%), with the remaining countries represented in five or fewer studies. The predominance of research from the U.S. prevailed across all disease groups and individual outcomes, limiting knowledge of the health of trans communities in low- and middle-income countries. In addition to contributing the largest number of publications, studies in the U.S. reported the largest trans sample sizes in this review, the largest being 28,980 in an insurance claims-based study of suicidality and mental healthcare utilization [22]. No other countries reported samples exceeding 10,000 participants. The largest non-U.S.-based study used gender clinic records from 8,263 patients in the Netherlands [23].

### Quality assessment

Of 39 articles assessed for quality, 80% (n = 31) were rated as fair, 18% (n = 7) as poor, and 3% (n = 1) as good quality (S2 Table). Most studies rated as good or fair were conducted in the U.S. (19/32) or in other high-income countries (Australia, Canada, Finland, Germany, Norway, United Kingdom; 8/32), while the rest were conducted in Latin America (Brazil, Colombia, Guatemala, Mexico, Paraguay; 5/32). Studies rated as poor were conducted in Brazil, India, Indonesia, Iran, the United Kingdom, the U.S., and an unnamed country (one study each). The most common contributing factors to fair or poor rankings were potential sampling bias, cross-sectional designs precluding casual inference, and concerns about power and sample size. Specifically, only eight out of 39 assessed studies measured the exposure prior to the outcome, only seven reported a participation rate that was at least 50% (most survey-based studies did not report a participation rate), and less than half (n = 15) provided a sample size justification, power description, and/or variance estimates. A frequent source of potential bias was recruitment of participants from specialized clinics, which overlooks trans people who cannot access these clinics for financial or other reasons, or who are not seeking medical gender affirmation [24].

Reviewers provided additional justification for rating studies as “poor,” which included exclusions of substantial numbers of participants from the sample without clear explanation, very low response rates or high loss to follow-up, and lack of information about study population or recruitment processes. Of note given concerns about data quality in relation to the small size of the trans population, seven studies did not specify how gender identity was measured for purposes of defining the study population.

### Gender identity and trans status measurement

Despite the known impact of measurement approaches on estimates of trans population size and trans health disparities [25], almost one-third of studies did not specify how gender identity was ascertained (n = 94, 29%). Of studies that reported trans status ascertainment method (n = 234, 71%), most prevalent was the “two-step” method (n = 91, capturing both current gender and sex assigned at birth via two separate items [26]), followed by “single-step” measures that assess gender identity only (and include trans-specific options) or ask directly about transgender self-identification (n = 68). In addition, many studies ascertained trans status via receipt of or referral for gender-affirming care (n = 38) or insurance billing codes (n = 20). Most studies included both transmasculine and transfeminine populations (n = 185, 56%), with single gender samples most frequently reported for trans women or transfeminine people (n = 96, 29%). Approximately 10% of studies (n = 32) did not specify the gender identity of trans participants, limiting interpretation of results and their applicability to intervention development.

**Fig 3:**
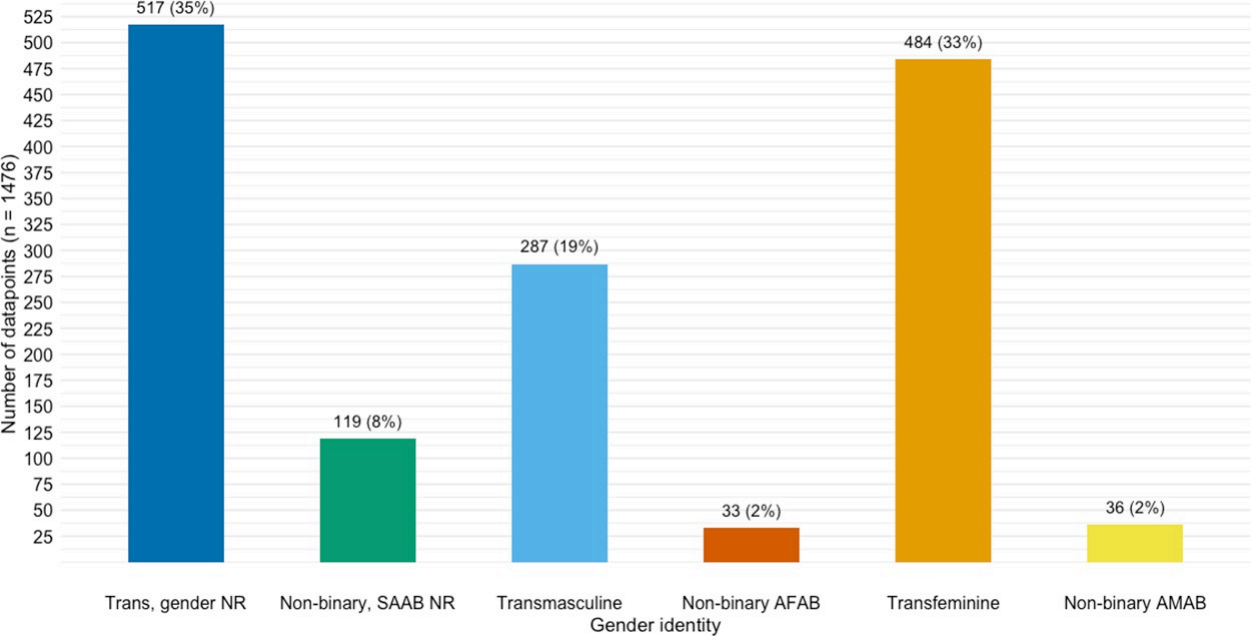
Gender distribution of datapoints (n = 1476). Abbreviations: SAAB = sex assigned at birth, AMAB = assigned male at birth, AFAB = assigned female at birth, NR = not reported. Note: Transmasculine includes transmasculine people and trans men; transfeminine includes transfeminine people and trans women.

Examining the distribution of gender-specific datapoints (Fig 3), most were not disaggregated by current gender identity and/or sex assigned at birth (n = 517, 35%) or were only for transfeminine people (n = 484, 33%). There were fewer datapoints for transmasculine people (n = 287, 19%) and non-binary people (n = 188, 12%). Of datapoints for non-binary people, most (n = 119, 63%) were not disaggregated by sex assigned at birth.

### Health burden

**Fig 4:**
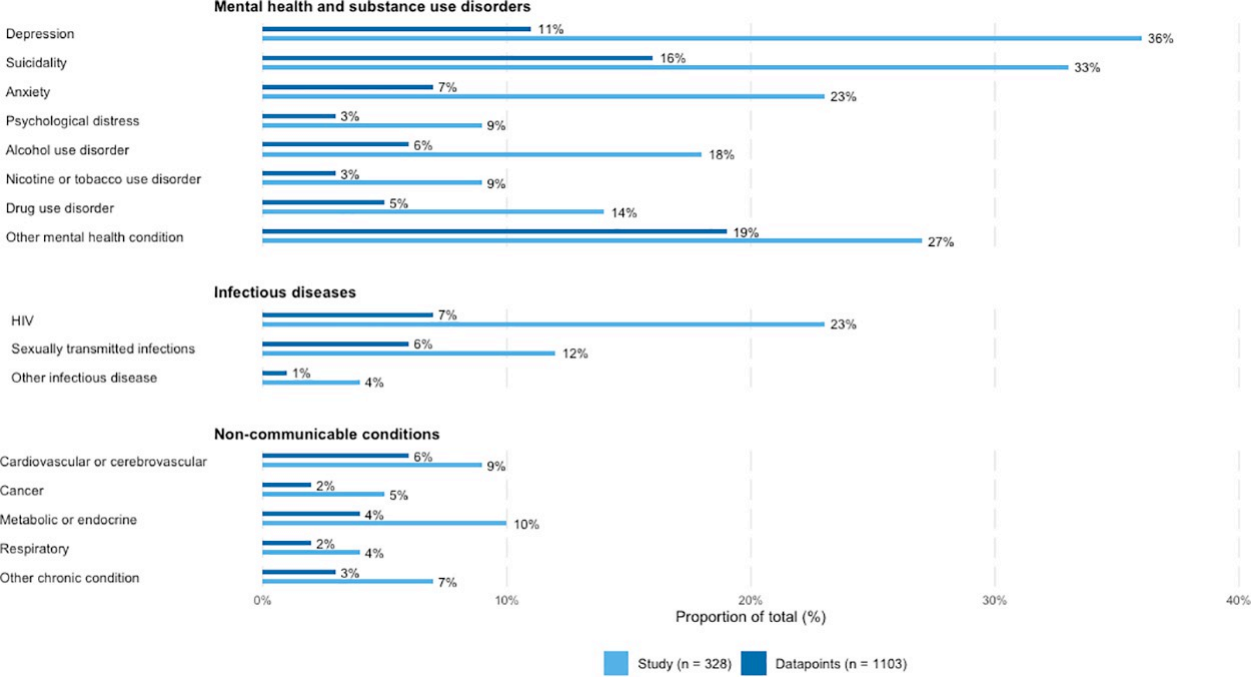
Number of studies and datapoints for mental health and substance use disorders, infectious disease outcomes, and non-communicable conditions. Note: Percentages will add to 100% for datapoints, but will not add to 100% for studies since studies could report on multiple outcomes.

We identified 1,103 datapoints on health burden from our sample of 328 studies (S3 Table). Fig 4 presents these datapoints grouped into three broad outcome categories: (1) mental health and substance use disorders, (2) infectious diseases, and (3) non-communicable conditions.

#### Mental health and substance use disorders

Consistent with previous reviews [6, 27], mental health and substance use disorders remain the most studied area of trans health (77% of studies, n = 252; 70% of datapoints, n = 773). Depression, suicidality, and other mental health conditions (e.g., self-harm; post-traumatic stress disorder [PTSD]; eating disorders) were the most frequently reported (16%, n = 123; 23%, n = 177; and 27%, n = 207 of datapoints respectively). The least reported mental health and substance use outcomes were drug use disorders (8% of datapoints, n = 58), nicotine use disorders (4% of datapoints, n = 33), and psychological distress (4% of datapoints, n = 28). There was substantial heterogeneity in the measurement and operationalization of mental health and substance use outcomes across studies. For example, studies assessing depression used various ascertainment methods (e.g., scales such as the Center for Epidemiologic Studies Depression Scale [CES-D], self-report, diagnosis) and timeframes of ascertainment (e.g., lifetime, past year, past two weeks). Nevertheless, there was consistent evidence of poor mental health and substance use burden for trans people across studies. Specifically, prevalence estimates for depression were as high as 95.5% (CES-D score≥16) among 44 non-binary people aged 18–35 in a community-based cross-sectional study in the U.S. [28], and were as low as 2.1% (assessed using the Patient Health Questionnaire-4) among 47 trans women seeking gender-affirming surgery in a retrospective longitudinal cohort study conducted in Germany [29]. We found growing attention to PTSD (3% versus 0.6% of datapoints) and eating disorders (6% versus 0.6% datapoints) in the current trans health literature compared to Reisner et al.’s 2016 review [6].

#### Infectious disease

Infectious disease was the second most studied area in this review (29% of studies, n = 94; 14% of datapoints, n = 157). In line with HIV as a priority area for research funding and attention in trans health, HIV was the most frequently reported infectious disease (50% of datapoints, n = 78), followed by other sexually transmitted infections (STIs; 39%, n = 61), leaving relatively little evidence on other infectious diseases (e.g., COVID-19; 3%, n = 5). Data on HIV and other STIs among people assigned female at birth were limited (18 datapoints versus 110 among people assigned male at birth). Among those assigned male at birth, HIV prevalence was as high as 71.1% among 149 trans women in a cross-sectional study using respondent-driven sampling in Nigeria [30] and as low as 0% among 49 transfeminine individuals seeking gender-affirming medical care in a retrospective chart review study conducted in the U.S. [31]. Among those assigned female at birth, HIV prevalence was as high as 3.5% among 176 AFAB men who have sex with men in a cross-sectional online study conducted in Europe [32], and as low as 0% among 25 trans men in a cross-sectional online study conducted in the U.S. [33] and among 31 transmasculine individuals seeking gender-affirming medical care in a retrospective chart review study conducted in the U.S. [31].

#### Non-communicable conditions

Non-communicable conditions were the least studied area of trans health (16% of studies, n = 52; 16% of datapoints, n = 173), pointing to an important area for future research, particularly considering evidence linking multi-level stress to inflammation-related chronic disease [27, 34]. Most non-communicable conditions data relate to cardiovascular or cerebrovascular (38% of datapoints, n = 65), metabolic or endocrine (23%, n = 40), or other non-communicable conditions (e.g., kidney disease or arthritis; 19% overall, n = 33). Trans people experience a high burden of non-communicable conditions: for example, hypertension prevalence was as high as 73% in a cross-sectional study of 221 trans women living with HIV in the U.S. [35], and diabetes prevalence was as high as 24.7% among 88 trans people aged 45 and older in a population-based survey in the U.S. [36]. There was a dearth of research on age-related non-communicable conditions, including dementia (n = 0 datapoints), arthritis (n = 6 datapoints), cancer (n = 18 datapoints), and hypertension (n = 23 datapoints).

### Risk and protective factors

Identification of risk and protective factors is essential to inform interventions to reduce morbidity for trans populations. Less than half of included studies examined correlates of health outcomes extracted for this review among trans participants (41%, n = 134), with a total of 636 risk or protective factors, including 268 unique factors, reported across studies and outcomes (S4 Table). Across studies, most correlates identified were associated with increased burden (i.e., risk factors; n = 566 datapoints, 89%) versus reduced burden (i.e., protective factors; n = 70 datapoints, 11%) of disease.

The most commonly reported outcomes for which risk or protective factors were identified were suicidality (170 correlates, 27%), depression (128 correlates, 20%), other mental health conditions (77 correlates, 12%), anxiety (63 correlates, 10%), psychological distress (44 correlates, 7%), and HIV (37 correlates, 6%), while the least common were respiratory conditions (2 correlates, <1%), cancer (2 correlate, <1%), and other infectious diseases (1 correlate, <1%). The most common categories into which correlates were classified were demographics and socioeconomic position (n = 227, 36%), followed by minority stressors and violence (n = 139, 22%), and health behaviors, health status, and healthcare use (n = 124, 20%). The least common category was for the policies domain (n = 7, 1%). The most common correlates of health outcomes among trans people were older age (n = 27, 4%), discrimination (n = 15, 2%), younger age (n = 14, 2%), autism (n = 13, 2%), non-binary identity (n = 12, 2%), and housing issues (n = 12, 2%), with a quarter of correlates reported only once across studies and outcomes (n = 150, 24%). We caution that these frequencies are impacted by studies exploring multiple outcomes (e.g., data on autism come from two studies that provided 13 datapoints [37, 38]). Importantly, across studies and outcomes gender affirmation was consistently protective and its absence consistently a risk for disease burden, including for suicidality, depression, anxiety, and cardiovascular and cerebrovascular conditions, highlighting the critical role of gender affirmation in trans health promotion. The most frequently reported protective factors in this area were access to gender-affirming care (5 datapoints) and legal name change or gender-concordant identification documents (3 datapoints each). The most frequently reported risk factors in the gender affirmation and dysphoria/incongruence domain were lack of gender-affirming care access (15 datapoints), negative media messaging about trans people (4 datapoints), and body dysphoria (4 datapoints).

## Discussion

There has been considerable growth in trans health research output since the 2016 global synthesis that reviewed 116 eligible studies over a seven-year period from 2008–2014 [6]. In the current review, we identified 328 studies providing data on health burden among trans people published over a 20-month period. This increase in volume is due not only to growth in trans-specific research, but also efforts to include and identify trans people in broader population studies; four out of every ten included studies had both cisgender and transgender participants. However, there remain notable gaps in gender-specific and global research on trans populations and in specific domains of health.

The geographic diversity of trans health research has grown. For instance, while quantitative data on trans health in African countries were largely unavailable in 2014, studies with trans populations in Kenya, Malawi, Nigeria, Rwanda, Senegal, and South Africa were included in this review [30, 39–46]. Nevertheless, research remains concentrated in high-income countries in the Global North, with many countries still having little or no published data on trans populations. It is also promising to see disaggregation of trans health data by sex and gender, as well as increasing availability of data specific to transmasculine and non-binary people. However, many studies did not disaggregate health outcomes among trans people by gender, overlooking gender identity differences that may be relevant for health and intervention development [47]. Of those that specified gender identity and/or sex assigned at birth, there were fewer datapoints for transmasculine individuals or people assigned female at birth. This demonstrates the importance of studies either reporting participants’ gender identity and sex assigned at birth and presenting health outcomes stratified by gender identity or providing a rationale for not providing stratified data. To guide this decision, researchers should consider whether the question at hand calls for gender-inclusive or gender-specific analysis [48]. The greater number of transfeminine-only studies is likely due to the continued predominance of HIV in global trans health research. Ongoing investment is needed in research on the holistic health needs of trans people globally, including non-binary and transmasculine populations [49].

Research included in this synthesis demonstrates a high burden of mental health, infectious diseases, and non-communicable conditions in trans populations. However, non-communicable chronic conditions, including age-related conditions, remain under-studied. With respect to mental health and infectious disease, disparities in outcomes such as depression, anxiety, and HIV are now well-documented across a range of settings [50, 51]. Identifying individual, network, and structural determinants of health is critical to the development and implementation of evidence-based interventions to reduce morbidity for trans populations. Notably, less than half of studies included in this review examined correlates of health outcomes. There is a need to continue identifying trans vulnerabilities and resiliencies for interventions, including intersectional stigmas and social, legal, and medical gender affirmation [12, 16]. Few studies examined the relationship between legal and policy factors and trans health outcomes, which is particularly important in the context of global attacks on trans rights. Similarly, just seven studies took an explicitly intersectional approach. It is vital to consider trans status alongside other social identities or positions to understand and address the multiple interlocking systems of power, privilege, and oppression (e.g., cissexism, ethno-racism, classism) that shape patterns of trans health [16]. The myriad ways trans people resist intersecting oppressions and promote community well-being is an important yet neglected area of strength-based research [52, 53].

Of the subset of 39 articles assessed for risk of bias, only one was rated as good quality. Studies that do not meet traditional epidemiologic standards of rigor may nevertheless contribute to trans population health by raising awareness and generating hypotheses. However, this finding indicates the importance of greater investment by public and private research institutions and funders in advancing methods for trans health research globally. This includes enhanced training for researchers in academic, clinical, and community environments in methodologies appropriate for assessing the characteristics of small and marginalized populations, as well as an emphasis on community-engaged research practices that prioritize trans community concerns and center equity in sharing the benefits and burdens of research [54].

More standardized data collection practices, in which gender identity and trans experience are consistently defined, measured, and reported during study design, recruitment, analysis, and publication are also essential for continuing to build the body of evidence related to trans health [55]. Historically, many researchers have sought to measure trans identity as part of the measurement of sex or gender. Many trans people identify simply as men, women, or another gender, however, which renders them invisible in questions such as “What is your gender?” with the response options “man,” “woman,” and “transgender” [56, 57]. To capture the broader category of trans experience, in the 1990s, community-based HIV researchers in the U.S. developed an approach that has come to be known as the “two-step” method [26]. A common form of the two-step is an item that asks about sex assigned at birth on the person’s original birth certificate, followed by a second item that asks about current gender. The second item allows for categorization of respondents by gender, while the cross-tabulation between the two items allows for categorization of respondents as transgender or cisgender. In survey research settings, two-step designs have shown good reliability and validity and very low item non-response rates [26] and are recommended in 2022 measurement recommendations from the U.S. National Academies [55]. Promisingly, this approach was used by almost one-third of included studies that did report on how they ascertained gender identity. Other studies identified trans participants based on receipt of gender-affirming care or using insurance claims or medical records, which can be queried for trans-related codes [58]. These approaches, however, only identify trans people who have sought health care (typically related to medical gender affirmation) and are unlikely to be representative of the trans population at large. Regarding the measurement of health conditions, clinical samples may be biased in either direction: more encounters with the health care system provides more opportunities for conditions to be identified and diagnosed, which may overestimate their burden in the trans population at large. At the same time, trans people engaged in care may be healthier than the general trans population, leading to an underestimation of poorer health. Administrative data also tend to have poor records of important variables such as social determinants of health, race, and ethnicity, which hinders assessment of the intersectional impacts of these factors on the health of trans populations [59, 60]. While these data can still contribute important knowledge and assist with planning of clinical interventions, they fail to provide a picture of trans population health that is fully adequate for the design and evaluation of community interventions. Whenever possible, self-reported demographic data is the preferred method for identifying and conducting research with trans people and populations [26, 61]. The routine inclusion of gender identity and trans experience variables in population surveys and other data collection systems administered by governments and private entities will assist in broadening the number of high-quality data sources available for trans health research.

Alongside scientific rigor, meaningful engagement of trans people is critical to ensure the relevance and success of research and to translate research into effective interventions [62, 63]. A concerning eight out of every 10 studies reviewed did not report any trans community engagement. Community-engaged research (CER) exists along a spectrum, from involving community members in study recruitment (10% of studies) to engaging community advisory boards or similar bodies to inform the research (6% of studies) to community-based participatory research (CBPR; 4% of studies). CBPR refers to research in which non-academic researchers who are part of the community under study participate in all stages of the research process [62, 64]. In community-led or community control models of CBPR, community members are key decision makers at all phases of the research, including conceptualization and design [65]. Drawing on these models of CER, trans health research priorities should be set in partnership with, and ultimately benefit, trans communities [66, 67]. Researchers newly entering the space of trans health should be reflective about their roles and take steps to assure they are addressing priorities in the field; recent scholarship on avoiding “health equity tourism” may be helpful for researchers embarking on this work [68].

### Limitations

It is important to consider the findings of this review in light of its limited scope. We initially proposed to review articles published from 1 January 2015 onwards, however, this was not feasible due to the number of potentially eligible articles identified (e.g., over 1000 articles eligible for full-text review from MEDLINE alone). As such, this synthesis provides a snapshot of what is being studied (and how) in trans health, rather than a longitudinal analysis or in-depth synthesis of the prevalence of specific conditions in trans populations. The distribution of studies across countries is likely the result most sensitive to our choice of timeframe, as countries may generate less than one trans health study per year; we therefore focus on regional patterns when interpreting results. In addition, the field would benefit from more narrative and quantitative syntheses of disease burden over longer timeframes, such as recently published reviews on chronic disease [27] and HIV [50]. Only studies published in English, French, or Spanish were eligible for inclusion and almost all included studies were in English. However, it is unclear whether inclusion of additional language would have meaningfully impacted results. We note that a review of trans mental health research in China identified just two Chinese-language articles from 2015 to 2021 [69] and a global review of transmasculine health research from 1999 to 2019 found no peer-reviewed articles in Hindi [49].

We also excluded qualitative research studies and non-peer-reviewed studies, which are rich sources of data on trans health and needs in diverse local contexts. Our synthesis methods also have limitations. We sometimes report data at the datapoint-level rather than the study-level. Because studies with more datapoints contribute more data, doing so may inflate some estimates. Our analysis of correlates of health outcomes focused only on those which were statistically significantly associated and thus gaps identified may reflect both a lack of investigation (e.g., of policy exposures) and null findings.

### Conclusion

The quantity of available data on trans people’s health globally in 2020–2021 is remarkable when compared to the state of the science just five years ago. The field now has an opportunity to address specific data needs and opportunities, including about specific trans populations (e.g., non-binary people outside the US), topic areas (e.g., non-communicable chronic diseases), geographic regions (e.g., in Asia and Africa), protective factors (e.g., mutual aid, resistance strategies), interventions (e.g., RCTs, natural experiments), methods and measures (e.g., difference-in-differences for policy analyses, harmonizing gender identity questions), and theoretical frameworks (e.g., in addition to minority stress). The growing evidence base on health inequities faced by trans people requires a comprehensive and holistic public health response, including but not limited to access to affirming primary and preventive care as well as medical gender affirmation (e.g., hormones and surgery). Ultimately, addressing the burden of mental health, infectious, and chronic conditions in trans populations requires tackling structural determinants of trans health outside the healthcare system, such as poverty, criminalization, legal gender recognition, and intersecting forms of stigma and discrimination [16]. Dedicated funding, including for trans-led initiatives, is needed to translate research into effective public health programs and policies for trans people globally.

## Supporting information

S1 Table MEDLINE search strategy. (DOCX)

S2 Table Quality appraisal. (DOCX)

S3 Table Studies and study characteristics included in systematic review. (DOCX)

S4 Table Risk and protective factors. (DOCX)

S1 Appendix All articles included in review. (DOCX)

S1 Checklist PRISMA 2009 checklist. (DOC)
