# Supplementary material for: Health status of transgender people globally: A systematic review of research on disease burden and correlates
Source: PLoS One. 2024 Mar 11;19(3):e0299373. doi: 10.1371/journal.pone.0299373 (PMC10927095; doi:10.1371/journal.pone.0299373)
Supplement: S1 Table — (DOCX) [file pone.0299373.s001.docx]

**Supplementary Table S1. MEDLINE Search Strategy**

| **Concept** | **Search string** |
| --- | --- |
| 1: Health and disease | exp Health/ or "Health Services for Transgender Persons"/ or exp Public Health/ or exp Health Surveys/ or exp Health Status/ or Health Risk Behaviors/ or Health Equity/ or exp Risk Factors/ or exp Disease/ or exp Behavioral Symptoms/ or exp Mental Disorders/ or exp Psychological Trauma/ or exp HIV Infections/ or exp HIV/  or (chronic* adj2 (hepat* or liver* or kidney* or obes* or mental* or anxiet* or depress* or substance* or drug* or alcohol* or respiratory or asthma or cardio* or heart or pain or diabet* or arthrit*)).ab,kw,ti.  or ((chronic* or infect* or longterm or long-term or "long term") adj2 (condition* or ill* or disease*)).ab,kw,ti.  or (((respiratory or cardio* or heart or liver or kidney or renal or hepato*) adj2 (disease* or condition* or illness*)) or obes* or (cancer* or tumor* or malignan* or carcinoma*) or diabet* or "human immunodeficiency virus" or "acquired immunodeficiency syndrome").ab,kw,ti.  or (((mental or "mental health" or psychological* or psychiatric*) adj1 (disorder* or condition*)) or depress* or anxiet* or suicid* or selfharm or self-harm or "self harm" or selfinjur* or self-injur* or (self adj2 injur*) or ((substance* or drug* or alcohol*) adj2 (disorder* or dependence* or abuse* or use*))).ab,kw,ti.  or (well-being or "well being").ab,kw,ti. |
| 2: Transgender identity | exp Transgender Persons/ or Transsexualism/ or Transvestism/ or Gender Identity/ or exp Sex Reassignment Procedures/  or ((Transgender* or Trans?exual* or Transvest* or transwom#n or "trans wom#n" or transm#n or "trans m#n") or (nonbinary or "non-binary" or "non binary") or (travesti* or hijra* or kat?oey* or mahu* or waria* or crossdress* or "cross-dress*" or "cross dress*")).ab,kw,ti.  or (Gender adj1 (identity* or dysphor* or minorit* or divers* or var* or nonconforming or non-conforming or "non conforming")).ab,kw,ti. |
