# Supplementary material for: Health status of transgender people globally: A systematic review of research on disease burden and correlates
Source: PLoS One. 2024 Mar 11;19(3):e0299373. doi: 10.1371/journal.pone.0299373 (PMC10927095; doi:10.1371/journal.pone.0299373)
Supplement: S2 Table — (DOCX) [file pone.0299373.s002.docx]

**Supplementary Table S2. Quality Appraisal**

| **Citation** | **Research Question** | **Study Population** | **Participation** | **Recruitment & Inclusion** | **Sample size** | **Exposure Temporality** | **Timeframe** | **Exposure Levels** | **Exposures Valid** | **Exposure >1** | **Outcomes Valid** | **Assessors Masked** | **Loss to Follow-Up** | **Confounding** | **Overall rating** |
| --- | --- | --- | --- | --- | --- | --- | --- | --- | --- | --- | --- | --- | --- | --- | --- |
| Abramovich 2020 | Y | N | NA | N | N | NA | NA | NA | CD | NA | Y | NA | NA | CD | Fair |
| Achille 2020 | Y | N | CD | CD | N | Y | Y | Y | Y | Y | Y | NA | N | N | Poor |
| Aguilar 2020 | Y | Y | NR | Y | N | N | NA | NA | Y | N | Y | NA | NA | NA | Fair |
| Aldridge 2020 | Y | Y | N | Y | N | Y | Y | NA | Y | N | Y | NA | N | Y | Fair |
| Allen 2021 | Y | Y | NR | CD | Y | Y | Y | NA | Y | NA | N | CD | NR | CD | Fair |
| Alvarado 2020 | Y | Y | NR | CD | N | N | N | NA | Y | NA | Y | NA | NA | Y | Fair |
| Anderssen 2020 | Y | CD | N | N | N | N | N | NA | N | NA | Y | NA | NA | Y | Fair |
| Andrew 2020 | Y | N | NR | NR | N | N | N | Y | Y | NA | Y | NA | NA | N | Poor |
| AndrewYockey 2020 | Y | Y | NR | Y | Y | N | N | N | NR | NA | N | NA | NA | Y | Fair |
| Angoff 2021 | Y | Y | NR | NR | Y | N | NA | NA | NA | N | Y | NA | NA | Y | Fair |
| Antebi-Gruszka 2020 | Y | Y | CD | Y | N | N | N | N | Y | NA | Y | NA | NA | Y | Fair |
| Becerra 2021 | Y | Y | CD | Y | Y | N | N | N | Y | N | Y | NA | NA | Y | Fair |
| Biedermann 2021 | Y | Y | NR | Y | Y | N | N | Y | Y | N | Y | NR | NA | Y | Fair |
| Blosnich 2021 | Y | Y | NA | Y | Y | N | N | NA | Y | N | Y | CD | NA | Y | Fair |
| Ferlatte 2020 | Y | Y | CD | Y | N | N | N | Y | Y | N | Y | NA | NA | Y | Fair |
| Flentje 2020 | Y | N | CD | Y | N | NA | NA | NA | NA | NA | Y | NA | NA | NA | Fair |
| Fontanari 2020 | Y | Y | NR | Y | Y | N | N | Y | Y | N | Y | N | NA | N | Fair |
| Hein 2021 | Y | Y | Y | Y | N | N | N | NA | N | NA | Y | NA | NA | Y | Fair |
| Hibbert 2020 | Y | Y | CD | Y | N | N | N | Y | Y | N | Y | NA | NA | Y | Fair |
| Indrawati 2020 | Y | N | CD | CD | Y | N | N | CD | N | N | Y | CD | NA | CD | Poor |
| Jacoby 2021 | Y | N | NA | Y | NA | NA | NA | NA | NA | NA | Y | NA | NA | NA | Fair |
| Lane 2020 | Y | Y | Y | Y | Y | NA | NA | NA | NA | N | Y | N | NA | NA | Fair |
| Linsenmeyer 2021 | Y | Y | NR | Y | N | N | N | NA | Y | N | Y | NA | NA | N | Fair |
| Martin 2021 | Y | Y | NR | Y | N | N | N | NA | Y | N | Y | NA | NA | Y | Fair |
| Miller 2020 | Y | Y | CD | CD | Y | N | N | Y | Y | N | Y | N | NA | Y | Fair |
| Mohajer 2020 | Y | N | Y | NR | N | N | N | Y | Y | NA | Y | N | NA | N | Poor |
| Parr 2020 | Y | Y | NR | Y | Y | N | N | NA | Y | N | Y | NA | NA | Y | Good |
| Pines 2021 | Y | Y | CD | N | N | NA | NA | NA | NA | NA | Y | NA | NA | N | Fair |
| Poteat 2020 | Y | Y | NR | Y | N | N | N | NA | Y | N | Y | NR | NA | N | Fair |
| Prescott 2020 | Y | Y | NR | Y | Y | N | N | Y | Y | N | Y | NA | NA | N | Fair |
| Real 2021 | Y | N | NR | N | Y | N | N | NA | Y | N | Y | NR | NA | Y | Poor |
| Rodriguez-Hart 2021 | Y | Y | NA | Y | NR | N | NA | NA | Y | N | Y | NA | NA | N | Fair |
| Sartaj 2020 | Y | N | NR | CD | N | N | N | Y | Y | N | Y | N | NA | N | Poor |
| Sergi 2021 | Y | Y | CD | Y | N | N | N | N | N | NA | Y | NA | NA | N | Fair |
| Singer 2020 | Y | Y | NR | Y | Y | N | N | NA | Y | NA | Y | NA | NA | CD | Fair |
| Steele 2020 | Y | Y | N | Y | N | N | N | NA | NA | NA | Y | NA | NA | NA | Poor |
| Stogner 2021 | Y | Y | CD | Y | N | N | N | Y | Y | N | Y | NA | NA | Y | Fair |
| Tebbe 2021 | Y | Y | CD | Y | N | N | N | Y | Y | N | Y | NA | NA | N | Fair |
| Turban 2021 | Y | Y | NR | Y | N | N | N | NA | Y | N | Y | NA | NA | Y | Fair |

Y = Yes; N= No; NA= Not applicable; NR = Not reported; CD = Cannot determine

**Research Question:** Was the research question or objective in this paper clearly stated?

**Study Population:** Was the study population clearly specified and defined?

**Participation:** Was the participation rate of eligible persons at least 50%?

**Recruitment & Inclusion:** Were all the subjects selected or recruited from the same or similar populations (including the same time period)? Were inclusion and exclusion criteria for being in the study prespecified and applied uniformly to all participants?

**Sample Size:** Was a sample size justification, power description, or variance and effect estimates provided?

**Exposure Temporality:** For the analyses in this paper, were the exposure(s) of interest measured prior to the outcome(s) being measured?

**Timeframe:** Was the timeframe sufficient so that one could reasonably expect to see an association between exposure and outcome if it existed?

**Exposure Levels:** For exposures that can vary in amount or level, did the study examine different levels of the exposure as related to the outcome (e.g., categories of exposure, or exposure measured as continuous variable)?

**Exposure Valid:** Were the exposure measures (independent variables) clearly defined, valid, reliable, and implemented consistently across all study participants?

**Exposure >1:** Was the exposure(s) assessed more than once over time?

**Outcomes Valid:** Were the outcome measures (dependent variables) clearly defined, valid, reliable, and implemented consistently across all study participants?

**Assessors Masked:** Were the outcome assessors blinded to the exposure status of participants?

**Loss to Follow-Up:** Was loss to follow-up after baseline 20% or less?

**Confounding:** Were key potential confounding variables measured and adjusted statistically for their impact on the relationship between exposure(s) and outcome(s)?
