# Supplementary material for: Health status of transgender people globally: A systematic review of research on disease burden and correlates
Source: PLoS One. 2024 Mar 11;19(3):e0299373. doi: 10.1371/journal.pone.0299373 (PMC10927095; doi:10.1371/journal.pone.0299373)
Supplement: S3 Table — (DOCX) [file pone.0299373.s003.docx]

**Supplementary Table S3. Studies and study characteristics included in systematic review (n=329 studies, 1106 datapoints)**

| Study ID | Country | Trans sample size | Dataset | Age range | Subgroup | Specific outcome; ascertainment method | Outcome measure | Total prevalence or incidence | Trans feminine incidence or prevalence | Trans masculine incidence or prevalence | Trans women incidence or prevalence | Trans men incidence or prevalence | Non-binary AMAB incidence or prevalence | Non-binary AFAB incidence or prevalence | Non-binary incidence or prevalence |
| --- | --- | --- | --- | --- | --- | --- | --- | --- | --- | --- | --- | --- | --- | --- | --- |
| Chronic conditions | | | | | | | | | | | | | | | |
| Cancer | | | | | | | | | | | | | | | |
| Abramovich 2020 | Canada | 2085 | No study name provided | NA | NA | Cancer; diagnosis | Prevalence | 1.30% | NA | NA | NA | NA | NA | NA | NA |
| Boehmer 2020 | United States | 4097 | BRFSS (Behavioral Risk Factor Surveillance System) | 18+ | People with a cancer diagnosis, other than skin cancer | Cancer ever, aside from skin cancer; self-report | Prevalence | NA | NA | NA | 6.80% | 9.70% | NA | NA | 7.60% |
| Bretherton 2021 | Australia | 928 | No study name provided | 18+ | NA | Cancer ever; self-report | Prevalence | 2.00% | NA | NA | NA | NA | NA | NA | NA |
| deBlok 2021 | Netherlands | 2616 | No study name provided | 18+ | NA | Breast cancer after the start of hormone treatment; diagnosis | Prevalence | NA | NA | NA | 10.00% | NA | NA | NA | NA |
| deNie 2020 | Netherlands | 2281 | Nationwide Network and Registry of Histopathology and Cytopathology in the Netherlands (PALGA) | 18+ | TW receiving GAHT | Diagnosis with prostate cancer after median 17 years of hormone treatment; diagnosis | Prevalence | NA | NA | NA | 0.30% | NA | NA | NA | NA |
| deNie 2021 | Netherlands | 3026 | Dutch National Pathology Database (PALGA) | 18+ | TW utilizing GAHT | Cases of testicular cancer within study cohort; diagnosis | Prevalence | NA | NA | NA | 0.10% | NA | NA | NA | NA |
| Gilbert 2020 | United States | 137 | No study name provided | 18+ | NA | Cancer diagnosis ever; self-report | Prevalence | 5.10% | NA | NA | NA | NA | NA | NA | NA |
| Hawkins 2021 | United States | 81 | No study name provided | 18+ | Transmasculine patients on testosterone presenting for hysterectomy | Endometrial hyperplasia or malignancy; diagnosis | Prevalence | NA | NA | 0.00% | NA | NA | NA | NA | NA |
| James 2020 | United States | 82 | Rochester Epidemiology Project | NA | Patients seeking gender-related healthcare | Cancer; diagnosis | Prevalence | NA | 6.10% | 0.00% | NA | NA | NA | NA | NA |
| Lett 2020 | United States | 3151 | BRFSS (Behavioral Risk Factor Surveillance System) | NA | NA | Cancer; self-report of physician diagnosis | Prevalence | Black: 7.2%; White: 13.5% | NA | NA | NA | NA | NA | NA | NA |
| Mohr 2021 | Switzerland | 98 | No study name provided | NA | NA | Anal squameous cell carcinoma; diagnosis | Prevalence | 1.00% | NA | NA | 1.90% | NA | NA | NA | NA |
| Pharr 2021 | United States | 2827 | BRFSS (Behavioral Risk Factor Surveillance System) | 18+ | NA | Skin cancer; self-report | Prevalence | 7.00% | NA | NA | NA | NA | NA | NA | NA |
| Pharr 2021 | United States | 2827 | BRFSS (Behavioral Risk Factor Surveillance System) | 18+ | NA | Other cancer; self-report | Prevalence | 8.50% | NA | NA | NA | NA | NA | NA | NA |
| Polonijo 2020 | United States | 90 | Inland Empire Transgender Health and Wellness Profile, 2015 | NA | NA | Cancer; self-report | Prevalence | 6.70% | NA | NA | NA | NA | NA | NA | NA |
| Sanfacon 2021 | United States | 74 | No study name provided | NA | Adults who were born or became deaf in both ears before 13 years of age | Cancer; self-report diagnosis | Prevalence | 2.70% | NA | NA | NA | NA | NA | NA | NA |
| Singer 2020 | United States | 3749 | BRFSS (Behavioral Risk Factor Surveillance System) | 18+ | NA | Skin cancer ever; self-report | Prevalence | NA | NA | NA | 6.00% | 6.10% | NA | NA | 6.80% |
| Smeaton 2020 | United States; Canada; Spain; Brazil; Peru; Haiti; Thailand; India; South Africa; Uganda; Zimbabwe; Botswana | 129 | REPRIEVE | 40-75 | PLHIV | History of cancer; self-report | Prevalence | NA | 5.00% | 0.00% | NA | NA | NA | NA | NA |
| Swartz 2021 | United States | 223 | No study name provided | 18+ | TWLHIV | Cancer; self-report of physician diagnosis | Prevalence | NA | NA | NA | 6.70% | NA | NA | NA | NA |
| Cardiovascular or cerebrovascular | | | | | | | | | | | | | | | |
| Abramovich 2020 | Canada | 2085 | No study name provided | NA | NA | Chronic heart failure; diagnosis | Prevalence | 0.40% | NA | NA | NA | NA | NA | NA | NA |
| Abramovich 2020 | Canada | 2085 | No study name provided | NA | NA | Hypertension; diagnosis | Prevalence | 6.10% | NA | NA | NA | NA | NA | NA | NA |
| Abramovich 2020 | Canada | 2085 | No study name provided | NA | NA | Myocardial infarction; diagnosis | Prevalence | 0.30% | NA | NA | NA | NA | NA | NA | NA |
| Antwi-Amoabeng 2020 | United States | 16555 | National Inpatient Sample | 18+ | Patients who underwent gender-affirming surgeries | Arrhythmia; diagnosis | Incidence | 3.70% | NA | NA | NA | NA | NA | NA | NA |
| Antwi-Amoabeng 2020 | United States | 16555 | National Inpatient Sample | 18+ | Patients who underwent gender-affirming surgeries | Hypertension; diagnosis | Prevalence | 26.40% | NA | NA | NA | NA | NA | NA | NA |
| Antwi-Amoabeng 2020 | United States | 16555 | National Inpatient Sample | 18+ | Patients who underwent gender-affirming surgeries | Stroke; diagnosis | Prevalence | 0.60% | NA | NA | NA | NA | NA | NA | NA |
| Balcerek 2021 | Australia | 296 | No study name provided | NA | Aging individuals | Hypertension (in patients aged >45yo); diagnosis | Prevalence | NA | 29.00% | NA | NA | NA | NA | NA | NA |
| Balcerek 2021 | Australia | 296 | No study name provided | NA | Aging individuals | Ischemic heart disease (in patients aged >45yo); diagnosis | Prevalence | NA | 5.00% | NA | NA | NA | NA | NA | NA |
| Balcerek 2021 | Australia | 296 | No study name provided | NA | Aging individuals | Cerebrovascular disease (in patients aged >45yo); diagnosis | Prevalence | NA | 4.00% | NA | NA | NA | NA | NA | NA |
| Balcerek 2021 | Australia | 296 | No study name provided | NA | Aging individuals | Venous thromboembolic disease; diagnosis | Prevalence | NA | 2.00% | NA | NA | NA | NA | NA | NA |
| Banks 2021 | United States | 470 | No study name provided | 17+ | People using GAHT | Stage 2 hypertension; diagnosis | Prevalence | 12.10% | 15.80% | 8.10% | NA | NA | NA | NA | NA |
| Bretherton 2021 | Australia | 928 | No study name provided | 18+ | NA | Blood clots (pulmonary embolus or deep vein thrombosis); self-report | Prevalence | 2.00% | NA | NA | NA | NA | NA | NA | NA |
| Bretherton 2021 | Australia | 928 | No study name provided | 18+ | NA | Stroke; self-report | Prevalence | 1.00% | NA | NA | NA | NA | NA | NA | NA |
| Bretherton 2021 | Australia | 928 | No study name provided | 18+ | NA | Ischemic heart disease; self-report | Prevalence | <1.0% | NA | NA | NA | NA | NA | NA | NA |
| Bretherton 2021 | Australia | 928 | No study name provided | 18+ | NA | Stroke; self-report | Prevalence | 1.00% | NA | NA | NA | NA | NA | NA | NA |
| Bretherton 2021 | Australia | 928 | No study name provided | 18+ | NA | Ischemic heart disease; self-report | Prevalence | <1.0% | NA | NA | NA | NA | NA | NA | NA |
| Cocchetti 2021 | Belgium; Italy; Norway | 309 | ENIGI endocrine study | 20-59 | NA | Hypertension diagnosis (at baseline); diagnosis | Prevalence | NA | NA | NA | 2.10% | 1.80% | NA | NA | NA |
| Denby 2021 | United States | 427 | No study name provided | 18+ | Patients seeking GAHT | Coronary artery disease; diagnosis | Prevalence | 1.60% | NA | NA | NA | NA | NA | NA | NA |
| Denby 2021 | United States | 427 | No study name provided | 18+ | Patients seeking GAHT | Congestive heart failure; diagnosis | Prevalence | 0.70% | NA | NA | NA | NA | NA | NA | NA |
| Denby 2021 | United States | 427 | No study name provided | 18+ | Patients seeking GAHT | Stroke or TIA; diagnosis | Prevalence | 0.50% | NA | NA | NA | NA | NA | NA | NA |
| Denby 2021 | United States | 427 | No study name provided | 18+ | Patients seeking GAHT | Hypertension; diagnosis | Prevalence | 24.10% | NA | NA | NA | NA | NA | NA | NA |
| DuBois 2020 | United States | 786 | BRFSS (Behavioral Risk Factor Surveillance System) | 18+ | NA | Heart attack; self-report | Prevalence | NA | NA | NA | 7.90% | 5.00% | NA | NA | 8.90% |
| DuBois 2020 | United States | 786 | BRFSS (Behavioral Risk Factor Surveillance System) | 18+ | NA | Heart disease; self-report | Prevalence | NA | NA | NA | 4.10% | 5.00% | NA | NA | 3.20% |
| Gabrick 2021 | United States | 137 | National Surgical Quality Improvement Program (NSQIP) | NA | Underwent breast augmentation | Hypertension requiring medication; diagnosis | Prevalence | NA | NA | NA | 8.20% | NA | NA | NA | NA |
| Gosiker 2020 | United States | 221 | No study name provided | NA | TW living with HIV | Treatment for hypertension; diagnosis | Prevalence | NA | NA | NA | 73.00% | NA | NA | NA | NA |
| Hawkins 2021 | United States | 81 | No study name provided | 18+ | Transmasculine patients on testosterone presenting for hysterectomy | Hypertension; diagnosis | Prevalence | NA | NA | 18.00% | NA | NA | NA | NA | NA |
| James 2020 | United States | 82 | Rochester Epidemiology Project | NA | Patients seeking gender-related healthcare | Hypertension; diagnosis | Prevalence | NA | 26.50% | 25.80% | NA | NA | NA | NA | NA |
| James 2020 | United States | 82 | Rochester Epidemiology Project | NA | Patients seeking gender-related healthcare | Myocardial infarction; diagnosis | Prevalence | NA | 0.00% | 3.20% | NA | NA | NA | NA | NA |
| Klaver 2020 | Netherlands | 192 | No study name provided | Adolescents | Treated with gonadotropin-releasing hormone agonists | High cholesterol at 22 years old; diagnosis | Prevalence | NA | NA | NA | 0.00% | 5.30% | NA | NA | NA |
| Lett 2020 | United States | 3151 | BRFSS (Behavioral Risk Factor Surveillance System) | NA | NA | Cardiovascular disease; self-report of physician diagnosis | Prevalence | Black: 17.9%; White: 13.5% | NA | NA | NA | NA | NA | NA | NA |
| Levit 2021 | Israel | 115 | No study name provided | NA | NA | Anaemia; NA | Prevalence | NA | NA | NA | 4.60% | 15.30% | NA | NA | NA |
| Madhavan 2020 | India | 200 | No study name provided | 18+ | NA | Hypertension; diagnosis | Prevalence | 16.00% | NA | NA | NA | NA | NA | NA | NA |
| Mohr 2021 | Switzerland | 98 | No study name provided | NA | NA | Hypertension; diagnosis | Prevalence | 13.30% | NA | NA | 13.20% | 13.30% | NA | NA | NA |
| Mohr 2021 | Switzerland | 98 | No study name provided | NA | NA | Hypertensive heart disease; diagnosis | Prevalence | 3.10% | NA | NA | 5.70% | 0.00% | NA | NA | NA |
| Mohr 2021 | Switzerland | 98 | No study name provided | NA | NA | Myocardial infarction; diagnosis | Prevalence | 2.00% | NA | NA | 1.90% | 2.20% | NA | NA | NA |
| Mohr 2021 | Switzerland | 98 | No study name provided | NA | NA | Coronary heart disease; diagnosis | Prevalence | 1.00% | NA | NA | 1.90% | 0.00% | NA | NA | NA |
| Mohr 2021 | Switzerland | 98 | No study name provided | NA | NA | Valvular heart disease; diagnosis | Prevalence | 1.00% | NA | NA | 1.90% | 0.00% | NA | NA | NA |
| Mullins 2021a. | United States | 611 | No study name provided | 13-24 | NA | Thrombosis; diagnosis | Prevalence | 0.50% | NA | NA | NA | NA | NA | NA | NA |
| Mullins 2021b. | United States | 611 | No study name provided | under 25 | Youth on GAHT | Venous thromboembolism; diagnosis | Prevalence | NA | NA | NA | 0.60% | 0.20% | NA | NA | NA |
| Mullins 2021b. | United States | 611 | No study name provided | under 25 | Youth on GAHT | Arterial stroke; diagnosis | Prevalence | NA | NA | NA | 0.60% | NA | NA | NA | NA |
| Nolan 2021 | Australia | 180 | No study name provided | NA | NA | Hypertension; diagnosis | Prevalence | NA | NA | 7.00% | NA | NA | NA | NA | NA |
| Nolan 2021 | Australia | 180 | No study name provided | NA | NA | High cholesterol; diagnosis | Prevalence | NA | NA | 13.00% | NA | NA | NA | NA | NA |
| Papadopulos 2020 | Germany | 47 | No study name provided | 18-57 | TW who underwent gender-confirming surgery | Hypertension; self-report | Prevalence | NA | NA | NA | 12.80% | NA | NA | NA | NA |
| Pharr 2021 | United States | 2827 | BRFSS (Behavioral Risk Factor Surveillance System) | 18+ | NA | High blood pressure; self-report | Prevalence | 31.30% | NA | NA | NA | NA | NA | NA | NA |
| Pharr 2021 | United States | 2827 | BRFSS (Behavioral Risk Factor Surveillance System) | 18+ | NA | High cholesterol; self-report | Prevalence | 30.80% | NA | NA | NA | NA | NA | NA | NA |
| Pharr 2021 | United States | 2827 | BRFSS (Behavioral Risk Factor Surveillance System) | 18+ | NA | Stroke; self-report | Prevalence | 6.50% | NA | NA | NA | NA | NA | NA | NA |
| Pharr 2021 | United States | 2827 | BRFSS (Behavioral Risk Factor Surveillance System) | 18+ | NA | Coronary heart disease; self-report | Prevalence | 7.10% | NA | NA | NA | NA | NA | NA | NA |
| Pharr 2021 | United States | 2827 | BRFSS (Behavioral Risk Factor Surveillance System) | 18+ | NA | Heart attack; self-report | Prevalence | 7.90% | NA | NA | NA | NA | NA | NA | NA |
| Polonijo 2020 | United States | 90 | Inland Empire Transgender Health and Wellness Profile, 2015 | NA | NA | High cholesterol; self-report | Prevalence | 32.20% | NA | NA | NA | NA | NA | NA | NA |
| Polonijo 2020 | United States | 90 | Inland Empire Transgender Health and Wellness Profile, 2015 | NA | NA | High blood pressure; self-report | Prevalence | 25.60% | NA | NA | NA | NA | NA | NA | NA |
| Poteat 2021a. | United States | 114 | TransPop | 18+ | NA | CVD; self-report diagnosis | Prevalence | 38.50% | NA | NA | 42.30% | 29.30% | NA | NA | 40.60% |
| Poteat 2021a. | United States | 114 | TransPop | 18+ | NA | Venous thromboembolism; self-report diagnosis | Prevalence | 7.80% | NA | NA | 6.80% | 2.10% | NA | NA | 18.00% |
| Pyra 2020 | United States | 4402 | No study name provided | 20-70 | NA | Thromboembolism on GAHT; diagnosis | Incidence | NA | NA | NA | 0.80% | 0.20% | NA | NA | NA |
| Pyra 2020 | United States | 4402 | No study name provided | 20-70 | NA | Hypertension on GAHT; diagnosis | Prevalence | NA | NA | NA | 2.10% | 1.50% | NA | NA | NA |
| Sanfacon 2021 | United States | 74 | No study name provided | NA | Adults who were born or became deaf in both ears before 13 years of age | Hypertension/high blood pressure; self-report diagnosis | Prevalence | 28.80% | NA | NA | NA | NA | NA | NA | 21.20% |
| Sanfacon 2021 | United States | 74 | No study name provided | NA | Adults who were born or became deaf in both ears before 13 years of age | Heart condition; self-report diagnosis | Prevalence | 5.50% | NA | NA | NA | NA | NA | NA | 3.00% |
| Sanfacon 2021 | United States | 74 | No study name provided | NA | Adults who were born or became deaf in both ears before 13 years of age | Stroke; self-report diagnosis | Prevalence | 2.70% | NA | NA | NA | NA | NA | NA | NA |
| Smeaton 2020 | United States; Canada; Spain; Brazil; Peru; Haiti; Thailand; India; South Africa; Uganda; Zimbabwe; Botswana | 129 | REPRIEVE | 40-75 | PLHIV | Hypertension ever; self-report | Prevalence | NA | 24.00% | 38.00% | NA | NA | NA | NA | NA |
| Swartz 2021 | United States | 223 | No study name provided | 18+ | TW PLHIV | Hypertension; self-report of physician diagnosis | Prevalence | NA | NA | NA | 13.90% | NA | NA | NA | NA |
| Swartz 2021 | United States | 223 | No study name provided | 18+ | TW PLHIV | Stroke; self-report of physician diagnosis | Prevalence | NA | NA | NA | 4.00% | NA | NA | NA | NA |
| vanHeesewijk 2021 | Netherlands | 37 | No study name provided | 55-70 | NA | Hypertension; blood pressure measured in study | Prevalence | NA | NA | NA | 50.00% | NA | NA | NA | NA |
| vanHeesewijk 2021 | Netherlands | 37 | No study name provided | 55-70 | NA | CVD ever; self-report | Prevalence | NA | NA | NA | 18.90% | NA | NA | NA | NA |
| Wang 2021a. | United States | 2890 | Musculoskeletal diagnosis (MSD) cohort | 18+ | NA | Hypertension; diagnosis | Prevalence | 33.20% | NA | NA | NA | NA | NA | NA | NA |
| Wang 2021a. | United States | 2890 | Musculoskeletal diagnosis (MSD) cohort | 18+ | NA | Coronary artery disease; diagnosis | Prevalence | 8.20% | NA | NA | NA | NA | NA | NA | NA |
| Waterschoot 2021 | Belgium | 74 | No study name provided | 18+ | Transmasculine patients who had metoidioplasty | Minor CVD; diagnosis | Prevalence | NA | NA | 5.40% | NA | NA | NA | NA | NA |
| Metabolic or endocrine | | | | | | | | | | | | | | | |
| Abramovich 2020 | Canada | 2085 | No study name provided | NA | NA | Diabetes; diagnosis | Prevalence | 5.50% | NA | NA | NA | NA | NA | NA | NA |
| Antwi-Amoabeng 2020 | United States | 16555 | National Inpatient Sample | 18+ | Patients who underwent gender-affirming surgeries | Diabetes; diagnosis | Prevalence | 10.10% | NA | NA | NA | NA | NA | NA | NA |
| Balcerek 2021 | Australia | 296 | No study name provided | NA | Aging individuals | Dyslipidaemia (in patients aged >45yo); diagnosis | Prevalence | NA | 16.00% | NA | NA | NA | NA | NA | NA |
| Balcerek 2021 | Australia | 296 | No study name provided | NA | Aging individuals | Diabetes (in patients aged >45yo); diagnosis | Prevalence | NA | 9.00% | NA | NA | NA | NA | NA | NA |
| Balcerek 2021 | Australia | 296 | No study name provided | NA | Aging individuals | Obesity (BMI >30kg/m^2) (in patients aged >45yo); diagnosis | Prevalence | NA | 20.00% | NA | NA | NA | NA | NA | NA |
| Banks 2021 | United States | 470 | No study name provided | 17+ | People using GAHT | Diabetes; diagnosis | Prevalence | 2.10% | 2.80% | 1.30% | NA | NA | NA | NA | NA |
| Bretherton 2021 | Australia | 928 | No study name provided | 18+ | NA | Diabetes mellitus; self-report | Prevalence | 3.00% | NA | NA | NA | NA | NA | NA | NA |
| Brown 2020 | United States | 88 | BRFSS (Behavioral Risk Factor Surveillance System) | 45+ | NA | Diagnosed with diabetes ever; self-report of physician diagnosis | Prevalence | 24.70% | NA | NA | NA | NA | NA | NA | NA |
| Cocchetti 2021 | Belgium; Italy; Norway | 309 | ENIGI endocrine study | 20-59 | NA | Diabetes diagnosis (at baseline); diagnosis | Prevalence | NA | NA | NA | 1.40% | 0.60% | NA | NA | NA |
| Denby 2021 | United States | 427 | No study name provided | 18+ | Patients seeking GAHT | Diabetes mellitus; diagnosis | Prevalence | 6.30% | NA | NA | NA | NA | NA | NA | NA |
| Denby 2021 | United States | 427 | No study name provided | 18+ | Patients seeking GAHT | Obesity (BMI>30); diagnosis | Prevalence | 35.00% | NA | NA | NA | NA | NA | NA | NA |
| Dinger 2020 | United States | 2487 | National College Health Assessment | 18-25 | College students | Obesity; self-report | Prevalence | Transgender: 18.8% | NA | NA | NA | NA | NA | NA | Non-binary, non-trans: 16.3% |
| Gabrick 2021 | United States | 137 | National Surgical Quality Improvement Program (NSQIP) | NA | Underwent breast augmentation | Diabetes; diagnosis | Prevalence | NA | NA | NA | 2.90% | NA | NA | NA | NA |
| Gosiker 2020 | United States | 221 | No study name provided | NA | TW PLHIV | Diabetes; diagnosis | Prevalence | NA | NA | NA | 8.00% | NA | NA | NA | NA |
| Hawkins 2021 | United States | 81 | No study name provided | 18+ | Transmasculine patients on testosterone presenting for hysterectomy | Obesity; diagnosis | Prevalence | NA | NA | 28.00% | NA | NA | NA | NA | NA |
| Hawkins 2021 | United States | 81 | No study name provided | 18+ | Transmasculine patients on testosterone presenting for hysterectomy | Diabetes; diagnosis | Prevalence | NA | NA | 4.00% | NA | NA | NA | NA | NA |
| James 2020 | United States | 82 | Rochester Epidemiology Project | NA | Patients seeking gender-related healthcare | Obesity; diagnosis | Prevalence | NA | 42.90% | 41.90% | NA | NA | NA | NA | NA |
| James 2020 | United States | 82 | Rochester Epidemiology Project | NA | Patients seeking gender-related healthcare | Type 2 diabetes; diagnosis | Prevalence | NA | 10.20% | 3.20% | NA | NA | NA | NA | NA |
| Kirby 2020 | United States | 26 | No study name provided | 18+ | College students | Obesity; self-report | Prevalence | 3.80% | NA | NA | NA | NA | NA | NA | NA |
| Klaver 2020 | Netherlands | 192 | No study name provided | Adolescents | Treated with gonadotropin-releasing hormone agonists | Obesity | Prevalence | NA | NA | NA | 9.90% | 6.60% | NA | NA | NA |
| Lett 2020 | United States | 3151 | BRFSS (Behavioral Risk Factor Surveillance System) | NA | NA | Diabetes; self-report of physician diagnosis | Prevalence | Black: 19.7%; White: 12.4% | NA | NA | NA | NA | NA | NA | NA |
| Levit 2021 | Israel | 115 | No study name provided | NA | NA | Hypothyroidism; diagnosis | Prevalence | NA | NA | NA | 6.90% | 4.20% | NA | NA | NA |
| Madhavan 2020 | India | 200 | No study name provided | 18+ | NA | Diabetes; self-report of physician diagnosis | Prevalence | 8.00% | NA | NA | NA | NA | NA | NA | NA |
| Madhavan 2020 | India | 200 | No study name provided | 18+ | NA | Obesity; self-report | Prevalence | 34.50% | NA | NA | NA | NA | NA | NA | NA |
| Martinson 2020 | United States | 1457 | No study name provided | NA | NA | Obesity; diagnosis | Prevalence | NA | 23.00% | 37.00% | NA | NA | NA | NA | NA |
| Mohr 2021 | Switzerland | 98 | No study name provided | NA | NA | Type I diabetes; diagnosis | Prevalence | 1.00% | NA | NA | 1.90% | 0.00% | NA | NA | NA |
| Mohr 2021 | Switzerland | 98 | No study name provided | NA | NA | Type II diabetes; diagnosis | Prevalence | 4.10% | NA | NA | 7.50% | 0.00% | NA | NA | NA |
| Mullins 2021b. | United States | 611 | No study name provided | under 25 | Youth on GAHT | Elevated BMI; diagnosis | Prevalence | Overweight: 24.2%; obese: 34.5% | NA | NA | NA | NA | NA | NA | NA |
| Pharr 2021 | United States | 2827 | BRFSS (Behavioral Risk Factor Surveillance System) | 18+ | NA | Prediabetes; self-report | Prevalence | 17.30% | NA | NA | NA | NA | NA | NA | NA |
| Pharr 2021 | United States | 2827 | BRFSS (Behavioral Risk Factor Surveillance System) | 18+ | NA | Diabetes; self-report | Prevalence | 15.40% | NA | NA | NA | NA | NA | NA | NA |
| Polonijo 2020 | United States | 90 | Inland Empire Transgender Health and Wellness Profile, 2015 | NA | NA | Diabetes; self-report | Prevalence | 6.70% | NA | NA | NA | NA | NA | NA | NA |
| Pyra 2020 | United States | 4402 | No study name provided | 20-70 | NA | Diabetes; diagnosis | Prevalence | NA | NA | NA | 3.30% | 3.00% | NA | NA | NA |
| Rothenberg 2021 | United States | 948 | No study name provided | 18+ | Top surgery patients | Obesity; diagnosis | Prevalence | 31.10% | NA | NA | NA | NA | NA | NA | NA |
| Sanfacon 2021 | United States | 74 | No study name provided | NA | Adults who were born or became deaf in both ears before 13 years of age | Diabetes; self-report diagnosis | Prevalence | 12.30% | NA | NA | NA | NA | NA | NA | 12.10% |
| Smeaton 2020 | United States; Canada; Spain; Brazil; Peru; Haiti; Thailand; India; South Africa; Uganda; Zimbabwe; Botswana | 129 | REPRIEVE | 40-75 | PLHIV | Diabetes ever; self-report | Prevalence | NA | 2.00% | 13.00% | NA | NA | NA | NA | NA |
| Suarez 2021 | United States | 131 | No study name provided | 21-64 | NA | Obesity; BMI calculated | Prevalence | NA | NA | 10.7%-35.6% (across number of adverse childhood experiences) | NA | NA | NA | NA | NA |
| Swartz 2021 | United States | 223 | No study name provided | 18+ | TW PLHIV | Diabetes or high blood sugar; self-report of physician diagnosis | Prevalence | NA | NA | NA | 4.50% | NA | NA | NA | NA |
| vanHeesewijk 2021 | Netherlands | 37 | No study name provided | 55-70 | NA | Diabetes mellitus; self-report | Prevalence | NA | NA | NA | 13.50% | NA | NA | NA | NA |
| Wang 2021a. | United States | 2890 | Musculoskeletal diagnosis (MSD) cohort | 18+ | NA | Diabetes; diagnosis | Prevalence | 14.30% | NA | NA | NA | NA | NA | NA | NA |
| Waterschoot 2021 | Belgium | 74 | No study name provided | 18+ | Transmasculine patients who had metoidioplasty | Diabetes; diagnosis | Prevalence | NA | NA | 0.00% | NA | NA | NA | NA | NA |
| Other chronic condition | | | | | | | | | | | | | | | |
| Abramovich 2020 | Canada | 2085 | No study name provided | NA | NA | Rheumatoid arthritis; diagnosis | Prevalence | 0.30% | NA | NA | NA | NA | NA | NA | NA |
| Abramovich 2020 | Canada | 2085 | No study name provided | NA | NA | Crohn's disease or colitis; diagnosis | Prevalence | 0.50% | NA | NA | NA | NA | NA | NA | NA |
| Antwi-Amoabeng 2020 | United States | 16555 | National Inpatient Sample | 18+ | Patients who underwent gender-affirming surgeries | Chronic kidney disease; diagnosis | Prevalence | 4.40% | NA | NA | NA | NA | NA | NA | NA |
| Antwi-Amoabeng 2020 | United States | 16555 | National Inpatient Sample | 18+ | Patients who underwent gender-affirming surgeries | Chronic liver disease; diagnosis | Prevalence | 5.10% | NA | NA | NA | NA | NA | NA | NA |
| Bretherton 2021 | Australia | 928 | No study name provided | 18+ | NA | Liver disease; self-report | Prevalence | 1.00% | NA | NA | NA | NA | NA | NA | NA |
| Bretherton 2021 | Australia | 928 | No study name provided | 18+ | NA | Kidney or renal disease; self-report | Prevalence | <1.0% | NA | NA | NA | NA | NA | NA | NA |
| Cicero 2020 | United States | 764 | BRFSS (Behavioral Risk Factor Surveillance System) | 18-79 | NA | 2+ chronic health conditions; self-report | Prevalence | NA | NA | NA | 36.70% | 33.80% | NA | NA | 40.90% |
| Denby 2021 | United States | 427 | No study name provided | 18+ | Patients seeking GAHT | Chronic kidney disease; diagnosis | Prevalence | 0.50% | NA | NA | NA | NA | NA | NA | NA |
| Denby 2021 | United States | 427 | No study name provided | 18+ | Patients seeking GAHT | Autoimmune disease; diagnosis | Prevalence | 4.00% | NA | NA | NA | NA | NA | NA | NA |
| Gava 2021 | Italy | 108 | No study name provided | 18+ | NA | 1+ chronic health conditions; self-report | Prevalence | 10.10% | NA | NA | NA | NA | NA | NA | NA |
| Hershner 2021 | United States; Canada | 4611 | American College Health Association-National College Health Assessment II | NA | College students | Insomnia; self-report of physician diagnosis | Prevalence | 13.3% (USA), 12.7% (Canada) | NA | NA | NA | NA | NA | NA | NA |
| Hershner 2021 | United States; Canada | 4611 | American College Health Association-National College Health Assessment II | NA | College students | Sleep disorders; self-report of physician diagnosis | Prevalence | 6.0% (USA) 7.3% (Canada) | NA | NA | NA | NA | NA | NA | NA |
| Imhof 2020 | United States | 214 | No study name provided | NA | NA | Atopic dermatitis; diagnosis | Prevalence | NA | NA | NA | 7.50% | NA | NA | NA | NA |
| James 2020 | United States | 82 | Rochester Epidemiology Project | NA | Patients seeking gender-related healthcare | Osteoporosis; diagnosis | Prevalence | NA | 2.00% | 0.00% | NA | NA | NA | NA | NA |
| Lett 2020 | United States | 3151 | BRFSS (Behavioral Risk Factor Surveillance System) | NA | NA | Kidney disease; self-report of physician diagnosis | Prevalence | Black: 4.5%; White: 2.9% | NA | NA | NA | NA | NA | NA | NA |
| Lett 2020 | United States | 3151 | BRFSS (Behavioral Risk Factor Surveillance System) | NA | NA | Arthritis; self-report of physician diagnosis | Prevalence | Black: 29.1%; White: 30.2% | NA | NA | NA | NA | NA | NA | NA |
| Levit 2021 | Israel | 115 | No study name provided | NA | NA | Fibromyalgia; diagnosis | Prevalence | 14.80% | NA | NA | 7.00% | 19.40% | NA | NA | NA |
| Lim 2021 | Australia | 134 | No study name provided | 18+ | NA | Physical, psychiatric, intellectual, cognitive and/or sensory disability.; self-report | Prevalence | 44.00% | NA | NA | NA | NA | NA | NA | NA |
| Mullins 2021a. | United States | 611 | No study name provided | 13-24 | NA | Migraine with aura; diagnosis | Prevalence | 4.60% | NA | NA | NA | NA | NA | NA | NA |
| Mullins 2021a. | United States | 611 | No study name provided | 13-24 | NA | Inflammatory bowel disease; diagnosis | Prevalence | 0.50% | NA | NA | NA | NA | NA | NA | NA |
| Mullins 2021a. | United States | 611 | No study name provided | 13-24 | NA | Juvenile rheumatoid arthritis; diagnosis | Prevalence | 0.20% | NA | NA | NA | NA | NA | NA | NA |
| Papadopulos 2020 | Germany | 47 | No study name provided | 18-57 | TW who underwent gender-confirming surgery | Psoriasis; self-report | Prevalence | NA | NA | NA | 4.30% | NA | NA | NA | NA |
| Pharr 2021 | United States | 2827 | BRFSS (Behavioral Risk Factor Surveillance System) | 18+ | NA | Arthritis; self-report | Prevalence | 25.50% | NA | NA | NA | NA | NA | NA | NA |
| Pharr 2021 | United States | 2827 | BRFSS (Behavioral Risk Factor Surveillance System) | 18+ | NA | Kidney diseases; self-report | Prevalence | 6.10% | NA | NA | NA | NA | NA | NA | NA |
| Radusky 2021a. | Argentina | 182 | No study name provided | 16+ | NA | Chronic conditions (heart or respiratory disease, diabetes, cancer, or hypertension); self-report | Prevalence | NA | 18.20% | 7.90% | NA | NA | NA | NA | 9.80% |
| Sanfacon 2021 | United States | 74 | No study name provided | NA | Adults who were born or became deaf in both ears before 13 years of age | Arthritis/rheumatism; self-report diagnosis | Prevalence | 16.20% | NA | NA | NA | NA | NA | NA | 12.10% |
| Sanfacon 2021 | United States | 74 | No study name provided | NA | Adults who were born or became deaf in both ears before 13 years of age | Cirrhosis/liver problems or kidney problems; self-report diagnosis | Prevalence | 7.00% | NA | NA | NA | NA | NA | NA | 6.10% |
| Smeaton 2020 | United States; Canada; Spain; Brazil; Peru; Haiti; Thailand; India; South Africa; Uganda; Zimbabwe; Botswana | 129 | REPRIEVE | 40-75 | PLHIV | Kidney disease; self-report | Prevalence | NA | 0.00% | 0.00% | NA | NA | NA | NA | NA |
| Swartz 2021 | United States | 223 | No study name provided | 18+ | TW PLHIV | Arthritis or rheumatism; self-report of physician diagnosis | Prevalence | NA | NA | NA | 9.00% | NA | NA | NA | NA |
| Swartz 2021 | United States | 223 | No study name provided | 18+ | TW PLHIV | Epilepsy or seizures; self-report of physician diagnosis | Prevalence | NA | NA | NA | 3.10% | NA | NA | NA | NA |
| Tantirattanakulchai 2021 | Thailand | 280 | No study name provided | 18+ | NA | Chronic disease; self-report | Prevalence | NA | NA | NA | 18.60% | NA | NA | NA | NA |
| Valente 2020 | United States | 330 | Project AFFIRM | 16+ | NA | Somatization P1W; Brief Symptom Inventory-18 | Prevalence | 26.40% | 28.60% | 24.20% | NA | NA | NA | NA | NA |
| Wang 2021a. | United States | 2890 | Musculoskeletal diagnosis (MSD) cohort | 18+ | NA | Pain; pain intensity numeric rating scale | Prevalence | Moderate: 18.2%; severe: 17.5% | NA | NA | NA | NA | NA | NA | NA |
| Respiratory | | | | | | | | | | | | | | | |
| Abramovich 2020 | Canada | 2085 | No study name provided | NA | NA | Asthma; diagnosis | Prevalence | 23.50% | NA | NA | NA | NA | NA | NA | NA |
| Abramovich 2020 | Canada | 2085 | No study name provided | NA | NA | COPD; diagnosis | Prevalence | 2.40% | NA | NA | NA | NA | NA | NA | NA |
| Antwi-Amoabeng 2020 | United States | 16555 | National Inpatient Sample | 18+ | Patients who underwent gender-affirming surgeries | Chronic lung disease; diagnosis | Prevalence | 20.00% | NA | NA | NA | NA | NA | NA | NA |
| Bretherton 2021 | Australia | 928 | No study name provided | 18+ | NA | Emphysema; self-report | Prevalence | <1.0% | NA | NA | NA | NA | NA | NA | NA |
| Denby 2021 | United States | 427 | No study name provided | 18+ | Patients seeking GAHT | COPD; diagnosis | Prevalence | 0.90% | NA | NA | NA | NA | NA | NA | NA |
| Denby 2021 | United States | 427 | No study name provided | 18+ | Patients seeking GAHT | Asthma; diagnosis | Prevalence | 18.70% | NA | NA | NA | NA | NA | NA | NA |
| Lett 2020 | United States | 3151 | BRFSS (Behavioral Risk Factor Surveillance System) | NA | NA | Pulmonary disease; self-report of physician diagnosis | Prevalence | Black: 24.2%; White: 23.8% | NA | NA | NA | NA | NA | NA | NA |
| Niforatos 2020 | United States | 10160 | Explorys, Inc Database | NA | NA | Bacterial pneumonia; diagnosis | Prevalence | 25.00% | NA | NA | NA | NA | NA | NA | NA |
| Niforatos 2020 | United States | 10160 | Explorys, Inc Database | NA | NA | Pneumocystis pneumonia; diagnosis | Prevalence | 5.00% | NA | NA | NA | NA | NA | NA | NA |
| Papadopulos 2020 | Germany | 47 | No study name provided | 18-57 | TW who underwent gender-confirming surgery | Bronchial asthma; self-report | Prevalence | NA | NA | NA | 4.30% | NA | NA | NA | NA |
| Peitzmeier 2021 | Global | 1800 | Binding Health Project | 18+ | AFAB or intersex individuals who had ever engaged in chest binding | Respiratory infection; self-report | Prevalence | NA | NA | Ever at P12M binding: 2.9%; Ever at P10Y binding: 11.8% | NA | NA | NA | NA | NA |
| Pharr 2021 | United States | 2827 | BRFSS (Behavioral Risk Factor Surveillance System) | 18+ | NA | Asthma current; self-report | Prevalence | 20.10% | NA | NA | NA | NA | NA | NA | NA |
| Pharr 2021 | United States | 2827 | BRFSS (Behavioral Risk Factor Surveillance System) | 18+ | NA | COPD current; self-report | Prevalence | 9.70% | NA | NA | NA | NA | NA | NA | NA |
| Polonijo 2020 | United States | 90 | Inland Empire Transgender Health and Wellness Profile, 2015 | NA | NA | Asthma; self-report | Prevalence | 35.60% | NA | NA | NA | NA | NA | NA | NA |
| Sanfacon 2021 | United States | 74 | No study name provided | NA | Adults who were born or became deaf in both ears before 13 years of age | Chronic respiratory condition (chronic lung disease/asthma/emphysema/chronic bronchitis); self-report diagnosis | Prevalence | 20.30% | NA | NA | NA | NA | NA | NA | 25.00% |
| Swartz 2021 | United States | 223 | No study name provided | 18+ | TWLHIV | COPD; self-report of physician diagnosis | Prevalence | NA | NA | NA | 13.90% | NA | NA | NA | NA |
| Swartz 2021 | United States | 223 | No study name provided | 18+ | TWLHIV | Asthma; self-report of physician diagnosis | Prevalence | NA | NA | NA | 31.40% | NA | NA | NA | NA |
| Swartz 2021 | United States | 223 | No study name provided | 18+ | TWLHIV | Pneumonia; self-report of physician diagnosis | Prevalence | NA | NA | NA | 14.80% | NA | NA | NA | NA |
| Wang 2021a. | United States | 2890 | Musculoskeletal diagnosis (MSD) cohort | 18+ | NA | COPD; diagnosis | Prevalence | 7.10% | NA | NA | NA | NA | NA | NA | NA |
| Infectious disease | | | | | | | | | | | | | | | |
| HIV | | | | | | | | | | | | | | | |
| Abramovich 2020 | Canada | 2085 | No study name provided | NA | NA | HIV; diagnosis | Prevalence | 1.60% | NA | NA | NA | NA | NA | NA | NA |
| Aguilar 2020 | Paraguay | 304 | No study name provided | 15+ | NA | HIV; test administered as part of study | Prevalence | NA | NA | NA | 24.80% | NA | NA | NA | NA |
| Antebi-Gruszka 2020 | United States | 192 | No study name provided | 18+ | TMSM | HIV; self-report | Prevalence | NA | NA | NA | NA | 2.10% | NA | NA | NA |
| Aspia 2020 | Indonesia | 867 | IBBS | 15+ | NA | HIV; diagnosis | Prevalence | NA | NA | NA | 26.10% | NA | NA | NA | NA |
| Batista 2020 | Brazil | 95 | No study name provided | NA | NA | HIV/AIDS; diagnosis | Prevalence | NA | NA | NA | 18.00% | NA | NA | NA | NA |
| Bretherton 2021 | Australia | 928 | No study name provided | 18+ | NA | HIV/AIDS; self-report | Prevalence | <1.0% | NA | NA | NA | NA | NA | NA | NA |
| Burns 2021 | United States | 37 | No study name provided | NA | Patients with cancer | HIV; diagnosis | Prevalence | 8.10% | NA | NA | NA | NA | NA | NA | NA |
| Cardona-Arias 2020 | Colombia | 41 | No study name provided | NA | NA | HIV; diagnosis | Prevalence | 2.40% | NA | NA | NA | NA | NA | NA | NA |
| Clark 2021 | Peru | 205 | No study name provided | 18+ | HIV-negative or status unknown TW in Peru who reported CAI | HIV at baseline; diagnosis | Prevalence | NA | NA | NA | 16.10% | NA | NA | NA | NA |
| Deutsch 2020 | United States | 132 | No study name provided | 21-64 | NA | HIV; a mix of rapid HIV testing, medical records, and self-report | Prevalence | 0.80% | NA | NA | NA | NA | NA | NA | NA |
| Druckler 2020 | Netherlands | 40 | No study name provided | NA | SW in Amsterdam red light district | HIV; diagnosis | Prevalence | NA | NA | NA | 10.00% | NA | NA | NA | NA |
| Fan 2021 | China | 220 | No study name provided | 18+ | SW | HIV; self-report | Prevalence | NA | NA | NA | 16.80% | NA | NA | NA | NA |
| Fan 2021 | China | 220 | No study name provided | 18+ | SW | HIV; finger-prick HIV rapid test | Prevalence | NA | NA | NA | 9.10% | NA | NA | NA | NA |
| Fearon 2020 | South Africa | 67 | No study name provided | 18+ | AMAB who had sex with a man P12M | HIV; diagnosis | Prevalence | 19.40% | 15.70% | 0.30% | NA | NA | NA | NA | 3.40% |
| Fein 2021 | United States | 79 | No study name provided | 18+ | NA | HIV; self-report | Prevalence | NA | NA | NA | 9.00% | 0.00% | NA | NA | NA |
| Frola 2020 | Argentina | 260 | No study name provided | 14+ | NA | HIV; diagnosis | Prevalence | NA | NA | NA | 19.60% | NA | NA | NA | NA |
| Hernandez 2021 | United States | 201 | National HIV Behavioral Surveillance (NHBS) Transgender Women Study | 18+ | NA | HIV; self-report of physician diagnosis | Prevalence | NA | NA | NA | 42.30% | NA | NA | NA | NA |
| Hickson 2020 | Europe | 1047 | The European MSM Internet Survey 2017 | Legal age of consent | NA | HIV; self-report | Prevalence | NA | NA | NA | NA | AMAB trans men: 7.1%  AFAB trans men: 1.0%  AFAB men: 3.5% | NA | NA | NA |
| Hung 2020 | Peru | 31 | No study name provided | 18+ | NA | HIV; diagnosis | Prevalence | NA | NA | NA | 29.00% | NA | NA | NA | NA |
| Indrawati 2020 | Indonesia | 113 | IBBS (Integrated Biological and Behavioral Surveillance) | 15+ | Never tested for HIV | HIV; testing as part of study | Prevalence | NA | NA | NA | 17.70% | NA | NA | NA | NA |
| Jacoby 2021 | United States | 295 | No study name provided | NA | NA | HIV; diagnosis | Prevalence | 3.80% | NA | NA | NA | NA | NA | NA | NA |
| James 2020 | United States | 82 | Rochester Epidemiology Project | NA | Patients seeking gender-related healthcare | HIV; diagnosis | Prevalence | NA | 0.00% | 0.00% | NA | NA | NA | NA | NA |
| Klemmer 2021 | United States | 233 | No study name provided | 18+ | NA | HIV; diagnosis | Prevalence | 36.00% | NA | NA | 36.00% | NA | NA | NA | NA |
| Komarudin 2020 | Indonesia | 995 | Indonesian Ministry of Health‚Äôs Integrated Biological and Behavioral Survey (IBBS) | 15+ | NA | HIV; test result | Prevalence | NA | 24.80% | NA | NA | NA | NA | NA | NA |
| Kota 2020 | United States | 92 | No study name provided | 18-65 | NA | HIV; self-report | Prevalence | NA | NA | NA | 54.30% | NA | NA | NA | NA |
| Logie 2020b. | Jamaica | 101 | No study name provided | NA | NA | HIV; self-report | Prevalence | NA | NA | NA | 36.80% | NA | NA | NA | NA |
| Long 2020 | Peru | 142 | Sabes | 18+ | NA | HIV; self-report of physician diagnosis | Prevalence | NA | NA | NA | 14.80% | NA | NA | NA | NA |
| LoSchiavo 2020 | United States | 43 | P18 Viral | Young adults | NA | HIV; test result | Prevalence | 14.00% | NA | NA | NA | NA | NA | NA | NA |
| Luz 2021 | Brazil | 489 | Transcendendo | 18+ | NA | HIV; diagnosis | Prevalence | NA | NA | NA | 41.90% | NA | NA | NA | NA |
| Lyons 2020 | Senegal | 59 | No study name provided | 18+ | NA | HIV; diagnosis | Prevalence | NA | NA | NA | 44.80% | NA | NA | NA | NA |
| Madhavan 2020 | India | 200 | No study name provided | 18+ | NA | HIV; self-report of physician diagnosis | Prevalence | 2.50% | NA | NA | NA | NA | NA | NA | NA |
| McFarland 2020 | United States | 415 | TransNational Study in San Francisco | 18+ | NA | HIV; diagnosis | Incidence | NA | NA | NA | 1.3 per 100PY | NA | NA | NA | NA |
| MuesesMar√≠n 2020 | Colombia | 59 | No study name provided | NA | NA | HIV; HIV test | Prevalence | 15.20% | NA | NA | 15.20% | NA | NA | NA | NA |
| Mustanski 2020 | United States | 32 | RADAR | 16-20 | NA | HIV; HIV test | Incidence | NA | NA | NA | 2.4 per 100 PY | NA | NA | NA | NA |
| Nadoushan 2021 | Iran | 58 | No study name provided | NA | NA | HIV; diagnosis | Prevalence | 0.00% | NA | NA | NA | NA | NA | NA | NA |
| Nanthaprut 2021 | Thailand | 830 | No study name provided | 18+ | NA | HIV; HIV test | Prevalence | NA | NA | NA | 5.50% | NA | NA | NA | NA |
| Niforatos 2020 | United States | 10160 | Explorys, Inc Database | NA | NA | HIV; diagnosis | Prevalence | 3.90% | NA | NA | NA | NA | NA | NA | NA |
| Passaro 2020a. | Peru | 120 | No study name provided | 18+ | NA | HIV; lab test | Prevalence | NA | NA | NA | 12.50% | NA | NA | NA | NA |
| Pines 2021 | Mexico | 98 | Proyecto Enlaces | 18+ | People who had anal sex with a cisgender man or TW in P4M | HIV; lab test | Prevalence | NA | NA | NA | 19.40% | NA | NA | NA | NA |
| Pitasi 2020 | United States | 1877 | Men Who Have Sex with Men (MSM) Testing Initiative (MTI) | NA | NA | HIV; diagnosis | Prevalence | NA | NA | NA | 4.60% | NA | NA | NA | NA |
| Poteat 2020a. | South Africa | 213 | No study name provided | 18+ | NA | HIV; self-report | Prevalence | NA | NA | NA | 31.00% | NA | NA | NA | NA |
| Poteat 2020c. | United States | 1020 | LITE (American Cohort to Study HIV Acquisition Among Transgender Women in High Risk Areas) | 18+ | NA | HIV; serology | Prevalence | NA | NA | NA | 27.00% | NA | NA | NA | NA |
| Prescott 2020 | United States | 187 | SHINE | 16-24 | NA | HIV; self-report | Prevalence | NA | NA | NA | 19.20% | NA | NA | NA | NA |
| Pyra 2020 | United States | 4402 | No study name provided | 20-70 | NA | HIV; diagnosis | Prevalence | NA | NA | NA | 13.40% | 0.70% | NA | NA | NA |
| Radusky 2021a. | Argentina | 182 | No study name provided | 16+ | NA | HIV; self-report | Prevalence | NA | 33.30% | 3.00% | NA | NA | NA | NA | 22.70% |
| Rajan 2020 | India | 3325 | IBBS (Integrated Biological and Behavioural Surveillance) | 15+ | NA | HIV; diagnosis | Prevalence | NA | NA | NA | 9.50% | NA | NA | NA | NA |
| Ramadhani 2020 | Nigeria | 311 | TRUST/RV368 cohort | Abuja: 16+; Lagos: 18+ | NA | HIV; diagnosis | Prevalence | NA | NA | NA | 59.40% | NA | NA | NA | NA |
| Ramadhani 2020 | Nigeria | 311 | TRUST/RV368 cohort | Abuja: 16+; Lagos: 18+ | NA | HIV; diagnosis | Incidence | NA | NA | NA | 23.8 per 100 PY | NA | NA | NA | 16.0 per 100 PY |
| Restar 2020c. | Philippines | 139 | The #ParaSaAtin study | 18+ | TW who had CAI with a cis male partner in P12M | HIV; self-report | Prevalence | 33.60% | NA | NA | 33.60% | NA | NA | NA | NA |
| Rich 2021 | Canada | 188 | Population Data BC (PopDataBC); Drug Treatment Program (DTP); Ministry of Health | 19+ | NA | HIV; lab confirmed diagnosis | Prevalence | 27.10% | NA | NA | NA | NA | NA | NA | NA |
| Robbins 2020 | Nigeria | 251 | No study name provided | Abuja: 16+; Lagos: 18+ | TM MSM | HIV; diagnosis | Prevalence | NA | NA | NA | 71.10% | NA | 63.70% | NA | NA |
| Rocha 2020 | Brazil | 386 | Divas Research | NA | NA | HIV; diagnosis | Prevalence | NA | NA | NA | 38.00% | NA | NA | NA | NA |
| Rodriguez-Hart 2021 | United States | 6335 | No study name provided | NA | Medicaid beneficiaries | HIV; diagnosis | Prevalence | 28.00% | NA | NA | NA | NA | NA | NA | NA |
| Rouhani 2021 | United States | 42 | Sapphire Study | 15+ | Street-based SW | HIV; self-report diagnosis | Prevalence | NA | NA | NA | 46.60% | NA | NA | NA | NA |
| Rutherford 2021 | Canada | 340 | Sex Now Survey | 15+ | NA | HIV; self-report diagnosis | Prevalence | 5.10% | NA | NA | NA | NA | NA | NA | 5.30% |
| Sandfort 2021 | Kenya; Malawi; South Africa | 53 | HIV Prevention Trials Network (HPTN) 075 | 18-44 | People who had receptive anal intercourse with a cis male partner in P3M | HIV; diagnosis | Incidence | NA | NA | NA | 8.4 per 100 PY | NA | NA | NA | NA |
| Santos 2021 | Brazil | 415 | No study name provided | NA | NA | HIV; self-report | Prevalence | 20.50% | 27.60% | NA | 15.70% | NA | NA | NA | NA |
| Sevelius 2020 | United States | 77 | Sheroes | 18+ | TW reporting condomless sex in P6M | HIV; self-report | Prevalence | NA | NA | NA | 45.00% | NA | NA | NA | NA |
| She 2021a. | China | 235 | No study name provided | 18+ | TW SW | HIV; self-report | Prevalence | NA | NA | NA | Sample 1: 20.6%; Sample 2: 22.9% | NA | NA | NA | NA |
| She 2021b. | China | 204 | No study name provided | 18+ | TW SW | HIV; self-report | Prevalence | NA | NA | NA | 18.10% | NA | NA | NA | NA |
| Silva 2021 | Brazil | 189 | No study name provided | NA | NA | HIV; self-report | Prevalence | 13.20% | NA | NA | 21.60% | 1.30% | NA | NA | NA |
| Sok 2020 | Cambodia | 1375 | No study name provided | 18+ | NA | HIV; self-report | Prevalence | NA | NA | NA | 1.20% | NA | NA | NA | NA |
| Storm 2020 | Nepal | 173 | No study name provided | 16+ | NA | HIV; tested in study | Prevalence | NA | NA | NA | 13.00% | NA | NA | NA | NA |
| Teixeira 2021 | Brazil | 458 | No study name provided | 18+ | Individuals seeking HIV testing | HIV; diagnosis | Prevalence | NA | NA | NA | Recent infection: 1.7%; non-recent infection: 7.9% | NA | NA | NA | NA |
| Teixeira 2021 | Brazil | 458 | No study name provided | 18+ | Individuals seeking HIV testing | Annualized HIV incidence; diagnosis | Incidence | NA | NA | NA | 9.20% | NA | NA | NA | NA |
| TwahirwaRwema 2020 | Rwanda | 106 | No study name provided | 18+ | NA | HIV; tested in study | Prevalence | NA | NA | NA | 9.40% | NA | NA | NA | NA |
| Uaamnuichai 2021 | Thailand | 62 | No study name provided | NA | TW with neovagina | HIV; diagnosis | Prevalence | NA | NA | NA | 1.80% | NA | NA | NA | NA |
| Veronese 2020 | Myanmar | 214 | No study name provided | 15+ | NA | HIV; tested in study | Prevalence | NA | NA | NA | 19.20% | NA | NA | NA | NA |
| Vi 2020 | Vietnam | 456 | No study name provided | 18+ | NA | HIV; self-report or rapid tested in study | Prevalence | NA | NA | NA | 16.50% | NA | NA | NA | NA |
| Wang 2020b. | China | 397 | No study name provided | 19+ | TW SW | HIV; self-report | Prevalence | NA | NA | NA | 19.10% | NA | NA | NA | NA |
| Wang 2021b. | China | 198 | No study name provided | 18+ | Sexual intercourse in P6M | HIV; self-report | Prevalence | NA | NA | NA | 24.70% | NA | NA | NA | NA |
| Wansom 2021 | Thailand | 358 | No study name provided | 18-35 | High risk for HIV | HIV; tested in study | Incidence | NA | NA | NA | 3.8 per 100PY | NA | NA | NA | NA |
| Wesson 2021 | United States | 456 | The Trans*National Study | 18+ | NA | HIV; tested in study | Prevalence | 32.10% | NA | NA | 32.10% | NA | NA | NA | NA |
| Williams 2021a. | United States | 1392 | No study name provided | 18+ | Veterans in VA care with documented unhealthy alcohol use | HIV; diagnosis | Prevalence | 2.50% | NA | NA | NA | NA | NA | NA | NA |
| Williams 2021b. | United States | 8619 | No study name provided | 18+ | Veterans in VA care | HIV; diagnosis | Prevalence | 1.90% | NA | NA | NA | NA | NA | NA | NA |
| Wilson 2021a. | Brazil | 345 | Transcender | 18+ | NA | HIV; tested in study | Prevalence | NA | NA | NA | Youth: 24.5%; adults: 47.6% | NA | NA | NA | NA |
| Wilson 2021c. | Nepal | 200 | Sweekar | 18+ | NA | HIV test; rapid test | Prevalence | 11.30% | NA | NA | NA | NA | NA | NA | NA |
| Yunihastuti 2020 | Indonesia; Malaysia; Thailand | 48 | MSM-VCT; ANSAP | 18+ | South-east Asian MSM and TW | HIV; diagnosis | Prevalence | NA | NA | NA | 14.90% | NA | NA | NA | NA |
| Other infectious disease | | | | | | | | | | | | | | | |
| Buspavanich 2021 | Germany | 187 | No study name provided | 18+ | NA | COVID-19 infection ever; self-report | Prevalence | NA | NA | NA | 0.00% | 0.00% | NA | NA | 0.00% |
| Gava 2021 | Italy | 108 | No study name provided | 18+ | NA | Tested positive for COVID-19; self-report | Prevalence | 3.70% | NA | NA | NA | NA | NA | NA | NA |
| Hernandez 2021 | United States | 201 | National HIV Behavioral Surveillance (NHBS) Transgender Women Study | 18+ | NA | Hepatitis C; diagnosis | Prevalence | NA | NA | NA | 23.90% | NA | NA | NA | NA |
| Imhof 2020 | United States | 214 | No study name provided | NA | NA | Infections (candidal intertrigo, tinea versicolor); diagnosis | Prevalence | NA | NA | NA | NA | 8.20% | NA | NA | NA |
| Jones 2021 | United Kingdom | 161 | No study name provided | 16-25 | NA | Suspected COVID-19; self-report | Prevalence | 9.30% | NA | NA | NA | NA | NA | NA | NA |
| Kidd 2021b. | United States | 208 | Project AFFIRM | 18+ | NA | COVID-19; self-report | Prevalence | 0.50% | NA | NA | NA | NA | NA | NA | NA |
| Martino 2021 | United States | 211 | No study name provided | 18+ | NA | COVID-19; self-report of physician diagnosis | Prevalence | 12.10% | NA | NA | NA | NA | NA | NA | 4.80% |
| Niforatos 2020 | United States | 10160 | Explorys, Inc Database | NA | NA | Oral candidiasis; diagnosis | Prevalence | 15.00% | NA | NA | NA | NA | NA | NA | NA |
| Niforatos 2020 | United States | 10160 | Explorys, Inc Database | NA | NA | Tuberculosis; diagnosis | Prevalence | 8.00% | NA | NA | NA | NA | NA | NA | NA |
| Rutherford 2021 | Canada | 340 | Sex Now Survey | 15+ | NA | Hepatitis C ever; self-report diagnosis | Prevalence | NA | NA | 1.40% | NA | NA | NA | NA | 2.00% |
| Smeaton 2020 | United States; Canada; Spain; Brazil; Peru; Haiti; Thailand; India; South Africa; Uganda; Zimbabwe; Botswana | 129 | REPRIEVE | 40-75 | PLHIV | Chronic active Hepatitis C; self-report | Prevalence | NA | 4.10% | 0.00% | NA | NA | NA | NA | NA |
| Swartz 2021 | United States | 223 | No study name provided | 18+ | TW PLHIV | Hepatitis C; self-report of physician diagnosis | Prevalence | NA | NA | NA | 10.80% | NA | NA | NA | NA |
| Wang 2021a. | United States | 2890 | Musculoskeletal diagnosis (MSD) cohort | 18+ | NA | Hepatitis C; diagnosis | Prevalence | 4.00% | NA | NA | NA | NA | NA | NA | NA |
| Williams 2021a. | United States | 1392 | No study name provided | 18+ | Veterans in VA care with documented unhealthy alcohol use | Hepatitis C; diagnosis | Prevalence | 6.00% | NA | NA | NA | NA | NA | NA | NA |
| Williams 2021b. | United States | 8619 | No study name provided | 18+ | Veterans in VA care | Hepatitis C; diagnosis | Prevalence | 3.50% | NA | NA | NA | NA | NA | NA | NA |
| Other sexually transmitted infections | | | | | | | | | | | | | | | |
| Aspia 2020 | Indonesia | 867 | IBBS | 15+ | NA | STI history; testing as part of the study | Prevalence | NA | NA | NA | 22.10% | NA | NA | NA | NA |
| Clark 2021 | Peru | 205 | No study name provided | 18+ | TW in Peru who reported CAI and denied previously testing positive for HIV | Syphilis at baseline; diagnosis | Prevalence | NA | NA | NA | 18.00% | NA | NA | NA | NA |
| Crowell 2020 | Nigeria | 63 | No study name provided | 18+ | Intercourse with a male partner P12M | Anorectal Mycoplasma Genitalium; Aptima MG transcription-mediated amplification assay | Both | NA | NA | NA | 43.6%; 9.7 per 100PY | NA | NA | NA | NA |
| Crowell 2020 | Nigeria | 63 | No study name provided | 18+ | Intercourse with a male partner P12M | Urogenital Mycoplasma Genitalium; Aptima MG transcription-mediated amplification assay | Both | NA | NA | NA | 4.8%; 1.8 per 100PY | NA | NA | NA | NA |
| Deutsch 2020 | United States | 132 | No study name provided | 21-64 | NA | Current cervical hr-HPV infection; provider-collected cervical HPV DNA assay | Prevalence | 15.90% | NA | NA | NA | NA | NA | NA | NA |
| Druckler 2020 | Netherlands | 40 | No study name provided | NA | SW in Amsterdam red light district | Any bacterial STI positivity at study visit; diagnosis | Prevalence | NA | NA | NA | 25.00% | NA | NA | NA | NA |
| Druckler 2020 | Netherlands | 40 | No study name provided | NA | SW in Amsterdam red light district | Chlamydia (non LGV) positivity at study visit; diagnosis | Prevalence | NA | NA | NA | 22.50% | NA | NA | NA | NA |
| Druckler 2020 | Netherlands | 40 | No study name provided | NA | SW in Amsterdam red light district | Gonorrhea positivity at study visit; diagnosis | Prevalence | NA | NA | NA | 2.50% | NA | NA | NA | NA |
| Druckler 2020 | Netherlands | 40 | No study name provided | NA | SW in Amsterdam red light district | Positive syphilis serology (TPPA) at study visit; diagnosis | Prevalence | NA | NA | NA | 55.00% | NA | NA | NA | NA |
| Fein 2021 | United States | 79 | No study name provided | 18+ | NA | STI ever; self-report diagnosis | Prevalence | NA | NA | NA | 20.00% | 8.00% | NA | NA | NA |
| Ferlatte 2020a. | Canada | 209 | Sex Now Survey (2015) | NA | TMSM | STI; self-report of physician diagnosis | Prevalence | NA | NA | NA | NA | 5.70% | NA | NA | NA |
| Frola 2020 | Argentina | 260 | No study name provided | 14+ | NA | STI ever; self-report | Prevalence | NA | NA | NA | 32.30% | NA | NA | NA | NA |
| Goldstein 2020 | United States | 314 | No study name provided | 21+ | TM without hysterectomy | High-risk HPV; clinician-collected swab (n=28) | Prevalence | NA | NA | 17.90% | NA | NA | NA | NA | NA |
| Heller 2021 | United States | 659 | National College Health Assessment | 18+ | College students | STI P12M; self-report | Prevalence | NA | NA | NA | 20.00% | 4.00% | NA | NA | 1.10% |
| Hickson 2020 | Europe | 1047 | The European MSM Internet Survey 2017 | Legal age of consent | NA | Syphilis diagnosis P12M; self-report of physician diagnosis | Prevalence | NA | NA | NA | NA | AMAB trans men: 4.5%  AFAB trans men: 0.6%  AFAB men: 2.3% | NA | NA | NA |
| Hickson 2020 | Europe | 1047 | The European MSM Internet Survey 2017 | Legal age of consent | NA | Gonorrhoea diagnosis P12M; self-report of physician diagnosis | Prevalence | NA | NA | NA | NA | AMAB trans men: 2.0%  AFAB trans man: 2.9%  AFAB men: 1.7% | NA | NA | NA |
| Hung 2020 | Peru | 31 | No study name provided | 18+ | NA | Syphilis; diagnosis | Prevalence | NA | NA | NA | 54.80% | NA | NA | NA | NA |
| Hung 2020 | Peru | 31 | No study name provided | 18+ | NA | Chlamydia ever; diagnosis | Prevalence | NA | NA | NA | 3.20% | NA | NA | NA | NA |
| Hung 2020 | Peru | 31 | No study name provided | 18+ | NA | Gonorrhea ever; diagnosis | Prevalence | NA | NA | NA | 3.20% | NA | NA | NA | NA |
| Hung 2020 | Peru | 31 | No study name provided | 18+ | NA | Hepatitis B ever; diagnosis | Prevalence | NA | NA | NA | 0.00% | NA | NA | NA | NA |
| Hung 2020 | Peru | 31 | No study name provided | 18+ | NA | Genital herpes ever; diagnosis | Prevalence | NA | NA | NA | 6.50% | NA | NA | NA | NA |
| Hung 2020 | Peru | 31 | No study name provided | 18+ | NA | HPV ever; diagnosis | Prevalence | NA | NA | NA | 3.20% | NA | NA | NA | NA |
| Indrawati 2020 | Indonesia | 113 | IBBS (Integrated Biological and Behavioral Surveillance) | 15+ | Never tested for HIV | Syphilis | Prevalence | NA | NA | NA | 13.30% | NA | NA | NA | NA |
| Indrawati 2020 | Indonesia | 113 | IBBS (Integrated Biological and Behavioral Surveillance) | 15+ | Never tested for HIV | Gonorrhea or chlamydia | Prevalence | NA | NA | NA | 25.00% | NA | NA | NA | NA |
| James 2020 | United States | 82 | Rochester Epidemiology Project | NA | Patients seeking gender-related healthcare | Non-HIV STI; diagnosis | Prevalence | NA | 8.20% | 9.70% | NA | NA | NA | NA | NA |
| Komarudin 2020 | Indonesia | 995 | Indonesian Ministry of Health‚Äôs Integrated Biological and Behavioral Survey (IBBS) | 15+ | NA | STI symptoms P12M; self-report | Prevalence | HIV+: 34.8%; HIV-: 65.2 | HIV+: 34.8%; HIV-: 65.2% | NA | NA | NA | NA | NA | NA |
| Komarudin 2020 | Indonesia | 995 | Indonesian Ministry of Health‚Äôs Integrated Biological and Behavioral Survey (IBBS) | 15+ | NA | Syphilis; test result | Prevalence | HIV+: 31.8%; HIV-: 68.2% | HIV+: 31.8%; HIV-: 68.2% | NA | NA | NA | NA | NA | NA |
| Long 2020 | Peru | 142 | Sabes | 18+ | NA | Active untreated syphilis | Prevalence | NA | NA | NA | 8.50% | NA | NA | NA | NA |
| LoSchiavo 2020 | United States | 43 | P18 Viral | Young adults | NA | HPV; test result | Prevalence | NA | NA | NA | 45.50% | NA | NA | NA | 68.80% |
| LoSchiavo 2020 | United States | 43 | P18 Viral | Young adults | NA | HSV-1/2; test result | Prevalence | 55.80% | NA | NA | NA | NA | NA | NA | NA |
| LoSchiavo 2020 | United States | 43 | P18 Viral | Young adults | NA | STI P6M; self-report of physician diagnosis | Prevalence | 34.90% | NA | NA | NA | NA | NA | NA | NA |
| LoSchiavo 2020 | United States | 43 | P18 Viral | Young adults | NA | STI symptoms ever; self-report | Prevalence | 79.10% | NA | NA | NA | NA | NA | NA | NA |
| LoSchiavo 2020 | United States | 43 | P18 Viral | Young adults | NA | STI symptoms P12M; self-report | Prevalence | 55.80% | NA | NA | NA | NA | NA | NA | NA |
| Mohr 2021 | Switzerland | 98 | No study name provided | NA | NA | HPV; diagnosis | Prevalence | 10.20% | NA | NA | NA | NA | NA | NA | NA |
| MuesesMar√≠n 2020 | Colombia | 59 | No study name provided | NA | NA | STI ever; self-report diagnosis | Prevalence | NA | NA | NA | 22.00% | NA | NA | NA | NA |
| Niforatos 2020 | United States | 10160 | Explorys, Inc Database | NA | NA | Syphilis; diagnosis | Prevalence | 110.00% | NA | NA | NA | NA | NA | NA | NA |
| Niforatos 2020 | United States | 10160 | Explorys, Inc Database | NA | NA | HPV; diagnosis | Prevalence | 23.00% | NA | NA | NA | NA | NA | NA | NA |
| Passaro 2020a. | Peru | 120 | No study name provided | 18+ | NA | Rectal gonorrhea/chlamydia; lab test | Prevalence | NA | NA | NA | 30.00% | NA | NA | NA | NA |
| Passaro 2020a. | Peru | 120 | No study name provided | 18+ | NA | Syphilis; lab test | Prevalence | NA | NA | NA | 5.80% | NA | NA | NA | NA |
| Passaro 2020b. | Peru | 137 | No study name provided | 18+ | People engaged in sex with a male or transfeminine partner in P12M | Bacterial STI (syphilis, gonorrhea, or chlamydia).; diagnosis | Prevalence | NA | NA | NA | 30.70% | NA | NA | NA | NA |
| Paulino-Ramirez 2021 | Dominican Republic | 255 | No study name provided | NA | NA | Syphilis; serology | Prevalence | NA | NA | NA | 47.50% | NA | NA | NA | NA |
| Plummer 2021 | United States | 71 | No study name provided | 18+ | NA | High-risk HPV; diagnosis | Prevalence | NA | NA | 18.50% | NA | NA | NA | NA | NA |
| Poteat 2020a. | South Africa | 213 | No study name provided | 18+ | NA | STI ever; self-report | Prevalence | NA | NA | NA | 38.00% | NA | NA | NA | NA |
| Ramadhani 2020 | Nigeria | 311 | TRUST/RV368 cohort | Abuja: 16+; Lagos: 18+ | NA | Gonorrhoea; diagnosis | Incidence | NA | NA | NA | 33.4 per 100 PY | NA | NA | NA | 28.9 per 100 PY |
| Ramadhani 2020 | Nigeria | 311 | TRUST/RV368 cohort | Abuja: 16+; Lagos: 18+ | NA | Chlamydia; diagnosis | Incidence | NA | NA | NA | 33.9 per 100 PY | NA | NA | NA | 26.8 per 100 PY |
| Rutherford 2021 | Canada | 340 | Sex Now Survey | 15+ | NA | Chlamydia, gonorrhea, or syphilis P12M; self-report | Prevalence | NA | NA | 9.80% | NA | NA | NA | NA | 12.00% |
| Santos 2021 | Brazil | 415 | No study name provided | NA | NA | Syphilis; self-report | Prevalence | 39.40% | 67.50% | NA | 39.30% | NA | NA | NA | NA |
| Secco 2020 | United States | 138 | DC Cohort Study | NA | PLHIV | STI; diagnosis | Incidence | NA | NA | NA | 4.9 per 100 PY | NA | NA | NA | NA |
| She 2021b. | China | 204 | No study name provided | 18+ | TW SW | STI ever; self-report | Prevalence | NA | NA | NA | 26.50% | NA | NA | NA | NA |
| Smeaton 2020 | United States; Canada; Spain; Brazil; Peru; Haiti; Thailand; India; South Africa; Uganda; Zimbabwe; Botswana | 129 | REPRIEVE | 40-75 | PLHIV | Chronic active Hepatitis B; self-report | Prevalence | NA | 3.30% | 0.00% | NA | NA | NA | NA | NA |
| Sok 2020 | Cambodia | 1375 | No study name provided | 18+ | NA | STI symptoms; self-report | Prevalence | NA | NA | NA | 14.00% | NA | NA | NA | NA |
| Storm 2020 | Nepal | 173 | No study name provided | 16+ | NA | Active syphilis; tested in study | Prevalence | NA | NA | NA | 11.00% | NA | NA | NA | NA |
| Swartz 2021 | United States | 223 | No study name provided | 18+ | TWLHIV | STI; self-report of physician diagnosis | Prevalence | NA | NA | NA | 73.10% | NA | NA | NA | NA |
| TwahirwaRwema 2020 | Rwanda | 106 | No study name provided | 18+ | NA | Gonorrhoea; tested in study | Prevalence | NA | NA | NA | 11.30% | NA | NA | NA | NA |
| TwahirwaRwema 2020 | Rwanda | 106 | No study name provided | 18+ | NA | Chlamydia; tested in study | Prevalence | NA | NA | NA | 11.30% | NA | NA | NA | NA |
| TwahirwaRwema 2020 | Rwanda | 106 | No study name provided | 18+ | NA | Syphilis; tested in study | Prevalence | NA | NA | NA | 3.80% | NA | NA | NA | NA |
| Uaamnuichai 2021 | Thailand | 62 | No study name provided | NA | TW with neovagina | High-risk HPV; tested in study | Prevalence | NA | NA | NA | Neovaginal: 20.0%; anal: 16.7% | NA | NA | NA | NA |
| Wilson 2021a. | Brazil | 345 | Transcender | 18+ | NA | Active syphilis; tested in study | Prevalence | NA | NA | NA | Youth: 29.5%; adults: 34.0% | NA | NA | NA | NA |
| Wilson 2021a. | Brazil | 345 | Transcender | 18+ | NA | Rectal chlamydia; tested in study | Prevalence | NA | NA | NA | Youth: 15.9%; adults: 13.4% | NA | NA | NA | NA |
| Wilson 2021a. | Brazil | 345 | Transcender | 18+ | NA | Rectal gonorrhea; tested in study | Prevalence | NA | NA | NA | Youth: 15.7%; adults: 4.6% | NA | NA | NA | NA |
| Yan 2021 | China | 222 | No study name provided | 18+ | NA | STI P12M; self-report | Prevalence | NA | NA | NA | 3.20% | NA | NA | NA | NA |
| Substance use or mental health | | | | | | | | | | | | | | | |
| Alcohol use disorder | | | | | | | | | | | | | | | |
| Alvarado 2020 | Colombia | 59 | No study name provided | Adult | NA | Frequent alcohol use: 5+ drinks in one occasion more than once a month; self-report | Prevalence | NA | NA | NA | 67.80% | NA | NA | NA | NA |
| Antwi-Amoabeng 2020 | United States | 16555 | National Inpatient Sample | 18+ | Patients who underwent gender-affirming surgeries | Chronic alcohol use; diagnosis | Prevalence | 11.10% | NA | NA | NA | NA | NA | NA | NA |
| Aristegui 2021 | Argentina | 61 | TransViiV | 18+ | Newly diagnosed TW living with HIV initiating ART | Above cutoff for hazardous drinking; AUDIT | Prevalence | NA | NA | NA | 52.50% | NA | NA | NA | NA |
| Barger 2021 | United States | 342 | Population Research in Identity and Disparities for Equality (PRIDE) | 18+ | NA | Binge alcohol use P12M; self-report | Prevalence | NA | 33.30% | 44.70% | NA | NA | NA | NA | Gender non-binary: 7.0%; genderqueer: 44.7% |
| Batchelder 2021 | United States | 257 | No study name provided | 18+ | NA | Likely alcohol use disorder per AUDIT; AUDIT | Prevalence | NA | NA | NA | 13.40% | 7.80% | NA | NA | NA |
| Cheung 2020 | Australia | 895 | No study name provided | NA | NA | Hazardous alcohol intake; diagnosis | Prevalence | Adult binary: 9.3% | NA | NA | NA | NA | NA | NA | Adult non-binary: 10.1% |
| Denby 2021 | United States | 427 | No study name provided | 18+ | Patients seeking GAHT | Alcohol dependence; diagnosis | Prevalence | 3.50% | NA | NA | NA | NA | NA | NA | NA |
| Dinger 2020 | United States | 2487 | National College Health Assessment | 18-25 | College students | Binge drinking P2W; self-report | Prevalence | Transgender: 23.8% | NA | NA | NA | NA | NA | NA | Non-binary, non-trans: 21.4% |
| Eastwood 2021 | United States | 102 | Enhancing engagement and retention in quality HIV Care for transgender women of color initiative | 18-24 | HIV+ TW of color | Significant alcohol use problems; CAGE | Prevalence | NA | NA | NA | 13.70% | NA | NA | NA | NA |
| Felner 2021 | United States | 144 | Growing Up Today Study | NA | NA | 12-month alcohol abuse/dependence; adapted from National Survey on Drug Use and Health | Prevalence | 22.20% | 28.30% | 17.90% | NA | NA | NA | NA | NA |
| Ferlatte 2020a. | Canada | 209 | Sex Now Survey (2015) | NA | TMSM | Frequent binge drinking; self-report | Prevalence | NA | NA | NA | NA | 4.30% | NA | NA | NA |
| Fish 2021a. | United States | 7045 | California Healthy Kids Survey (CHKS) | 12+ | NA | Alcohol use P1M; self-report | Prevalence | 31.20% | NA | NA | NA | NA | NA | NA | NA |
| Fish 2021a. | United States | 7045 | California Healthy Kids Survey (CHKS) | 12+ | NA | Heavy episodic binge drinking P1M; self-report | Prevalence | 22.80% | NA | NA | NA | NA | NA | NA | NA |
| Fish 2021b. | United States | 7045 | California Healthy Kids Survey | 12+ | NA | Heavy episodic binge drinking P1M: Self-reporting having 5+ drinks of alcohol in a row (within a couple of hours) P1M.; self-report | Prevalence | 22.80% | NA | NA | NA | NA | NA | NA | NA |
| Flentje 2020 | United States | 1892 | The PRIDE Study | 18+ | NA | Harmful alcohol use P12M; AUDIT | Prevalence | NA | 12.90% | 10.50% | NA | NA | NA | NA | 13.60% |
| Flentje 2020 | United States | 1892 | The PRIDE Study | 18+ | NA | Alcohol dependence symptoms and consequences; items from AUDIT | Prevalence | NA | 27.60% | 29.50% | NA | NA | NA | NA | 34.00% |
| Fuxman 2021 | United States | 323 | MWAHS (MetroWest Adolescent Health Survey) | Grades 9-12 | NA | Binge drinking P1M; self-report | Prevalence | 25.00% | NA | NA | NA | NA | NA | NA | NA |
| Gilbert 2020 | United States | 137 | No study name provided | 18+ | NA | Heavy drinking; NA | Prevalence | 18.70% | NA | NA | NA | NA | NA | NA | NA |
| Halli 2021 | India | 282 | No study name provided | 18+ | NA | Alcohol consumption daily; self-report | Prevalence | 23.00% | NA | NA | NA | NA | NA | NA | NA |
| Harper 2021 | Kenya | 62 | No study name provided | 18+ | NA | Alcohol use daily; self-report | Prevalence | 5.50% | NA | NA | NA | NA | NA | NA | NA |
| Harper 2021 | Kenya | 62 | No study name provided | 18+ | NA | Home brew use daily; self-report | Prevalence | 1.90% | NA | NA | NA | NA | NA | NA | NA |
| Hickson 2020 | Europe | 1047 | The European MSM Internet Survey 2017 | Legal age of consent | NA | Alcohol dependency; CAGE | Prevalence | NA | NA | NA | NA | AMAB trans men: 20.7%  AFAB trans men: 26.3%  AFAB men: 22.3% | NA | NA | NA |
| Hillman 2021 | United States | 3462 | USTS | 50+ | NA | Binge drinking, P1M | Prevalence | 15.90% | NA | NA | NA | NA | NA | NA | NA |
| Hisle-Gorman 2021 | United States | 3754 | No study name provided | <18 at first contact | Youth in the military healthcare system | Alcohol use disorder; diagnosis | Prevalence | 1.50% | NA | NA | NA | NA | NA | NA | NA |
| Holloway 2021 | United States | 58 | No study name provided | 18+ | Military servicemembers | Problematic alcohol use; AUDIT-C | Prevalence | 48.30% | NA | NA | NA | NA | NA | NA | NA |
| Horwitz 2020 | United States | NA | eBridge Study | 18+ | College students | Heavy alcohol use; AUDIT | Prevalence | NA | NA | NA | 12.10% | 11.30% | 16.70% | 11.30% | NA |
| Hughto 2021c. | United States | 15637 | OLDW (OptimLabs Data Warehouse) | 18+ | NA | Alcohol and drug use disorder; diagnosis | Prevalence | 5.70% | NA | NA | NA | NA | NA | NA | NA |
| Hughto 2021c. | United States | 15637 | OLDW (OptimLabs Data Warehouse) | 18+ | NA | AUD; diagnosis | Prevalence | 2.60% | NA | NA | NA | NA | NA | NA | NA |
| Katz-Wise 2021 | United States | 33 | Trans Teen and Family Narratives (TTFN) Project | 13-17 | NA | Alcohol use ever; self-report | Prevalence | 26.70% | NA | NA | NA | NA | NA | NA | NA |
| Kcomt 2020b. | United States | 27715 | USTS | 18+ | NA | Frequent binge drinking P1M; self-report | Prevalence | NA | NA | NA | 57.60% | 63.00% | 66.60% | 60.70% | Crossdressers: 67.7% |
| Kota 2020 | United States | 92 | No study name provided | 18-65 | NA | Excessive drinking; diagnosis | Prevalence | NA | NA | NA | 21.00% | NA | NA | NA | NA |
| Lee 2020a. | South Korea | 255 | Rainbow Connection Project II - Korean Transgender Adults Health Study | 19+ | Diagnosed with gender identity disorder, ever used hormone therapy, or received any kind of gender affirmation surgery. | Hazardous drinking; self-report | Prevalence | 13.60% | 14.70% | 12.20% | NA | NA | NA | NA | NA |
| Lindley 2021 | United States | 297 | No study name provided | 18+ | NA | Hazardous drinking; AUDIT | Prevalence | 1660.00% | 14.20% | 17.40% | NA | NA | NA | NA | 20.30% |
| Logie 2020b. | Jamaica | 101 | No study name provided | NA | NA | Binge drinking; self-report | Prevalence | NA | NA | NA | 28.70% | NA | NA | NA | NA |
| Long 2020 | Peru | 142 | Sabes | 18+ | NA | Alcohol dependency; AUDIT | Prevalence | NA | NA | NA | 31.00% | NA | NA | NA | NA |
| Madhavan 2020 | India | 200 | No study name provided | 18+ | NA | Alcohol dependence; AUDIT | Prevalence | Current alcohol users: hazardous use 45.0%; harmful use 21.0%; possible dependence 19.3% | NA | NA | NA | NA | NA | NA | NA |
| Majumder 2020 | India | 120 | No study name provided | 18-70 | NA | Addiction to alcohol | Prevalence | NA | 33.90% | 45.50% | NA | NA | NA | NA | NA |
| Miller 2020 | Guatemala | 142 | No study name provided | 18+ | TW SW | Binge drinking P1W; self-report | Prevalence | NA | NA | NA | 40.20% | NA | NA | NA | NA |
| Mohr 2021 | Switzerland | 98 | No study name provided | NA | NA | Alcohol abuse; diagnosis | Prevalence | 4.10% | NA | NA | 9.40% | 2.20% | NA | NA | NA |
| Passaro 2020a. | Peru | 120 | No study name provided | 18+ | NA | Alcohol use disorder; AUDIT | Prevalence | NA | NA | NA | 63.30% | NA | NA | NA | NA |
| Passaro 2020a. | Peru | 120 | No study name provided | 18+ | NA | Alcohol use disorder; AUDIT | Prevalence | NA | NA | NA | 63.30% | NA | NA | NA | NA |
| Passaro 2020b. | Peru | 137 | No study name provided | 18+ | People engaged in sex with a male or transfeminine partner in P12M | Hazardous drinking; AUDIT-10 | Prevalence | NA | NA | NA | 72.30% | NA | NA | NA | NA |
| Passaro 2020b. | Peru | 137 | No study name provided | 18+ | People engaged in sex with a male or transfeminine partner in P12M | Binge drinking: AUDIT-3 | Prevalence | NA | NA | NA | 80.70% | NA | NA | NA | NA |
| Pharr 2021 | United States | 2827 | BRFSS (Behavioral Risk Factor Surveillance System) | 18+ | NA | Heavy alcohol consumption; self-report | Prevalence | 6.70% | NA | NA | NA | NA | NA | NA | NA |
| Poteat 2020a. | South Africa | 213 | No study name provided | 18+ | NA | Alcohol use disorder P12M; AUDIT-C | Prevalence | NA | NA | NA | 83.00% | NA | NA | NA | NA |
| Poteat 2020c. | United States | 1020 | LITE (American Cohort to Study HIV Acquisition Among Transgender Women in High Risk Areas) | 18+ | NA | AUD; self-report | Prevalence | NA | NA | NA | 29.00% | NA | NA | NA | NA |
| Progovac 2021 | United States | 916 | No study name provided | Dec-80 | NA | AUD; diagnosis | Prevalence | 6.70% | NA | NA | NA | NA | NA | NA | NA |
| Radusky 2020 | Argentina | 61 | No study name provided | 18+ | People initiating HIV care | Hazardous drinking; AUDIT | Prevalence | NA | NA | NA | 52.50% | NA | NA | NA | NA |
| Radusky 2021b. | Argentina | 41 | COPA2 (Conexiones y Opciones Positivas en la Argentina 2) | 18+ | Individuals disengaged from HIV care | Hazardous alcohol use; AUDIT | Prevalence | NA | NA | NA | 46.30% | NA | NA | NA | NA |
| Restar 2020a. | United States | 297 | Project LifeSkills | 16-29 | NA | AUD recent; self-report | Prevalence | NA | NA | NA | 10.80% | NA | NA | NA | NA |
| Santos 2021 | Brazil | 415 | No study name provided | NA | NA | Alcohol use daily P6M; self-report | Prevalence | 13.20% | 16.50% | NA | 11.00% | NA | NA | NA | NA |
| Sartaj 2021 | India | 50 | No study name provided | 18-60 | Hijra | Alcohol use; WHO-ASSIST | Prevalence | NA | Moderate: 16.0%; High: 14.0% | NA | NA | NA | NA | NA | NA |
| Schlissel 2021 | United States | 1567 | Survey of Today's Adolescent Relationships and Transitions | 13-24 | NA | Binge drinking P1M; self-report | Prevalence | 58.50% | NA | NA | 84.70% | 26.80% | NA | NA | 30.80% |
| Stanton 2021 | United States | 3179 | No study name provided | 18+ | NA | AUD; AUDIT-C | Prevalence | NA | NA | NA | 8.70% | 7.20% | 11.40% | 11.00% | NA |
| Suarez 2021 | United States | 131 | No study name provided | 21-64 | NA | Binge drinking at least monthly; self-report | Prevalence | NA | NA | 19.2%-22.0% (across number of adverse childhood experiences) | NA | NA | NA | NA | NA |
| Turban 2020 | United States | 3494 | USTS | 18-36 | TGD individuals who wanted puberty blockers | Binge drinking P1M; self-report | Prevalence | Received pubertal suppression: 29.2%; did not receive pubertal suppression: 24.3% | NA | NA | NA | NA | NA | NA | NA |
| Turban 2021 | United States | 9711 | USTS | 18+ | TGD and gender diverse people who had undergone social transition and realized they were TGD during childhood | Binge drinking P1M; self-report | Prevalence | 26.00% | NA | NA | NA | NA | NA | NA | NA |
| vanHeesewijk 2021 | Netherlands | 37 | No study name provided | 55-70 | NA | Heavy alcohol consumption; self-report | Prevalence | NA | NA | NA | 21.60% | NA | NA | NA | NA |
| Vi 2020 | Vietnam | 456 | No study name provided | 18+ | NA | Alcohol consumption ‚â•2-3 times per week P12M; self-report | Prevalence | NA | NA | NA | 17.60% | NA | NA | NA | NA |
| Vi 2020 | Vietnam | 456 | No study name provided | 18+ | NA | Drinks of alcohol per week; self-report | Prevalence | NA | NA | NA | 3-4: 29.7%; 5-6: 19.5%; 7+: 9.3% | NA | NA | NA | NA |
| Watson 2020 | United States | 3868 | LGBTQ National Teen Survey | 13-17 | NA | Alcohol use ever and recent; self-report | Prevalence | NA | Alcohol use ever: 54.8%; alcohol use recent: 27.0% | Alcohol use ever: 59.2%; alcohol use recent: 27.5% | NA | NA | Alcohol use ever: 52.8%; alcohol use recent: 22.3% | Alcohol use ever: 54.5%; alcohol use recent: 24.2% | NA |
| Watson 2020 | United States | 3868 | LGBTQ National Teen Survey | 13-17 | NA | Binge drinking P1M; self-report | Prevalence | NA | 12.70% | 10.20% | NA | NA | 9.40% | 7.40% | NA |
| Wichaidit 2021 | Thailand | 755 | National School Survey on Alcohol Consumption, Substance Use and Other Health-Risk Behaviors | Years 7, 9 and 11 students | NA | Drinker ever and drank P12M (among drinkers ever); self-report | Prevalence | NA | NA | NA | Ever: 33.6%; P12M: 79.1% | Ever: 51.1%; P12M: 75.1% | NA | NA | NA |
| Wilkerson 2020 | United States | 197 | Tell Us Texas | 13+ | NA | Hazardous drinking; AUDIT-C | Prevalence | 16.70% | NA | NA | NA | NA | NA | NA | 16.70% |
| Williams 2021b. | United States | 8619 | No study name provided | 18+ | Veterans in VA care | Unhealthy or high-risk alcohol use; AUDIT-C | Prevalence | Unhealthy: 6.6%; high-risk: 2.8% | NA | NA | NA | NA | NA | NA | NA |
| Williams 2021b. | United States | 8619 | No study name provided | 18+ | Veterans in VA care | AUD P12M; diagnosis | Prevalence | 8.60% | NA | NA | NA | NA | NA | NA | NA |
| Wilson 2021c. | Nepal | 200 | Sweekar | 18+ | NA | Binge drinking P12M; self-report | Prevalence | 79.00% | NA | NA | NA | NA | NA | NA | NA |
| Yi 2020 | Cambodia | 1039 | No study name provided | 18+ | NA | Binge drinking; self-report | Prevalence | NA | NA | NA | 43.30% | NA | NA | NA | NA |
| Zea 2021 | Colombia | 58 | No study name provided | 18-49 | NA | Binge drinking P3M; self-report | Prevalence | NA | NA | NA | 85.30% | NA | NA | NA | NA |
| Anxiety | | | | | | | | | | | | | | | |
| Aldridge 2020 | United Kingdom | 178 | No study name provided | 17+ | NA | Anxiety; HADS-A: Hospital Anxiety and Depression Scale | Prevalence | 51.10% | NA | NA | NA | NA | NA | NA | NA |
| Arikawa 2021 | United States | 117 | No study name provided | 18-35 | NA | High anxiety; Beck Anxiety Inventory (BAI) | Prevalence | NA | NA | NA | NA | 16.20% | NA | NA | 22.20% |
| Banks 2021 | United States | 470 | No study name provided | 17+ | People using GAHT | Anxiety; diagnosis | Prevalence | 13.80% | 11.70% | 16.10% | NA | NA | NA | NA | NA |
| Batchelder 2021 | United States | 257 | No study name provided | 18+ | NA | Positive screen for anxiety; GAD-7 | Prevalence | NA | NA | NA | 34.70% | 33.40% | NA | NA | NA |
| Bohlmann 2021 | United States | 97 | No study name provided | 18+ | NA | Anxiety; self-report | Prevalence | 49.50% | NA | NA | 29.80% | 46.80% | NA | NA | Non-binary: 8.3%; GNC: 10.6%; bigender/two-spirit: 4.3% |
| Boskey 2020 | United States | 158 | No study name provided | 14-35 | AFAB trans people with a diagnosis of GD, seeking top surgery | Anxiety ever; self-report | Prevalence | 66.00% | NA | NA | NA | NA | NA | NA | NA |
| Branstrom 2020 | Sweden | NA | No study name provided | NA | NA | Outpatient visit for any anxiety disorder in 2015; diagnosis | Prevalence | 7.40% | NA | NA | NA | NA | NA | NA | NA |
| Bretherton 2021 | Australia | 928 | No study name provided | 18+ | NA | Anxiety ever; self-report | Prevalence | 67.00% | NA | NA | NA | NA | NA | NA | NA |
| Bryant-Genevier 2021 | United States | 147 | No study name provided | NA | State, Tribal, local, and territorial public health workers during the COVID-19 pandemic | Anxiety P2W; GAD-2 | Prevalence | 61.10% | NA | NA | NA | NA | NA | NA | NA |
| Burns 2021 | United States | 37 | No study name provided | NA | Patients with cancer | Anxiety or mood disorders ever; diagnosis | Prevalence | 51.40% | NA | NA | NA | NA | NA | NA | NA |
| Cantu 2020 | United States | 80 | No study name provided | 18-Nov | NA | Met cutoff for anxiety at initial visit (GAD-7 >6); GAD-7 | Prevalence | 61.00% | NA | NA | NA | NA | NA | NA | NA |
| Chen 2020 | China | 250 | No study name provided | 18+ | NA | Diagnosed with anxiety; self-report of physician diagnosis | Prevalence | NA | NA | NA | 28.00% | NA | NA | NA | NA |
| Cheung 2020 | Australia | 895 | No study name provided | NA | NA | Anxiety; diagnosis | Prevalence | Adult binary: 34.6% | NA | NA | NA | NA | NA | NA | Adult non-binary: 64.6% |
| Chumakov 2021 | Russia | 588 | No study name provided | 18+ | NA | Clinical-level anxiety; Hospital Anxiety and Depression Scale (HADS) with Anxiety sub-scale | Prevalence | NA | 35.30% | 47.40% | NA | NA | NA | NA | 53.50% |
| Coakley 2021 | United States | 53 | No study name provided | 18+ | University students | Degree of anxiety symptoms; GAD-7 | Prevalence | Moderate symptoms: 34.0%; severe symptoms: 39.6% | NA | NA | NA | NA | NA | NA | NA |
| Denby 2021 | United States | 427 | No study name provided | 18+ | Patients seeking GAHT | Anxiety disorder; diagnosis | Prevalence | 31.80% | NA | NA | NA | NA | NA | NA | NA |
| Dinger 2020 | United States | 2487 | National College Health Assessment | 18-25 | College students | Diagnosed with anxiety P12M; self-report of physician diagnosis | Prevalence | Transgender: 44.7% | NA | NA | NA | NA | NA | NA | Non-binary, non-trans: 39.1% |
| Fontanari 2020 | Brazil | 350 | No study name provided | 16-24 | NA | Recent anxiety; Overall Anxiety Severity and Impairment Scale (OASIS) | Prevalence | 67.90% | NA | NA | NA | NA | NA | NA | NA |
| Gonzales 2020 | United States | 201 | No study name provided | 18-25 | College students | Generalized anxiety disorder; GAD-2 | Prevalence | Transgender: 76.7% | NA | NA | NA | NA | NA | NA | GNC: 70.0%; other: 79.0% |
| Halli 2021 | India | 282 | No study name provided | 18+ | NA | High anxiety; not specified | Prevalence | 45.40% | NA | NA | Hijra: 47.2% | NA | Kothi: 44.3% | NA | NA |
| Heller 2021 | United States | 659 | National College Health Assessment | 18+ | College students | Anxiety P12M; self-report | Prevalence | NA | NA | NA | 55.00% | 69.30% | NA | NA | 56.70% |
| Henry 2021 | United States | 78 | No study name provided | 18+ | NA | Clinically significant anxiety symptomology (P1W); Hopkins Symptom Checklist-25 | Prevalence | 11.50% | NA | NA | NA | NA | NA | NA | NA |
| Hisle-Gorman 2021 | United States | 3754 | No study name provided | <18 at first contact | Youth in the military healthcare system | Anxiety disorder; diagnosis | Prevalence | 50.80% | NA | NA | NA | NA | NA | NA | NA |
| Holloway 2021 | United States | 58 | No study name provided | 18+ | Military servicemembers | Anxiety symptoms; GAD-7 | Prevalence | Mild: 15.5%; Moderate: 13.8%; Severe: 3.5% | NA | NA | NA | NA | NA | NA | NA |
| Hughto 2021a. | United States | 580 | No study name provided | 18+ | NA | Anxiety symptoms P1W; Brief Symptom Inventory | Prevalence | 12.90% | NA | NA | NA | NA | NA | NA | NA |
| Hughto 2021b. | United States | 545 | No study name provided | 18+ | NA | Anxiety symptoms; Brief Symptom Inventory | Prevalence | 12.80% | NA | NA | NA | NA | NA | NA | NA |
| James 2020 | United States | 82 | Rochester Epidemiology Project | NA | Patients seeking gender-related healthcare | Anxiety; diagnosis | Prevalence | NA | 61.20% | 64.50% | NA | NA | NA | NA | NA |
| Jarrett 2021 | Multinational | 964 | COVID Disparities Working Group | 18+ | Hornet or Her app users | Anxiety symptoms; PHQ-4 | Prevalence | 45.80% | 52.70% | 51.50% | NA | NA | NA | NA | 42.70% |
| Joshi 2021 | India | 33 | No study name provided | 18+ | NA | Anxiety symptoms P2W; GAD-7 | Prevalence | NA | NA | NA | Moderate: 45.4% Severe: 39.4% | NA | NA | NA | NA |
| Kaltiala 2020 | Finland | 52 | No study name provided | 15-19 | NA | Anxiety during and before gender assessment; diagnosis | Prevalence | 48.00% | NA | NA | NA | NA | NA | NA | NA |
| Kattari 2020a. | United States | 659 | Michigan Trans Health Survey | 18+ | NA | Anxiety ever; self-report of physician diagnosis | Prevalence | 73.00% | NA | NA | NA | NA | NA | NA | NA |
| Khorashad 2021 | Iran | 205 | No study name provided | NA | NA | Anxiety disorder; diagnosis | Prevalence | Current 5.6%; Lifetime 1.0% | NA | NA | Current: 4.2%; lifetime: 0.0% | Current: 1.2%; lifetime: 1.8% | NA | NA | NA |
| Kidd 2021b. | United States | 208 | Project AFFIRM | 18+ | NA | Clinically significant anxiety symptoms during pandemic; Brief Symptom Inventory | Prevalence | 41.30% | NA | NA | NA | NA | NA | NA | NA |
| Kirby 2020 | United States | 26 | No study name provided | 18+ | College students | Anxiety; self-report | Prevalence | 85.00% | NA | NA | NA | NA | NA | NA | NA |
| Klemmer 2021 | United States | 233 | No study name provided | 18+ | NA | Anxiety ever; self-report of physician diagnosis | Prevalence | NA | NA | NA | 42.10% | NA | NA | NA | NA |
| Konrad 2020 | Germany | 585 | Disease Analyzer database (IQVIA) | M=34.8 | NA | Anxiety; diagnosis | Prevalence | 5.80% | NA | NA | NA | NA | NA | NA | NA |
| Lane 2020 | United States | 103 | No study name provided | 18+ | TM seeking gender affirming mastectomies | Positive screen for anxiety; GAD-7 | Prevalence | NA | NA | 66.30% | NA | NA | NA | NA | NA |
| Leon 2021 | United States | 185 | Gender Wellness Center Pediatric Patient Registry | 25-Jul | NA | Anxiety disorder ever; diagnosis | Prevalence | 42.20% | NA | NA | NA | NA | NA | NA | NA |
| Levit 2021 | Israel | 115 | No study name provided | NA | NA | Anxiety; diagnosis | Prevalence | NA | NA | NA | 45.50% | 23.50% | NA | NA | NA |
| Liu 2020 | China | 1304 | No study name provided | M=22 | NA | Anxiety; self-report | Prevalence | 44.90% | NA | NA | 47.00% | 42.00% | NA | NA | NA |
| McNichols 2020 | United States | 246 | No study name provided | NA | NA | Anxiety ever; self-report | Prevalence | NA | NA | NA | NA | 70.00% | NA | NA | NA |
| Parr 2020 | United States | 1523 | Healthy Minds Study | NA | College students | Anxiety; GAD-7 | Prevalence | NA | NA | NA | 55.00% | NA | NA | NA | NA |
| Polizopoulos-Wilson 2021 | United States | 62 | No study name provided | 18+ | NA | Anxiety; self-report | Prevalence | 51.00% | NA | NA | NA | NA | NA | NA | NA |
| Poteat 2021b. | United States | 153 | No study name provided | NA | Gay-straight alliance members | Anxiety P1M; Beck Anxiety Inventory | Prevalence | 54.50% | NA | NA | NA | NA | NA | NA | 56.90% |
| Progovac 2021 | United States | 916 | No study name provided | Dec-80 | NA | Anxiety disorder; diagnosis | Prevalence | 43.20% | NA | NA | NA | NA | NA | NA | NA |
| Radusky 2021b. | Argentina | 41 | COPA2 (Conexiones y Opciones Positivas en la Argentina 2) | 18+ | Individuals disengaged from HIV care | Anxiety ever; BDI-II | Prevalence | NA | NA | NA | 51.20% | NA | NA | NA | NA |
| Reisner 2020 | United States | 843 | No study name provided | 18+ | HIV-negative transmasculine people who had sex with cis men in P6M | General Anxiety P2W; GAD-7 | Prevalence | NA | NA | Moderate: 22.5%; Severe: 14.7% | NA | NA | NA | NA | NA |
| Restar 2020a. | United States | 297 | Project LifeSkills | 16-29 | NA | Generalized Anxiety Disorder recent; self-report | Prevalence | NA | NA | NA | 37.70% | NA | NA | NA | NA |
| Restar 2020b. | United States | 600 | No study name provided | 18+ | NA | Anxiety P1W; Brief Symptom Inventory | Prevalence | 12.10% | NA | NA | NA | NA | NA | NA | NA |
| Restar 2021 | Global | 849 | Global COVID-19 Disparities Survey | 18+ | NA | Anxiety P2W; PHQ-4 | Prevalence | 45.80% | 31.90% | 4.40% | NA | NA | NA | NA | 63.80% |
| Rutherford 2021 | Canada | 340 | Sex Now Survey | 15+ | NA | Anxiety; GAD-2 | Prevalence | NA | NA | 38.90% | NA | NA | NA | NA | 42.00% |
| Sartaj 2021 | India | 50 | No study name provided | 18-60 | Hijra | Anxiety disorder; self-report | Prevalence | NA | 8.00% | NA | NA | NA | NA | NA | NA |
| Schvey 2020 | United States | 195 | No study name provided | 18+ | Military servicemembers | Anxiety; DASS-21 Anxiety Sub-scale | Prevalence | 28.30% | NA | NA | NA | NA | NA | NA | NA |
| Sergi 2021 | United States | 631 | Trans* National Study | 18-75 | NA | Anxiety ever; self-report diagnosis | Prevalence | NA | NA | NA | Filler users: 46.1%; Non-filler users: 54.8% | NA | NA | NA | NA |
| She 2020 | Canada | 92 | No study name provided | NA | NA | Anxiety disorder ever; diagnosis | Prevalence | Ever: 62.0%; Current: 58.7% | NA | NA | NA | NA | NA | NA | NA |
| She 2021a. | China | 235 | No study name provided | 18+ | TW SW | Generalized Anxiety; GAD-7 | Prevalence | NA | NA | NA | Sample 1: 79.4%; Sample 2: 68.8% | NA | NA | NA | NA |
| She 2021a. | China | 235 | No study name provided | 18+ | TW SW | Social anxiety disorder; Mini-SPIN | Prevalence | NA | NA | NA | Sample 1: 26.2%; Sample 2: 24.8% | NA | NA | NA | NA |
| She 2021b. | China | 204 | No study name provided | 18+ | TW SW | Generalized Anxiety Disorder; GAD-7 | Prevalence | NA | NA | NA | 51.00% | NA | NA | NA | NA |
| Silva 2021 | Brazil | 189 | No study name provided | NA | NA | Anxiety; DASS-21 | Prevalence | Moderate: 12.4%; Severe: 7.9% | NA | NA | Moderate: 14.7%; Severe: 10.1% | Moderate: 8.8%; Severe: 4.4% | NA | NA | NA |
| Silveri 2021 | United States | 35 | No study name provided | 13-17 | Adolescents in residential psychiatric treatment | Generalized anxiety disorder; MASC | Prevalence | 62.50% | NA | NA | NA | NA | NA | NA | NA |
| Spanos 2021 | Australia | 589 | No study name provided | 21+ | Patients receiving consultations for hormone therapy | Anxiety; diagnosis | Prevalence | 35.10% | NA | NA | NA | NA | NA | NA | NA |
| Stanton 2021 | United States | 3179 | No study name provided | 18+ | NA | Anxiety; GAD-7 | Prevalence | NA | NA | NA | 35.00% | 33.60% | 38.00% | 46.10% | NA |
| Strang 2021a. | United States | 124 | No study name provided | 21-Nov | NA | Generalized anxiety disorder; Child and Adolescent Symptom Inventory-5 | NA | 29.80% | NA | NA | NA | NA | NA | NA | NA |
| Strauss 2020a. | Australia | 869 | Trans Pathways | 14-25 | NA | Anxiety ever; self-report of physician diagnosis | Prevalence | 72.20% | 67.20% | 73.90% | NA | NA | NA | NA | NA |
| Strauss 2020a. | Australia | 869 | Trans Pathways | 14-25 | NA | Severe anxiety current; GAD-7 | Prevalence | 31.60% | NA | NA | NA | NA | NA | NA | NA |
| Strauss 2020b. | Australia | 859 | Trans Pathways | 14-25 | NA | Severe anxiety current; GAD-7 | Prevalence | 31.60% | NA | NA | NA | NA | NA | NA | NA |
| Strauss 2020b. | Australia | 859 | Trans Pathways | 14-25 | NA | Anxiety disorder ever; self-reported psychiatric diagnosis | Prevalence | 72.20% | NA | NA | NA | NA | NA | NA | NA |
| Strauss 2021 | Australia | 859 | Trans Pathways | 14-25 | NA | Anxiety disorder ever; self-report of physician diagnosis | Prevalence | With ASD: 93.6%; without ASD: 65.9% | NA | NA | NA | NA | NA | NA | NA |
| Strauss 2021 | Australia | 859 | Trans Pathways | 14-25 | NA | Anxiety; GAD-7 | Prevalence | With ASD: 37.7%; without ASD: 29.4% | NA | NA | NA | NA | NA | NA | NA |
| Tan 2020 | Aotearoa/New Zealand | 1178 | Counting Ourselves: the Aotearoa New Zealand Trans and Non-Binary Health Survey | 14+ | NA | Anxiety; self-report of physician diagnosis | Prevalence | 55.20% | NA | NA | NA | NA | NA | NA | NA |
| Tan 2021a. | Aotearoa/New Zealand | 49 | Counting Ourselves: Aotearoa New Zealand Trans and Non-Binary Health Survey | 14+ | Asian TGD individuals | Anxiety disorder; self-report of physician diagnosis | Prevalence | 41.20% | NA | NA | NA | NA | NA | NA | NA |
| Taylor 2020 | Australia | 474 | Who I Am Study | 18+ | Bisexual Australians | Anxiety disorder; self-report of physician diagnosis | Prevalence | 63.90% | NA | NA | NA | NA | NA | NA | NA |
| Tebbe 2021 | United States | 301 | No study name provided | 18+ | NA | Moderate or higher anxiety P1W; Burns Anxiety Inventory | Prevalence | 71.40% | NA | NA | NA | NA | NA | NA | NA |
| To 2020 | United States | 620 | Study of Transition, Outcomes, and Gender (STRONG) | 18+ | NA | Anxiety symptoms; Beck Anxiety Inventory | Prevalence | 16.30% | NA | NA | NA | NA | NA | NA | NA |
| Valente 2020 | United States | 330 | Project AFFIRM | 16+ | NA | Anxiety symptoms P1W; Brief Symptom Inventory-18 | Prevalence | 39.70% | 39.30% | 40.40% | NA | NA | NA | NA | NA |
| vanHeesewijk 2021 | Netherlands | 37 | No study name provided | 55-70 | NA | Anxiety symptoms; Hopkins Symptom Checklist-25 | Prevalence | NA | NA | NA | 11.80% | NA | NA | NA | NA |
| Wathelet 2020 | France | 784 | COSAMe | NA | University students during COVID-19 pandemic | Anxiety severity; Stait-Trait Anxiety Inventory for Adults | Prevalence | NA | NA | NA | NA | NA | NA | NA | Moderate: 35.5%; high: 50.0% |
| Zwickl 2021b. | Australia | 928 | No study name provided | 18+ | NA | Anxiety; self-report | Prevalence | 79.00% | NA | NA | NA | NA | NA | NA | NA |
| Depression | | | | | | | | | | | | | | | |
| Achille 2020 | United States | 50 | No study name provided | 25-Sep | NA | Depressed P12M; CESD-R | Prevalence | 64.00% | 70.60% | 60.60% | NA | NA | NA | NA | NA |
| Aldridge 2020 | United Kingdom | 178 | No study name provided | 17+ | NA | Depression; HADS-D: Hospital Anxiety and Depression Scale | Prevalence | 47.80% | NA | NA | NA | NA | NA | NA | NA |
| Antwi-Amoabeng 2020 | United States | 16555 | National Inpatient Sample | 18+ | Patients who underwent gender-affirming surgeries | Depression; diagnosis | Prevalence | 14.60% | NA | NA | NA | NA | NA | NA | NA |
| Arikawa 2021 | United States | 117 | No study name provided | 18-35 | NA | High levels of depressive symptoms; CES-D | Prevalence | NA | NA | NA | NA | 91.40% | NA | NA | 95.50% |
| Aristegui 2021 | Argentina | 61 | TransViiV | 18+ | Newly diagnosed TW living with HIV initiating ART | Depressive symptoms P1W (CES-D >16); CES-D | Prevalence | NA | NA | NA | 50.80% | NA | NA | NA | NA |
| Atteberry-Ash 2021 | United States | 396 | 2015 Healthy Kids Colorado Survey (HKCS) | 15-18 | NA | During the P12M, did you ever feel so sad or hopeless almost every day for two weeks or more in a row that you stopped doing some usual activities?; self-report | Prevalence | 54.80% | 17.10% | 22.60% | NA | NA | NA | NA | Trans other: 22.6%; don't know: 37.8% |
| Banks 2021 | United States | 470 | No study name provided | 17+ | People using GAHT | Depression; diagnosis | Prevalence | 18.50% | 17.40% | 19.70% | NA | NA | NA | NA | NA |
| Batchelder 2021 | United States | 257 | No study name provided | 18+ | NA | Positive screen on PHQ-9; PHQ-9 | Prevalence | NA | NA | NA | 34.10% | 28.40% | NA | NA | NA |
| Biedermann 2021 | Germany | 187 | No study name provided | 18+ | NA | Depressive symptoms P2W; BDI-II, German Version | Prevalence | NA | NA | NA | Mild: 11.8%; moderate: 8.5%; severe: 7.5% | Mild: 16.3%; moderate: 6.5%; severe: 2.2% | NA | NA | NA |
| Blosnich 2021 | United States | 8981 | No study name provided | NA | VA patients | Depression ever; diagnosis | Prevalence | 76.90% | NA | NA | NA | NA | NA | NA | NA |
| Bohlmann 2021 | United States | 97 | No study name provided | 18+ | NA | Depression; self-report | Prevalence | 17.90% | NA | NA | 11.80% | 29.40% | NA | NA | Non-binary: 17.6%; GNC: 29.4%; bigender/two-spirit: 11.8% |
| Boskey 2020 | United States | 158 | No study name provided | 14-35 | AFAB trans people with a diagnosis of GD, seeking top surgery | Depression ever; self-report | Prevalence | 61.00% | NA | NA | NA | NA | NA | NA | NA |
| Bretherton 2021 | Australia | 928 | No study name provided | 18+ | NA | Depression ever; self-report | Prevalence | 73.00% | NA | NA | NA | NA | NA | NA | NA |
| Brown 2020 | United States | 88 | BRFSS (Behavioral Risk Factor Surveillance System) | 45+ | NA | Diagnosed with depression ever; self-report of physician diagnosis | Prevalence | 33.20% | NA | NA | NA | NA | NA | NA | NA |
| Bryant-Genevier 2021 | United States | 147 | No study name provided | NA | State, Tribal, local, and territorial public health workers during the COVID-19 pandemic | Depression P2W; PHQ-9 | Prevalence | 62.40% | NA | NA | NA | NA | NA | NA | NA |
| Cantu 2020 | United States | 80 | No study name provided | 18-Nov | NA | Meeting cutoff for depression at initial visit (PHQ-9 >11); PHQ-9 | Prevalence | 46.00% | NA | NA | NA | NA | NA | NA | NA |
| Chen 2020 | China | 250 | No study name provided | 18+ | NA | Diagnosed with depression; self-report of physician diagnosis | Prevalence | NA | NA | NA | 32.80% | NA | NA | NA | NA |
| Cheung 2020 | Australia | 895 | No study name provided | NA | NA | Depression; diagnosis | Prevalence | Adult binary: 52.3% | NA | NA | NA | NA | NA | NA | Adult non-binary: 70.7% |
| Chumakov 2021 | Russia | 588 | No study name provided | 18+ | NA | Clinical-level depression; Hospital Anxiety and Depression Scale (HADS) with Depression sub-scale | Prevalence | NA | 21.30% | 24.20% | NA | NA | NA | NA | 30.20% |
| Denby 2021 | United States | 427 | No study name provided | 18+ | Patients seeking GAHT | Depression disorder; diagnosis | Prevalence | 40.90% | NA | NA | NA | NA | NA | NA | NA |
| Dinger 2020 | United States | 2487 | National College Health Assessment | 18-25 | College students | Diagnosed with depression P12M; self-report of physician diagnosis | Prevalence | Transgender: 43.8% | NA | NA | NA | NA | NA | NA | Non-binary, non-trans: 35.7% |
| DuBois 2020 | United States | 786 | BRFSS (Behavioral Risk Factor Surveillance System) | 18+ | NA | Depressive disorder ever; self-report | Prevalence | NA | NA | NA | 25.60% | 39.30% | NA | NA | 44.40% |
| Eastwood 2021 | United States | 102 | Enhancing engagement and retention in quality HIV Care for transgender women of color initiative | 18-24 | HIV+ TW of color | Significant depression; CES-D-10 | Prevalence | NA | NA | NA | 51.30% | NA | NA | NA | NA |
| Felner 2021 | United States | 144 | Growing Up Today Study | NA | NA | Depression; CES-D | Prevalence | 44.40% | 41.70% | 46.40% | NA | NA | NA | NA | NA |
| Ferlatte 2020b. | Canada | 998 | No study name provided | 18+ | SGM | Depression symptoms P2W; PHQ-9 | Prevalence | NA | NA | NA | 40.20% | 49.80% | NA | NA | 55.30% |
| Gava 2021 | Italy | 108 | No study name provided | 18+ | NA | Depressive symptoms; BDI | Prevalence | Mild to moderate: 20.4%; moderate to severe: 8.3%; severe: 3.7% | NA | NA | NA | NA | NA | NA | NA |
| Gonzales 2020 | United States | 201 | No study name provided | 18-25 | College students | Major depression; PHQ-2 | Prevalence | Transgender: 74.4% | NA | NA | NA | NA | NA | NA | GNC: 65.0%; other: 73.7% |
| Guzman-Gonzalez 2020 | Chile | 377 | No study name provided | 18+ | NA | Diagnosed with depression ever; self-report of physician diagnosis | Prevalence | 40.00% | 30.50% | 58.30% | NA | NA | NA | NA | 69.60% |
| Halli 2021 | India | 282 | No study name provided | 18+ | NA | High depression; not specified | Prevalence | 32.50% | NA | NA | 30.80% | NA | 33.50% | NA | NA |
| Harper 2021 | Kenya | 62 | No study name provided | 18+ | NA | Clinically significant levels of depressive symptoms; PHQ-9 | Prevalence | 21.40% | NA | NA | NA | NA | NA | NA | NA |
| Heller 2021 | United States | 659 | National College Health Assessment | 18+ | College students | Depression P12M; self-report | Prevalence | NA | NA | NA | 50.00% | 56.00% | NA | NA | 54.10% |
| Henry 2021 | United States | 78 | No study name provided | 18+ | NA | Clinically significant depression symptomology; Hopkins Symptom Checklist-25 | Prevalence | 19.20% | NA | NA | NA | NA | NA | NA | NA |
| Hershner 2021 | United States; Canada | 4611 | American College Health Association-National College Health Assessment II | NA | College students | Depression ever; self-report of physician diagnosis | Prevalence | 43.7% (USA), 29.4%(Canada) | NA | NA | NA | NA | NA | NA | NA |
| Holloway 2021 | United States | 58 | No study name provided | 18+ | Military servicemembers | High depressive symptomatology; PHQ-8 | Prevalence | 37.90% | NA | NA | NA | NA | NA | NA | NA |
| Horwitz 2020 | United States | NA | eBridge Study | 18+ | College students | Positive depression screen; PHQ-2 | Prevalence | NA | NA | NA | 39.40% | 29.90% | 45.10% | 40.90% | NA |
| Hotton 2020 | United States | 69 | POSSE Project | 18+ | Black SGM in House and Ball Community | Depressive symptoms; CES-D | Prevalence | NA | NA | NA | 18.40% | NA | NA | NA | NA |
| Hughto 2021a. | United States | 580 | No study name provided | 18+ | NA | Depression symptoms P1W; Brief Symptom Inventory | Prevalence | 15.60% | NA | NA | NA | NA | NA | NA | NA |
| Hughto 2021b. | United States | 545 | No study name provided | 18+ | NA | Depressive symptoms; Brief Symptom Inventory | Prevalence | 15.80% | NA | NA | NA | NA | NA | NA | NA |
| James 2020 | United States | 82 | Rochester Epidemiology Project | NA | Patients seeking gender-related healthcare | Depression; diagnosis | Prevalence | NA | 77.60% | 77.40% | NA | NA | NA | NA | NA |
| Jarrett 2021 | Multinational | 964 | COVID Disparities Working Group | 18+ | Hornet or Her app users | Depression symptoms; PHQ-4 | Prevalence | 50.40% | 58.80% | 48.60% | NA | NA | NA | NA | 47.10% |
| Joshi 2021 | India | 33 | No study name provided | 18+ | NA | Depressive symptoms P2W; PHQ-9 | Prevalence | NA | NA | NA | Moderate: 30.3% Moderately severe: 30.3% Severe: 21.2% | NA | NA | NA | NA |
| Kaltiala 2020 | Finland | 52 | No study name provided | 15-19 | NA | Depression during and before gender identity assessment; diagnosis | Prevalence | 54.00% | NA | NA | NA | NA | NA | NA | NA |
| Kattari 2020a. | United States | 659 | Michigan Trans Health Survey | 18+ | NA | Depression ever; self-report of physician diagnosis | Prevalence | 72.20% | NA | NA | NA | NA | NA | NA | NA |
| Khorashad 2021 | Iran | 205 | No study name provided | NA | NA | Depressive disorders; diagnosis | Prevalence | Current: 11.0%; Lifetime: 34.1% | NA | NA | Current: 12.5%; lifetime: 34.7% | Current: 9.8%; lifetime: 33.6% | NA | NA | NA |
| Kidd 2021b. | United States | 208 | Project AFFIRM | 18+ | NA | Clinically significant depressive symptoms during pandemic; Brief Symptom Inventory | Prevalence | 41.30% | NA | NA | NA | NA | NA | NA | NA |
| Kirby 2020 | United States | 26 | No study name provided | 18+ | College students | Depression; self-report | Prevalence | 77.00% | NA | NA | NA | NA | NA | NA | NA |
| Klemmer 2021 | United States | 233 | No study name provided | 18+ | NA | Depression ever; self-report of physician diagnosis | Prevalence | NA | NA | NA | 57.50% | NA | NA | NA | NA |
| Kneale 2021 | United Kingdom | 73 | Queerentine | 18+ | NA | Significant depressive symptomology; CES-D-10 | Prevalence | 83.60% | NA | NA | NA | NA | NA | NA | NA |
| Konrad 2020 | Germany | 585 | Disease Analyzer database (IQVIA) | M=34.8 | NA | Depression; diagnosis | Prevalence | 20.80% | NA | NA | NA | NA | NA | NA | NA |
| Kuper 2020 | United States | 148 | No study name provided | NA | NA | Depression; Quick Inventory of Depressive Symptoms | Prevalence | Moderate: 11.0%, severe: 4.0% | NA | NA | NA | NA | NA | NA | NA |
| Lacombe-Duncan 2021 | Canada | 54 | CHIWOS (Canadian HIV Women's Sexual and Reproductive Health Cohort Study) | 18+ | TW living with HIV | Clinically significant depressive symptoms; CES-D | Prevalence | NA | NA | NA | 45.30% | NA | NA | NA | NA |
| Lane 2020 | United States | 103 | No study name provided | 18+ | TM seeking gender affirming mastectomies | Mild to severe depression; PHQ | Prevalence | NA | NA | 70.30% | NA | NA | NA | NA | NA |
| Lee 2020a. | South Korea | 255 | Rainbow Connection Project II - Korean Transgender Adults Health Study | 19+ | Diagnosed with gender identity disorder, ever used hormone therapy, or received any kind of gender affirmation surgery. | Depressive symptoms; CES-D-11 | Prevalence | 63.60% | 66.90% | 58.60% | NA | NA | NA | NA | NA |
| Lee 2020b. | South Korea | 207 | Rainbow Connection Project II - Korean Transgender Adults Health Study | 18+ | NA | Depressive symptoms P1W; CES-D | Prevalence | 60.40% | NA | NA | 66.20% | 50.70% | NA | NA | NA |
| Lee 2021 | South Korea | 557 | No study name provided | 18+ | NA | Depressive symptoms P1W; CES-D | Prevalence | 70.70% | NA | NA | NA | NA | NA | NA | NA |
| Lett 2020 | United States | 3151 | BRFSS (Behavioral Risk Factor Surveillance System) | NA | NA | Depressive disorders; self-report of physician diagnosis | Prevalence | Black: 28.5%; White: 37.0% | NA | NA | NA | NA | NA | NA | NA |
| Levit 2021 | Israel | 115 | No study name provided | NA | NA | Depression; diagnosis | Prevalence | NA | NA | NA | 72.70% | 88.20% | NA | NA | NA |
| Liu 2020 | China | 1304 | No study name provided | M=22 | NA | Depression; self-report | Prevalence | 41.10% | NA | NA | 42.80% | 38.90% | NA | NA | NA |
| Logie 2020a. | Jamaica | 97 | No study name provided | 18+ | Sexual minority trans women | Depressive symptoms P2W; self-report | Prevalence | NA | NA | NA | 86.60% | NA | NA | NA | NA |
| McNichols 2020 | United States | 246 | No study name provided | NA | NA | Depression ever; self-report | Prevalence | NA | NA | NA | NA | 73.00% | NA | NA | NA |
| Newcomb 2020 | United States | 214 | RADAR and FAB 400 | NA | NA | Depression; PROMIS Depression Short Form | Prevalence | Mild: 21.5%; moderate: 37.9%; severe: 7.5% | NA | NA | NA | NA | NA | NA | NA |
| Papadopulos 2020 | Germany | 47 | No study name provided | 18-57 | TW who underwent gender-confirming surgery | Depression pre-surgery; self-report | Prevalence | NA | NA | NA | 2.10% | NA | NA | NA | NA |
| Parr 2020 | United States | 1523 | Healthy Minds Study | NA | College students | Depression; PHQ-9 | Prevalence | NA | NA | NA | 68.00% | NA | NA | NA | NA |
| Pharr 2021 | United States | 2827 | BRFSS (Behavioral Risk Factor Surveillance System) | 18+ | NA | Depression; self-report | Prevalence | 39.40% | NA | NA | NA | NA | NA | NA | NA |
| Polizopoulos-Wilson 2021 | United States | 62 | No study name provided | 18+ | NA | Depression; self-report | Prevalence | 50.00% | NA | NA | NA | NA | NA | NA | NA |
| Poteat 2020b. | Brazil; Thailand | 37 | HPTN 063 | 18+ | PLHIV | Depression; CES-D | Prevalence | NA | NA | NA | Thailand: 41.0%; Brazil: 80.0% | NA | NA | NA | NA |
| Poteat 2021b. | United States | 153 | No study name provided | NA | Gay-straight alliance members | Depression P1W; CESD-10 | Prevalence | 81.80% | NA | NA | NA | NA | NA | NA | 91.70% |
| Price-Feeney 2020 | United States | 8367 | No study name provided | 13-24 | NA | Depression P12M; self-report | Prevalence | 82.80% | NA | NA | 76.10% | 86.50% | 72.20% | 83.40% | NA |
| Price-Feeney 2021 | United States | 7370 | No study name provided | 13-24 | NA | Depression P12M; self-report | Prevalence | 82.60% | NA | NA | NA | NA | NA | NA | NA |
| Progovac 2021 | United States | 916 | No study name provided | Dec-80 | NA | Depressive disorder; diagnosis | Prevalence | 43.70% | NA | NA | NA | NA | NA | NA | NA |
| Radusky 2020 | Argentina | 61 | No study name provided | 18+ | People initiating HIV care | Depression; CES-D | Prevalence | NA | NA | NA | 50.80% | NA | NA | NA | NA |
| Radusky 2021b. | Argentina | 41 | COPA2 (Conexiones y Opciones Positivas en la Argentina 2) | 18+ | Individuals disengaged from HIV care | Depression ever; BDI-II | Prevalence | NA | NA | NA | 51.20% | NA | NA | NA | NA |
| Restar 2020a. | United States | 297 | Project LifeSkills | 16-29 | NA | Major depressive episode recent; self-report | Prevalence | NA | NA | NA | 14.10% | NA | NA | NA | NA |
| Restar 2020b. | United States | 600 | No study name provided | 18+ | NA | Depression P1W; Brief Symptom Inventory | Prevalence | 15.10% | NA | NA | NA | NA | NA | NA | NA |
| Restar 2021 | Global | 849 | Global COVID-19 Disparities Survey | 18+ | NA | Depression P2W; PHQ-4 | Prevalence | 50.80% | 32.40% | 3.70% | NA | NA | NA | NA | 63.90% |
| Rutherford 2021 | Canada | 340 | Sex Now Survey | 15+ | NA | Depression; PHQ-2 | Prevalence | NA | NA | 30.10% | NA | NA | NA | NA | 33.30% |
| Sabino 2021 | Brazil | 106 | No study name provided | 18+ | TW PLHIV | Depression; self-report | Prevalence | NA | NA | NA | 16.00% | NA | NA | NA | NA |
| Sartaj 2021 | India | 50 | No study name provided | 18-60 | Hijra | Depressive disorder; self-report | Prevalence | NA | 8.00% | NA | NA | NA | NA | NA | NA |
| Schvey 2020 | United States | 195 | No study name provided | 18+ | Military servicemembers | Depression; DASS-21 Depression Sub-scale | Prevalence | 34.10% | NA | NA | NA | NA | NA | NA | NA |
| Segev-Becker 2020 | Israel | 106 | No study name provided | 18-Apr | NA | Depression; diagnosis | Prevalence | NA | 24.00% | 29.00% | NA | NA | NA | NA | NA |
| Sergi 2021 | United States | 631 | Trans* National Study | 18-75 | NA | Depression ever; self-report diagnosis | Prevalence | NA | NA | NA | Filler users: 47.7%, Non-filler users: 60.5% | NA | NA | NA | NA |
| Sevelius 2021 | United States | 858 | Enhancing Engagement and Retention  in Quality HIV Care for TWC | 18+ | TW PLHIV | Depression P1W; CES-D | Prevalence | NA | NA | NA | 52.30% | NA | NA | NA | NA |
| She 2021a. | China | 235 | No study name provided | 18+ | TW SW | Depression P1W; CES-D-20 (Chinese) | Prevalence | NA | NA | NA | Sample 1: 89.7%; Sample 2: 70.6% | NA | NA | NA | NA |
| Shrestha 2020 | Malaysia | 361 | No study name provided | 18+ | NA | Depression; CES-D | Prevalence | NA | NA | NA | 56.00% | NA | NA | NA | NA |
| Silva 2021 | Brazil | 189 | No study name provided | NA | NA | Depression; DASS-21 | Prevalence | Moderate: 11.9%; Severe: 1.7% | NA | NA | Moderate: 13.8%; Severe: 2.8% | Moderate: 8.8%; Severe: 0.0% | NA | NA | NA |
| Silveri 2021 | United States | 35 | No study name provided | 13-17 | Adolescents in residential psychiatric treatment | Major depressive disorder; CES-D | Prevalence | 84.40% | NA | NA | NA | NA | NA | NA | NA |
| Snooks 2021a. | United States; Australia; United Kingdom; Canada | 848 | No study name provided | 18+ | NA | Depressive symptoms; CES-D | Prevalence | 73.70% | NA | NA | NA | NA | NA | NA | NA |
| Snooks 2021b. | United States; Australia; United Kingdom; Canada | 237 | No study name provided | 18-70 | NA | Depressive symptoms; CES-D | Prevalence | 66.70% | NA | NA | NA | NA | NA | NA | NA |
| Sorbara 2020 | Canada | 184 | No study name provided | 15+ | Patients in puberty under 18 | Depressive disorders; diagnosis | Prevalence | 46.00% | NA | NA | NA | NA | NA | NA | NA |
| Spanos 2021 | Australia | 589 | No study name provided | 21+ | Patients receiving consultations for hormone therapy | Depression; diagnosis | Prevalence | 42.30% | NA | NA | NA | NA | NA | NA | NA |
| Srivastava 2021a. | India | 1366 | No study name provided | NA | NA | Depressive symptoms P1W; CES-D | Prevalence | NA | NA | NA | 38.50% | NA | NA | NA | NA |
| Srivastava 2021b. | India | 1366 | No study name provided | 18-73 | NA | Depressive symptoms P1W; CES-D | Prevalence | 38.50% | NA | NA | 38.50% | NA | NA | NA | NA |
| Stanton 2021 | United States | 3179 | No study name provided | 18+ | NA | Depression; PHQ-9 | Prevalence | NA | NA | NA | 34.60% | 27.50% | 33.70% | 36.80% | NA |
| Strang 2021a. | United States | 124 | No study name provided | 21-Nov | NA | Major depressive disorder; Child and Adolescent Symptom Inventory-5 | Prevalence | 16.10% | NA | NA | NA | NA | NA | NA | NA |
| Strauss 2020a. | Australia | 869 | Trans Pathways | 14-25 | NA | Depression ever; self-report of physician diagnosis | Prevalence | 74.60% | 72.80% | 75.30% | NA | NA | NA | NA | NA |
| Strauss 2020a. | Australia | 869 | Trans Pathways | 14-25 | NA | Severe depression current; PHQ-9 | Prevalence | 54.80% | NA | NA | NA | NA | NA | NA | NA |
| Strauss 2020b. | Australia | 859 | Trans Pathways | 14-25 | NA | Moderately severe to severe depression current; Patient Health Questionnaire for Adolescents | Prevalence | 54.80% | NA | NA | NA | NA | NA | NA | NA |
| Strauss 2020b. | Australia | 859 | Trans Pathways | 14-25 | NA | Depression ever; self-reported psychiatric diagnosis | Prevalence | 74.60% | NA | NA | NA | NA | NA | NA | NA |
| Strauss 2021 | Australia | 859 | Trans Pathways | 14-25 | NA | Depression ever; self-report of physician diagnosis | Prevalence | With ASD: 91.3%; without ASD: 69.8% | NA | NA | NA | NA | NA | NA | NA |
| Strauss 2021 | Australia | 859 | Trans Pathways | 14-25 | NA | Depression; PHQ-9 | Prevalence | With ASD: 68.9%; without ASD: 50.8% | NA | NA | NA | NA | NA | NA | NA |
| Suarez 2021 | United States | 131 | No study name provided | 21-64 | NA | Depression P1W; CES-D | Prevalence | NA | NA | 25.0%-61.4% (across number of adverse childhood experiences) | NA | NA | NA | NA | NA |
| Tan 2020 | Aotearoa/New Zealand | 1178 | Counting Ourselves: the Aotearoa New Zealand Trans and Non-Binary Health Survey | 14+ | NA | Depression; self-report of physician diagnosis | Prevalence | 65.70% | NA | NA | NA | NA | NA | NA | NA |
| Tan 2021a. | Aotearoa/New Zealand | 49 | Counting Ourselves: Aotearoa New Zealand Trans and Non-Binary Health Survey | 14+ | Asian TGD individuals | Depression; self-report of physician diagnosis | Prevalence | 68.60% | NA | NA | NA | NA | NA | NA | NA |
| Tantirattanakulchai 2021 | Thailand | 280 | No study name provided | 18+ | NA | Depression P1W; CES-D | Prevalence | NA | 58.20% | NA | NA | NA | NA | NA | NA |
| Taylor 2020 | Australia | 474 | Who I Am Study | 18+ | Bisexual Australians | Depression; self-report of physician diagnosis | Prevalence | 70.20% | NA | NA | NA | NA | NA | NA | NA |
| Tebbe 2021 | United States | 301 | No study name provided | 18+ | NA | Depressive symptoms P2W; CESD-R | Prevalence | 32.20% | NA | NA | NA | NA | NA | NA | NA |
| To 2020 | United States | 620 | Study of Transition, Outcomes, and Gender (STRONG) | 18+ | NA | Depression; CES-D | Prevalence | 42.90% | NA | NA | NA | NA | NA | NA | NA |
| Valente 2020 | United States | 330 | Project AFFIRM | 16+ | NA | Depressive symptoms P1W; Brief Symptom Inventory-18 | Prevalence | 34.20% | 40.50% | 28.00% | NA | NA | NA | NA | NA |
| Vance 2021 | United States | 356 | 2015-2017 Biennial California Healthy Kids Survey | Grades 9 and 11 students | NA | Depression symptoms P12M; self-report | Prevalence | Black and Latinx: 50.0%; White: 63.0% | NA | NA | NA | NA | NA | NA | NA |
| vanHeesewijk 2021 | Netherlands | 37 | No study name provided | 55-70 | NA | Depression symptoms; Hopkins Symptom Checklist-25 | Prevalence | NA | NA | NA | 14.70% | NA | NA | NA | NA |
| Wang 2020b. | China | 397 | No study name provided | 19+ | TW SW | Depression symptoms; CES-D | Prevalence | NA | NA | NA | 49.10% | NA | NA | NA | NA |
| Wang 2021a. | United States | 2890 | Musculoskeletal diagnosis (MSD) cohort | 18+ | NA | Depression; diagnosis | Prevalence | 49.20% | NA | NA | NA | NA | NA | NA | NA |
| Wang 2021b. | China | 198 | No study name provided | 18+ | Sexual intercourse in P6M | Depressive symptoms P2W; PHQ-9 | Prevalence | Moderate: 17.2%; moderately severe: 4.5%; severe: 3.5% | NA | NA | Moderate: 17.2%; moderately severe: 4.5%; severe: 3.5% | NA | NA | NA | NA |
| Wathelet 2020 | France | 784 | COSAMe | NA | University students during COVID-19 pandemic | Depression severity; BDI | Prevalence | NA | NA | NA | NA | NA | NA | NA | Moderate: 35.5%; severe: 42.3% |
| Wichaidit 2021 | Thailand | 755 | National School Survey on Alcohol Consumption, Substance Use and Other Health-Risk Behaviors | Years 7, 9 and 11 students | NA | Depressive experience P12M; self-report | Prevalence | NA | NA | NA | 19.10% | 22.60% | NA | NA | NA |
| Witte 2020 | United States; United Kingdom | 32 | No study name provided | 18+ | LGBTQ+ veterinary professionals | Depressive episodes ever; self-report | Prevalence | 50.00% | NA | NA | NA | NA | NA | NA | NA |
| Yan 2021 | China | 222 | No study name provided | 18+ | NA | Depression; self-report of physician diagnosis | Prevalence | NA | NA | NA | 33.30% | NA | NA | NA | NA |
| Yi 2020 | Cambodia | 1039 | No study name provided | 18+ | NA | Depressive symptoms; CES-D | Prevalence | NA | NA | NA | 43.70% | NA | NA | NA | NA |
| Zea 2021 | Colombia | 58 | No study name provided | 18-49 | NA | Depressive symptoms P1W; self-report | Prevalence | NA | NA | NA | 8.70% | NA | NA | NA | NA |
| Zhang 2020 | China | 81 | No study name provided | 16+ | Patients seeking gender-affirming treatment | Depression; Symptom Checklist-90 | Prevalence | 27.10% | NA | NA | NA | NA | NA | NA | NA |
| Zwickl 2021a. | Australia | 1019 | No study name provided | 16+ | NA | Depression symptoms P2W; PHQ-9 | Prevalence | 61.10% | NA | NA | 53.80% | 59.90% | NA | NA | 74.30% |
| Zwickl 2021b. | Australia | 928 | No study name provided | 18+ | NA | Depression; self-report of physician diagnosis | Prevalence | 85.00% | NA | NA | NA | NA | NA | NA | NA |
| Drug use disorder | | | | | | | | | | | | | | | |
| Aguilar 2020 | Paraguay | 304 | No study name provided | 15+ | NA | High-risk cocaine use (1/week for 25 days P6M); self-report | Prevalence | NA | NA | NA | 29.30% | NA | NA | NA | NA |
| Aristegui 2021 | Argentina | 61 | TransViiV | 18+ | Newly diagnosed TW living with HIV initiating ART | Met criteria for drug abuse or dependence; DAST-10 | Prevalence | NA | NA | NA | 13.10% | NA | NA | NA | NA |
| Batchelder 2021 | United States | 257 | No study name provided | 18+ | NA | Positive screen on DAST for potential drug abuse; DAST | Prevalence | NA | NA | NA | 5.70% | 2.90% | NA | NA | NA |
| Boskey 2020 | United States | 158 | No study name provided | 14-35 | AFAB trans people with a diagnosis of GD, seeking top surgery | Marijuana use (in those aged <18 years); self-report | Prevalence | 23.00% | NA | NA | NA | NA | NA | NA | NA |
| Davy-Mendez 2021 | United States; Canada | 157 | NA-ACCORD | 18+ | PLHIV | Injection drug use ever; NA | Prevalence | 18.00% | NA | NA | NA | NA | NA | NA | NA |
| Eastwood 2021 | United States | 102 | Enhancing engagement and retention in quality HIV Care for transgender women of color initiative | 18-24 | HIV+ TW of color | Drug dependence; self-report | Prevalence | NA | NA | NA | 15.60% | NA | NA | NA | NA |
| Felner 2021 | United States | 144 | Growing Up Today Study | NA | NA | 12-month drug abuse/dependence; adapted from National Survey on Drug Use and Health | Prevalence | 14.60% | 13.30% | 15.50% | NA | NA | NA | NA | NA |
| Fish 2021a. | United States | 7045 | California Healthy Kids Survey (CHKS) | 12+ | NA | Marijuana use P1M; self-report | Prevalence | 26.10% | NA | NA | NA | NA | NA | NA | NA |
| Frost 2020 | United States | 8619 | No study name provided | NA | US veterans | Any drug use disorder; diagnosis code | Prevalence | 7.20% | NA | NA | NA | NA | NA | NA | NA |
| Frost 2020 | United States | 8619 | No study name provided | NA | US veterans | Opioid use disorder; diagnosis code | Prevalence | 1.50% | NA | NA | NA | NA | NA | NA | NA |
| Frost 2020 | United States | 8619 | No study name provided | NA | US veterans | Amphetamine use disorder; diagnosis code | Prevalence | 1.10% | NA | NA | NA | NA | NA | NA | NA |
| Frost 2020 | United States | 8619 | No study name provided | NA | US veterans | Cocaine use disorder; diagnosis code | Prevalence | 1.50% | NA | NA | NA | NA | NA | NA | NA |
| Frost 2020 | United States | 8619 | No study name provided | NA | US veterans | Cannabis use disorder; diagnosis code | Prevalence | 3.40% | NA | NA | NA | NA | NA | NA | NA |
| Frost 2020 | United States | 8619 | No study name provided | NA | US veterans | Sedative use disorder; diagnosis code | Prevalence | 0.30% | NA | NA | NA | NA | NA | NA | NA |
| Frost 2020 | United States | 8619 | No study name provided | NA | US veterans | Hallucinogen use disorder; diagnosis code | Prevalence | 0.00% | NA | NA | NA | NA | NA | NA | NA |
| Garg 2020 | Indonesia | 49 | No study name provided | 16-30 | NA | Use of injection drugs; self-report | Prevalence | NA | NA | NA | Injected drugs ever: 4.0%; injected drugs P1M: 2.0% | NA | NA | NA | NA |
| Harper 2021 | Kenya | 62 | No study name provided | 18+ | NA | Marijuana use daily; self-report | Prevalence | 1.80% | NA | NA | NA | NA | NA | NA | NA |
| Harper 2021 | Kenya | 62 | No study name provided | 18+ | NA | Miraa or khat use daily; self-report | Prevalence | 0.00% | NA | NA | NA | NA | NA | NA | NA |
| Hisle-Gorman 2021 | United States | 3754 | No study name provided | <18 at first contact | Youth in the military healthcare system | SUD; diagnosis | Prevalence | 6.30% | NA | NA | NA | NA | NA | NA | NA |
| Hughto 2021c. | United States | 15637 | OLDW (OptimLabs Data Warehouse) | 18+ | NA | Polysubstance SUD; diagnosis | Prevalence | 2.00% | NA | NA | NA | NA | NA | NA | NA |
| Hughto 2021c. | United States | 15637 | OLDW (OptimLabs Data Warehouse) | 18+ | NA | Drug use disorder diagnosis; diagnosis | Prevalence | 4.30% | NA | NA | NA | NA | NA | NA | NA |
| Kaltiala 2020 | Finland | 52 | No study name provided | 15-19 | NA | Substance abuse; diagnosis | Prevalence | 4.00% | NA | NA | NA | NA | NA | NA | NA |
| Katz-Wise 2021 | United States | 33 | Trans Teen and Family Narratives (TTFN) Project | 13-17 | NA | Marijuana use ever; self-report | Prevalence | 23.30% | NA | NA | NA | NA | NA | NA | NA |
| Katz-Wise 2021 | United States | 33 | Trans Teen and Family Narratives (TTFN) Project | 13-17 | NA | Any substance use; self-report | Prevalence | 17.20% | NA | NA | NA | NA | NA | NA | NA |
| Katz-Wise 2021 | United States | 33 | Trans Teen and Family Narratives (TTFN) Project | 13-17 | NA | Polysubstance use ever; self-report | Prevalence | 3.50% | NA | NA | NA | NA | NA | NA | NA |
| Khorashad 2021 | Iran | 205 | No study name provided | NA | NA | Substance abuse and addictive disorders; diagnosis | Prevalence | Current 0.6%; Lifetime 2.0% | NA | NA | Current: 0.0%; lifetime: 1.1% | Current: 1.2%; lifetime: 2.7% | NA | NA | NA |
| Kidd 2021a. | United States | NA | USTS | 18+ | NA | Prescription drug misuse P1M; self-report | Prevalence | NA | NA | NA | 13.50% | 17.30% | 18.00% | 18.70% | NA |
| Lacombe-Duncan 2021 | Canada | 54 | CHIWOS (Canadian HIV Women's Sexual and Reproductive Health Cohort Study) | 18+ | TW living with HIV | Injection drug use; self-report | Prevalence | NA | NA | NA | Current: 16.7%; Past: 16.7% | NA | NA | NA | NA |
| Mohr 2021 | Switzerland | 98 | No study name provided | NA | NA | Cannabis abuse; diagnosis | Prevalence | 9.20% | NA | NA | 5.70% | 48.90% | NA | NA | NA |
| Poteat 2020c. | United States | 1020 | LITE (American Cohort to Study HIV Acquisition Among Transgender Women in High Risk Areas) | 18+ | NA | SUD; self-report | Prevalence | NA | NA | NA | 29.40% | NA | NA | NA | NA |
| Progovac 2021 | United States | 916 | No study name provided | Dec-80 | NA | OUD; diagnosis | Prevalence | 2.60% | NA | NA | NA | NA | NA | NA | NA |
| Radusky 2020 | Argentina | 61 | No study name provided | 18+ | People initiating HIV care | Drug abuse; DAST | Prevalence | NA | NA | NA | 13.10% | NA | NA | NA | NA |
| Radusky 2021b. | Argentina | 41 | COPA2 (Conexiones y Opciones Positivas en la Argentina 2) | 18+ | Individuals disengaged from HIV care | Problematic substance use; DAST-10 | Prevalence | NA | NA | NA | 39.00% | NA | NA | NA | NA |
| Restar 2020a. | United States | 297 | Project LifeSkills | 16-29 | NA | SUD recent; self-report | Prevalence | NA | NA | NA | 14.60% | NA | NA | NA | NA |
| Rouhani 2021 | United States | 42 | Sapphire Study | 15+ | Street-based SW | Non-injection drug use daily P3M; self-report | Prevalence | NA | NA | NA | 37.20% | NA | NA | NA | NA |
| Rouhani 2021 | United States | 42 | Sapphire Study | 15+ | Street-based SW | Overdose P3M; self-report | Prevalence | NA | NA | NA | 4.70% | NA | NA | NA | NA |
| Sartaj 2021 | India | 50 | No study name provided | 18-60 | Hijra | Cannabis use; WHO-ASSIST | Prevalence | NA | Moderate: 4.0%; High: 2.0% | NA | NA | NA | NA | NA | NA |
| Schlissel 2021 | United States | 1567 | Survey of Today's Adolescent Relationships and Transitions | 13-24 | NA | Injection drug use ever; self-report | Prevalence | 29.40% | NA | NA | 60.30% | 4.90% | NA | NA | 3.90% |
| Segev-Becker 2020 | Israel | 106 | No study name provided | 18-Apr | NA | Cannabis and polysubstance abuse; diagnosis | Prevalence | NA | 16.00% | 1.50% | NA | NA | NA | NA | NA |
| She 2020 | Canada | 92 | No study name provided | NA | NA | SUD ever; diagnosis | Prevalence | Ever: 17.4%; Current: 13.0% | NA | NA | NA | NA | NA | NA | NA |
| Silva 2021 | Brazil | 189 | No study name provided | NA | NA | Illicit drug use; self-report | Prevalence | 35.50% | NA | NA | 32.10% | 40.30% | NA | NA | NA |
| Smeaton 2020 | United States; Canada; Spain; Brazil; Peru; Haiti; Thailand; India; South Africa; Uganda; Zimbabwe; Botswana | 129 | REPRIEVE | 40-75 | PLHIV | Acquired HIV via injection drug use; self-report | Prevalence | NA | 2.00% | 25.00% | NA | NA | NA | NA | NA |
| Spanos 2021 | Australia | 589 | No study name provided | 21+ | Patients receiving consultations for hormone therapy | Substance abuse/dependency; diagnosis | Prevalence | 3.10% | NA | NA | NA | NA | NA | NA | NA |
| Stanton 2021 | United States | 3179 | No study name provided | 18+ | NA | SUD; DAST | Prevalence | NA | NA | NA | 5.40% | 2.80% | 10.40% | 4.10% | NA |
| Steele 2020 | United Kingdom | 53 | No study name provided | NA | TGD SW | Inject IV drugs current; self-report | Prevalence | 3.80% | NA | NA | NA | NA | NA | NA | NA |
| Stogner 2021 | United States | 311 | YRBS | NA | Youth in seven urban school districts and four states | Synthetic cannabinoid use; self-report | Prevalence | District-level any synthetic cannabinoid use: 30.3%; District-level continuing synthetic cannabinoid use: 25.2%; State-level any synthetic cannabinoid use: 32.6%; State-level continuing synthetic cannabinoid use: 20.9% | NA | NA | NA | NA | NA | NA | NA |
| Strauss 2020a. | Australia | 869 | Trans Pathways | 14-25 | NA | SUD ever; self-report of physician diagnosis | Prevalence | 13.50% | 16.40% | 12.50% | NA | NA | NA | NA | NA |
| Strauss 2020b. | Australia | 859 | Trans Pathways | 14-25 | NA | SUD ever; self-report of physician diagnosis | Prevalence | 13.50% | NA | NA | NA | NA | NA | NA | NA |
| Strauss 2021 | Australia | 859 | Trans Pathways | 14-25 | NA | SUD ever; self-report of physician diagnosis | Prevalence | With ASD: 41.3%; without ASD: 5.4% | NA | NA | NA | NA | NA | NA | NA |
| Watson 2020 | United States | 3868 | LGBTQ National Teen Survey | 13-17 | NA | Marijuana use ever and P1M; self-report | Prevalence | NA | Ever: 33.3%; P1M: 19.9% | Ever: 35.6%; P1M: 18.8% | NA | NA | Ever: 32.1%; P1M: 13.6% | Ever: 25.3%; P1M: 12.4% | NA |
| Wichaidit 2021 | Thailand | 755 | National School Survey on Alcohol Consumption, Substance Use and Other Health-Risk Behaviors | Years 7, 9 and 11 students | NA | Marijuana use ever; self-report | Prevalence | NA | NA | NA | 6.20% | 8.90% | NA | NA | NA |
| Wichaidit 2021 | Thailand | 755 | National School Survey on Alcohol Consumption, Substance Use and Other Health-Risk Behaviors | Years 7, 9 and 11 students | NA | Kratom use ever; self-report | Prevalence | NA | NA | NA | 4.90% | 6.50% | NA | NA | NA |
| Wichaidit 2021 | Thailand | 755 | National School Survey on Alcohol Consumption, Substance Use and Other Health-Risk Behaviors | Years 7, 9 and 11 students | NA | Yaba (methamphetamine pills) use ever; self-report | Prevalence | NA | NA | NA | 0.70% | 3.40% | NA | NA | NA |
| Wichaidit 2021 | Thailand | 755 | National School Survey on Alcohol Consumption, Substance Use and Other Health-Risk Behaviors | Years 7, 9 and 11 students | NA | Crystal methamphetamine use ever; self-report | Prevalence | NA | NA | NA | 1.90% | 1.80% | NA | NA | NA |
| Williams 2021a. | United States | 1392 | No study name provided | 18+ | Veterans in VA care with documented unhealthy alcohol use | Non-alcohol SUD; diagnosis | Prevalence | 37.10% | NA | NA | NA | NA | NA | NA | NA |
| Williams 2021b. | United States | 8619 | No study name provided | 18+ | Veterans in VA care | Non-alcohol SUD; diagnosis | Prevalence | 19.10% | NA | NA | NA | NA | NA | NA | NA |
| Wilson 2021b. | United States | 116 | National HIV Behavioral Surveillance Study | 18+ | HIV-negative TW | Injection drug use P12M; self-report | Prevalence | NA | NA | NA | 8.60% | NA | NA | NA | NA |
| Wolfe 2021 | United States | 600 | Project VOICE NOW! | 18+ | NA | SUD ever; self-report of physician diagnosis | Prevalence | 11.80% | NA | NA | NA | NA | NA | NA | NA |
| Yousuf 2021 | Pakistan | 95 | No study name provided | NA | PLHIV or Hepatitis C | Chronic drug addiction; diagnosis | Prevalence | 26.30% | NA | NA | NA | NA | NA | NA | NA |
| Nicotine or tobacco use disorder | | | | | | | | | | | | | | | |
| Boskey 2020 | United States | 158 | No study name provided | 14-35 | AFAB trans people with a diagnosis of GD, seeking top surgery | Nicotine use (in those aged <18 years); self-report | Prevalence | 12.00% | NA | NA | NA | NA | NA | NA | NA |
| Felner 2021 | United States | 144 | Growing Up Today Study | NA | NA | 12-month nicotine dependence; items adapted from National Survey on Drug Use and Health | NA | 13.90% | 15.00% | 13.10% | NA | NA | NA | NA | NA |
| Ferlatte 2020a. | Canada | 209 | Sex Now Survey (2015) | NA | Trans MSM | Tobacco use daily; self-report | Prevalence | NA | NA | NA | NA | 15.30% | NA | NA | NA |
| Fish 2021a. | United States | 7045 | California Healthy Kids Survey (CHKS) | 12+ | NA | E-cigarette use P1M; self-report | Prevalence | 25.60% | NA | NA | NA | NA | NA | NA | NA |
| Fish 2021a. | United States | 7045 | California Healthy Kids Survey (CHKS) | 12+ | NA | Combustible cigarette use P1M; self-report | Prevalence | 17.20% | NA | NA | NA | NA | NA | NA | NA |
| Gamarel 2020 | United States | 3840 | LGBTQ Teen Study | 13-17 | NA | Cigarette smoking P1M; self-report | Prevalence | 8.40% | NA | NA | 7.10% | 14.20% | 6.10% | 6.50% | NA |
| Gava 2021 | Italy | 108 | No study name provided | 18+ | NA | Current cigarette smoking; self-report | Prevalence | 47.20% | NA | NA | NA | NA | NA | NA | NA |
| Harper 2021 | Kenya | 62 | No study name provided | 18+ | NA | Tobacco use daily; self-report | Prevalence | 5.60% | NA | NA | NA | NA | NA | NA | NA |
| Hawkins 2021 | United States | 81 | No study name provided | 18+ | Transmasculine patients on testosterone presenting for hysterectomy | Tobacco use; diagnosis | Prevalence | NA | NA | 17.00% | NA | NA | NA | NA | NA |
| Hughto 2021c. | United States | 15637 | OLDW (OptimLabs Data Warehouse) | 18+ | NA | Nicotine use disorder; diagnosis | Prevalence | 16.60% | NA | NA | NA | NA | NA | NA | NA |
| Katz-Wise 2021 | United States | 33 | Trans Teen and Family Narratives (TTFN) Project | 13-17 | NA | Tobacco use ever; self-report | Prevalence | 13.30% | NA | NA | NA | NA | NA | NA | NA |
| Lee 2020a. | South Korea | 255 | Rainbow Connection Project II - Korean Transgender Adults Health Study | 19+ | Diagnosed with gender identity disorder, ever used hormone therapy, or received any kind of gender affirmation surgery. | Current smoker; self-report | Prevalence | 27.70% | 20.40% | 38.60% | NA | NA | NA | NA | NA |
| LoSchiavo 2021 | United States | 44 | P18 | 22-23 | NA | Current smoker; self-report | Prevalence | 59.10% | NA | NA | NA | NA | NA | NA | NA |
| Madhavan 2020 | India | 200 | No study name provided | 18+ | NA | Medium or high nicotine dependence; Fagerstrom Addiction Scale | Prevalence | Smokers: 35.3%; Smokeless tobacco users: 74.7% | NA | NA | NA | NA | NA | NA | NA |
| Majumder 2020 | India | 120 | No study name provided | 18-70 | NA | Addiction to smoking | Prevalence | NA | 31.20% | 54.50% | NA | NA | NA | NA | NA |
| Mohr 2021 | Switzerland | 98 | No study name provided | NA | NA | Nicotine abuse; diagnosis | Prevalence | 34.70% | NA | NA | 22.60% | 48.90% | NA | NA | NA |
| Pharr 2021 | United States | 2827 | BRFSS (Behavioral Risk Factor Surveillance System) | 18+ | NA | Smoker current; self-report | Prevalence | 22.40% | NA | NA | NA | NA | NA | NA | NA |
| Progovac 2021 | United States | 916 | No study name provided | Dec-80 | NA | Tobacco use disorder; diagnosis | Prevalence | 9.90% | NA | NA | NA | NA | NA | NA | NA |
| Real 2021 | Brazil | 34 | No study name provided | 18+ | NA | Smoking; self-report | Prevalence | NA | NA | NA | 3.20% | NA | NA | NA | NA |
| Restar 2020a. | United States | 297 | Project LifeSkills | 16-29 | NA | Cigarette smoking daily; self-report | Prevalence | NA | NA | NA | 26.90% | NA | NA | NA | NA |
| Sartaj 2021 | India | 50 | No study name provided | 18-60 | Hijra | Tobacco use; WHO-ASSIST | Prevalence | NA | Moderate: 26.0%; High: 20.0% | NA | NA | NA | NA | NA | NA |
| Smeaton 2020 | United States; Canada; Spain; Brazil; Peru; Haiti; Thailand; India; South Africa; Uganda; Zimbabwe; Botswana | 129 | REPRIEVE | 40-75 | PLHIV | Smoking current; self-report | Prevalence | NA | 27.00% | 50.00% | NA | NA | NA | NA | NA |
| Stowell 2020 | United States | 143 | BRFSS | 55-79 | No personal history of lung cancer | Smoking current; self-report | Prevalence | 11.40% | NA | NA | NA | NA | NA | NA | NA |
| Suarez 2021 | United States | 131 | No study name provided | 21-64 | NA | Smoking current; self-report | Prevalence | NA | NA | 11.1%-34.0% (across number of adverse childhood experiences) | NA | NA | NA | NA | NA |
| Tantirattanakulchai 2021 | Thailand | 280 | No study name provided | 18+ | NA | Smoking; self-report | Prevalence | NA | NA | NA | 20.40% | NA | NA | NA | NA |
| vanHeesewijk 2021 | Netherlands | 37 | No study name provided | 55-70 | NA | Smoking; self-report | Prevalence | NA | NA | NA | 40.50% | NA | NA | NA | NA |
| Walker 2020 | New Zealand | 458 | Action for Smokefree 2025 Year 10 Snapshot Survey | Year 10 students | NA | Daily cigarette use; self-report | Prevalence | NA | NA | NA | NA | NA | NA | NA | 8.80% |
| Walker 2020 | New Zealand | 458 | Action for Smokefree 2025 Year 10 Snapshot Survey | Year 10 students | NA | Daily e-cigarette use; self-report | Prevalence | NA | NA | NA | NA | NA | NA | NA | 10.80% |
| Waterschoot 2021 | Belgium | 74 | No study name provided | 18+ | Transmasculine patients who had metoidioplasty | Active smoking; diagnosis | Prevalence | NA | NA | 25.70% | NA | NA | NA | NA | NA |
| Watson 2020 | United States | 3868 | LGBTQ National Teen Survey | 13-17 | NA | Cigarette use ever and P1M; self-report | Prevalence | NA | Ever: 19.1%; P1M: 7.1% | Ever: 34.3%; P1M: 14.2% | NA | NA | Ever: 26.4%; P1M: 6.4% | Ever: 22.0%; P1M: 6.6% | NA |
| Wichaidit 2021 | Thailand | 755 | National School Survey on Alcohol Consumption, Substance Use and Other Health-Risk Behaviors | Years 7, 9 and 11 students | NA | Smoker ever and smoked P12M (among smokers ever); self-report | Prevalence | NA | NA | NA | Ever: 13.2%; P12M: 67.0% | Ever: 20.9%; P12M: 56.9% | NA | NA | NA |
| Williams 2021a. | United States | 1392 | No study name provided | 18+ | Veterans in VA care with documented unhealthy alcohol use | Smoking current; self-report | Prevalence | 53.30% | NA | NA | NA | NA | NA | NA | NA |
| Zhang 2021 | United States | 114 | Social Justice Sexuality Project | 18+ | Gay and lesbian individuals | Smoking current; self-report | Prevalence | 44.70% | NA | NA | NA | NA | NA | NA | NA |
| Other mental health condition | | | | | | | | | | | | | | | |
| Anderssen 2020 | Norway | 115 | SHoT2018 | Full-time higher education students | NA | High or moderate anxiety and depression P2W; Hopkins Symptoms Checklist (HSCL-25) | Prevalence | Binary transgender; moderate: 21.4%; high: 50.0% | NA | NA | NA | NA | NA | NA | Moderate: 11.9%; high: 62.7% |
| Anderssen 2020 | Norway | 115 | SHoT2018 | Full-time higher education students | NA | Any mental disorder; self-report | Prevalence | Binary transgender: 57.0% | NA | NA | NA | NA | NA | NA | 59.00% |
| Anderssen 2020 | Norway | 115 | SHoT2018 | Full-time higher education students | NA | Self-harm ever; self-report | Prevalence | Binary transgender: 57.1% | NA | NA | NA | NA | NA | NA | 53.60% |
| Anderssen 2020 | Norway | 115 | SHoT2018 | Full-time higher education students | NA | Self-harm thoughts ever; self-report | Prevalence | Binary transgender: 57.1% | NA | NA | NA | NA | NA | NA | 58.00% |
| Andrew 2020 | NA | 155 | No study name provided | NA | NA | PTSD; PCL-5 | Prevalence | 62.60% | NA | NA | NA | NA | NA | NA | NA |
| Angoff 2021 | United States | 238 | 2019 State of Utah Prevention Needs Assessment (PNA) | 8th-12th grade | NA | NSSI P12M; self-report | Prevalence | 59.00% | NA | NA | NA | NA | NA | NA | NA |
| Anzani 2020 | Italy | 91 | No study name provided | None | NA | Personality disorder; diagnosis | Prevalence | 49.50% | NA | NA | 54.20% | 44.20% | NA | NA | NA |
| Arikawa 2021 | United States | 117 | No study name provided | 18-35 | NA | Exhibiting eating disorder pathology; Eating Disorder Examination Self-Report Questionnaire (EDE-Q) | Prevalence | NA | NA | NA | NA | 29.30% | NA | NA | 32.60% |
| Arikawa 2021 | United States | 117 | No study name provided | 18-35 | NA | At risk for disordered eating; Eating Attitudes Test (EAT-26) | Prevalence | NA | NA | NA | NA | 20.30% | NA | NA | 17.20% |
| Atteberry-Ash 2021 | United States | 396 | 2015 Healthy Kids Colorado Survey (HKCS) | 15-18 | NA | NSSI; self-report | Prevalence | 50.50% | 15.00% | 23.50% | NA | NA | NA | NA | Trans other: 26.5%; don't know: 35.0% |
| Boskey 2020 | United States | 158 | No study name provided | 14-35 | AFAB trans people with a diagnosis of GD, seeking top surgery | PTSD; self-report | Prevalence | 12.00% | NA | NA | NA | NA | NA | NA | NA |
| Boskey 2020 | United States | 158 | No study name provided | 14-35 | AFAB trans people with a diagnosis of GD, seeking top surgery | ADD/ADHD; self-report | Prevalence | 19.00% | NA | NA | NA | NA | NA | NA | NA |
| Boskey 2020 | United States | 158 | No study name provided | 14-35 | AFAB trans people with a diagnosis of GD, seeking top surgery | Autism spectrum; self-report | Prevalence | 4.00% | NA | NA | NA | NA | NA | NA | NA |
| Boskey 2020 | United States | 158 | No study name provided | 14-35 | AFAB trans people with a diagnosis of GD, seeking top surgery | OCD; self-report | Prevalence | 4.00% | NA | NA | NA | NA | NA | NA | NA |
| Boskey 2020 | United States | 158 | No study name provided | 14-35 | AFAB trans people with a diagnosis of GD, seeking top surgery | Bipolar disorder; self-report | Prevalence | 4.00% | NA | NA | NA | NA | NA | NA | NA |
| Branstrom 2020 | Sweden | NA | No study name provided | NA | NA | Outpatient visit for any mood disorder in 2015; diagnosis | Prevalence | 9.30% | NA | NA | NA | NA | NA | NA | NA |
| Bretherton 2021 | Australia | 928 | No study name provided | 18+ | NA | Autism spectrum; self-report | Prevalence | 15.00% | NA | NA | NA | NA | NA | NA | NA |
| Bretherton 2021 | Australia | 928 | No study name provided | 18+ | NA | ADHD ever; self-report | Prevalence | 11.00% | NA | NA | NA | NA | NA | NA | NA |
| Bretherton 2021 | Australia | 928 | No study name provided | 18+ | NA | Bipolar disorder; self-report | Prevalence | 8.00% | NA | NA | NA | NA | NA | NA | NA |
| Bryant-Genevier 2021 | United States | 147 | No study name provided | NA | State, Tribal, local, and territorial public health workers during the COVID-19 pandemic | PTSD symptoms P2W; 6-item Impact of Event Scale (IES-6) for PTSD | Prevalence | 65.50% | NA | NA | NA | NA | NA | NA | NA |
| Busby 2020 | United States | 87 | eBridge | 18+ | College students | NSSI P12M; self-report | Prevalence | 42.50% | NA | NA | NA | NA | NA | NA | NA |
| Cerel 2021 | United States | 2784 | TransLifeline | 18+ | NA | Mental health diagnoses (yes/no); self-report | Prevalence | 79.60% | NA | NA | NA | NA | NA | NA | NA |
| Cerel 2021 | United States | 2784 | TransLifeline | 18+ | NA | NSSI ever; self-report | Prevalence | 63.70% | NA | NA | NA | NA | NA | NA | NA |
| Chen 2020 | China | 250 | No study name provided | 18+ | NA | Probable PTSD; PTSD scale | Prevalence | NA | NA | NA | 24.00% | NA | NA | NA | NA |
| Cheung 2020 | Australia | 895 | No study name provided | NA | NA | Bipolar disorder; diagnosis | Prevalence | Adult binary: 3.5% | NA | NA | NA | NA | NA | NA | Adult non-binary: 3.0% |
| Cheung 2020 | Australia | 895 | No study name provided | NA | NA | PTSD; diagnosis | Prevalence | Adult binary: 3.9% | NA | NA | NA | NA | NA | NA | Adult non-binary: 7.1% |
| Cheung 2020 | Australia | 895 | No study name provided | NA | NA | Borderline personality disorder; diagnosis | Prevalence | Adult binary: 6.0% | NA | NA | NA | NA | NA | NA | Adult non-binary: 9.1% |
| Cheung 2020 | Australia | 895 | No study name provided | NA | NA | Obsessive compulsive disorder; diagnosis | Prevalence | Adult binary: 2.1% | NA | NA | NA | NA | NA | NA | Adult non-binary: 3.0% |
| Cheung 2020 | Australia | 895 | No study name provided | NA | NA | Eating disorder; diagnosis | Prevalence | Adult binary: 2.1% | NA | NA | NA | NA | NA | NA | Adult non-binary: 7.1% |
| Denby 2021 | United States | 427 | No study name provided | 18+ | Patients seeking GAHT | PTSD; diagnosis | Prevalence | 5.40% | NA | NA | NA | NA | NA | NA | NA |
| Denby 2021 | United States | 427 | No study name provided | 18+ | Patients seeking GAHT | Bipolar disorder; diagnosis | Prevalence | 8.40% | NA | NA | NA | NA | NA | NA | NA |
| Denby 2021 | United States | 427 | No study name provided | 18+ | Patients seeking GAHT | Schizophrenia; diagnosis | Prevalence | 0.50% | NA | NA | NA | NA | NA | NA | NA |
| Denby 2021 | United States | 427 | No study name provided | 18+ | Patients seeking GAHT | ADHD; diagnosis | Prevalence | 9.60% | NA | NA | NA | NA | NA | NA | NA |
| Dinger 2020 | United States | 2487 | National College Health Assessment | 18-25 | College students | Intentionally injured yourself ever; self-report | Prevalence | Transgender: 25.9% | NA | NA | NA | NA | NA | NA | Non-binary, non-trans: 23.4% |
| Flatt 2021 | United States | 441 | BRFSS (Behavioral Risk Factor Surveillance System) | 18+ | NA | Reported experiences of subjective cognitive decline; self-report | Prevalence | 17.30% | NA | NA | NA | NA | NA | NA | NA |
| Frost 2020 | United States | 8619 | No study name provided | NA | US veterans | Any mental health condition P12M; diagnosis code | Prevalence | 61.30% | NA | NA | NA | NA | NA | NA | NA |
| Gorrell 2021 | United States | 399 | No study name provided | NA | NA | Binge eating disorder; diagnosis | Prevalence | NA | 4.20% | 1.20% | NA | NA | NA | NA | 2.00% |
| Gorrell 2021 | United States | 399 | No study name provided | NA | NA | Anorexia nervosa; diagnosis | Prevalence | NA | 25.00% | 27.70% | NA | NA | NA | NA | 19.30% |
| Gorrell 2021 | United States | 399 | No study name provided | NA | NA | Avoidant-restrictive food intake disorder; diagnosis | Prevalence | NA | 6.90% | 0.00% | NA | NA | NA | NA | 3.70% |
| Gorrell 2021 | United States | 399 | No study name provided | NA | NA | Bulimia nervosa; diagnosis | Prevalence | NA | 6.90% | 6.00% | NA | NA | NA | NA | 6.10% |
| Gorrell 2021 | United States | 399 | No study name provided | NA | NA | Other specified feeding and eating disorder; diagnosis | Prevalence | NA | 11.10% | 16.90% | NA | NA | NA | NA | 17.20% |
| Grammer 2021 | United States | 234 | No study name provided | 18+ | College students | Probable anorexia nervosa diagnosis; SWED (Stanford-Washington University ED Screen) | Prevalence | 1.70% | NA | NA | NA | NA | NA | NA | NA |
| Grammer 2021 | United States | 234 | No study name provided | 18+ | College students | Probable clinical/subthreshold Bulimia Nervosa/BED diagnosis; SWED (Stanford-Washington University ED Screen) | Prevalence | 13.20% | NA | NA | NA | NA | NA | NA | NA |
| Harper 2021 | Kenya | 62 | No study name provided | 18+ | NA | Clinically Significant PTSD Symptoms; Primary Care Post-Traumatic Stress Disorder Assessment | Prevalence | 64.30% | NA | NA | NA | NA | NA | NA | NA |
| Heller 2021 | United States | 659 | National College Health Assessment | 18+ | College students | Eating disorder P12M; self-report | Prevalence | NA | NA | NA | 25.00% | 3.00% | NA | NA | 4.30% |
| Hickson 2020 | Europe | 1047 | The European MSM Internet Survey 2017 | Legal age of consent | NA | Severe anxiety and depression; Patient Health Questionnaire-4 | Prevalence | NA | NA | NA | NA | AMAB trans men: 6.7%  AFAB trans men: 22.7%  AFAB men: 16.7% | NA | NA | NA |
| Hisle-Gorman 2021 | United States | 3754 | No study name provided | <18 at first contact | Youth in the military healthcare system | Adjustment disorder; diagnosis | Prevalence | 44.90% | NA | NA | NA | NA | NA | NA | NA |
| Hisle-Gorman 2021 | United States | 3754 | No study name provided | <18 at first contact | Youth in the military healthcare system | ADHD; diagnosis | Prevalence | 29.80% | NA | NA | NA | NA | NA | NA | NA |
| Hisle-Gorman 2021 | United States | 3754 | No study name provided | <18 at first contact | Youth in the military healthcare system | Cognitive disorder; diagnosis | Prevalence | 3.70% | NA | NA | NA | NA | NA | NA | NA |
| Hisle-Gorman 2021 | United States | 3754 | No study name provided | <18 at first contact | Youth in the military healthcare system | Disorders usually diagnosed in infancy or childhood (incl. autism); diagnosis | Prevalence | 11.50% | NA | NA | NA | NA | NA | NA | NA |
| Hisle-Gorman 2021 | United States | 3754 | No study name provided | <18 at first contact | Youth in the military healthcare system | Impulse disorder; diagnosis | Prevalence | 1.60% | NA | NA | NA | NA | NA | NA | NA |
| Hisle-Gorman 2021 | United States | 3754 | No study name provided | <18 at first contact | Youth in the military healthcare system | Personality disorder; diagnosis | Prevalence | 2.30% | NA | NA | NA | NA | NA | NA | NA |
| Hisle-Gorman 2021 | United States | 3754 | No study name provided | <18 at first contact | Youth in the military healthcare system | Psychotic disorder; diagnosis | Prevalence | 9.70% | NA | NA | NA | NA | NA | NA | NA |
| Holloway 2021 | United States | 58 | No study name provided | 18+ | Military servicemembers | High PTSD symptomology; PTSD Checklist for DSM-5 | Prevalence | 31.00% | NA | NA | NA | NA | NA | NA | NA |
| Hughto 2020 | United States | 288 | Transgender Stress and Health Study | 18+ | NA | Non-suicidal self-injury | Prevalence | Before gender affirmation: 66.0%; after GA: 29.5% | NA | NA | NA | NA | NA | NA | NA |
| Hughto 2021a. | United States | 580 | No study name provided | 18+ | NA | PTSD; Primary Care PTSD Screen | Prevalence | 36.80% | NA | NA | NA | NA | NA | NA | NA |
| Hughto 2021b. | United States | 545 | No study name provided | 18+ | NA | PTSD symptoms; Primary Care PTSD Screen | Prevalence | 36.10% | NA | NA | NA | NA | NA | NA | NA |
| James 2020 | United States | 82 | Rochester Epidemiology Project | NA | Patients seeking gender-related healthcare | Personality disorder; diagnosis | Prevalence | NA | 26.50% | 12.90% | NA | NA | NA | NA | NA |
| James 2020 | United States | 82 | Rochester Epidemiology Project | NA | Patients seeking gender-related healthcare | Post-traumatic stress disorder; diagnosis | Prevalence | NA | 14.30% | 12.90% | NA | NA | NA | NA | NA |
| James 2020 | United States | 82 | Rochester Epidemiology Project | NA | Patients seeking gender-related healthcare | Bipolar disorder; diagnosis | Prevalence | NA | 14.30% | 9.70% | NA | NA | NA | NA | NA |
| James 2020 | United States | 82 | Rochester Epidemiology Project | NA | Patients seeking gender-related healthcare | Eating disorder; diagnosis | Prevalence | NA | 12.20% | 12.90% | NA | NA | NA | NA | NA |
| James 2020 | United States | 82 | Rochester Epidemiology Project | NA | Patients seeking gender-related healthcare | Schizophrenia; diagnosis | Prevalence | NA | 8.20% | 0.00% | NA | NA | NA | NA | NA |
| James 2020 | United States | 82 | Rochester Epidemiology Project | NA | Patients seeking gender-related healthcare | Autism; diagnosis | Prevalence | NA | 6.10% | 3.20% | NA | NA | NA | NA | NA |
| James 2020 | United States | 82 | Rochester Epidemiology Project | NA | Patients seeking gender-related healthcare | NSSI; diagnosis | Prevalence | NA | 26.50% | 35.50% | NA | NA | NA | NA | NA |
| Kaltiala 2020 | Finland | 52 | No study name provided | 15-19 | NA | Conduct problems/anti-social; diagnosis | Prevalence | 14.00% | NA | NA | NA | NA | NA | NA | NA |
| Kaltiala 2020 | Finland | 52 | No study name provided | 15-19 | NA | Psychotic symptoms/psychosis; diagnosis | Prevalence | 2.00% | NA | NA | NA | NA | NA | NA | NA |
| Kaltiala 2020 | Finland | 52 | No study name provided | 15-19 | NA | Autism; diagnosis | Prevalence | 12.00% | NA | NA | NA | NA | NA | NA | NA |
| Kaltiala 2020 | Finland | 52 | No study name provided | 15-19 | NA | ADHD; diagnosis | Prevalence | 10.00% | NA | NA | NA | NA | NA | NA | NA |
| Kaltiala 2020 | Finland | 52 | No study name provided | 15-19 | NA | Eating disorder; diagnosis | Prevalence | 2.00% | NA | NA | NA | NA | NA | NA | NA |
| Kattari 2020a. | United States | 659 | Michigan Trans Health Survey | 18+ | NA | NSSI thoughts P12M; self-report | Prevalence | 49.90% | NA | NA | NA | NA | NA | NA | NA |
| Kattari 2020a. | United States | 659 | Michigan Trans Health Survey | 18+ | NA | NSSI P12M; self-report | Prevalence | 26.30% | NA | NA | NA | NA | NA | NA | NA |
| Khorashad 2021 | Iran | 205 | No study name provided | NA | NA | Trauma and stressor-related disorders; diagnosis | Prevalence | Current 17.5%; Lifetime 8.8% | NA | NA | Current: 15.3%; lifetime: 8.4% | Current: 19.5%; lifetime: 9.1% | NA | NA | NA |
| Khorashad 2021 | Iran | 205 | No study name provided | NA | NA | Obsessive compulsive and related disorders; diagnosis | Prevalence | Current 7.1%; Lifetime 0.5% | NA | NA | Current: 5.6%; lifetime: 1.1% | Current: 8.5%; lifetime: 0.0% | NA | NA | NA |
| Khorashad 2021 | Iran | 205 | No study name provided | NA | NA | Bipolar and related disorders; diagnosis | Prevalence | Current 5.2%; Lifetime 1.0% | NA | NA | Current: 8.3%; lifetime: 2.1% | Current: 1.2%; lifetime: 0.9% | NA | NA | NA |
| Khorashad 2021 | Iran | 205 | No study name provided | NA | NA | Schizophrenia and other psychotic disorders; diagnosis | Prevalence | Current 0.6%; Lifetime 1.5% | NA | NA | Current: 0.0%; lifetime: 2.1% | Current: 1.2%; lifetime: 0.9% | NA | NA | NA |
| Khorashad 2021 | Iran | 205 | No study name provided | NA | NA | Neurodevelopmental disorder ever; diagnosis | Prevalence | 2.00% | NA | NA | 1.10% | 2.70% | NA | NA | NA |
| Khorashad 2021 | Iran | 205 | No study name provided | NA | NA | Conduct disorder ever; diagnosis | Prevalence | 0.50% | NA | NA | 1.10% | 0.00% | NA | NA | NA |
| Khorashad 2021 | Iran | 205 | No study name provided | NA | NA | Neurocognitive disorder ever; diagnosis | Prevalence | 0.50% | NA | NA | 1.10% | 0.00% | NA | NA | NA |
| Khorashad 2021 | Iran | 205 | No study name provided | NA | NA | Personality disorder current; diagnosis | Prevalence | 23.40% | NA | NA | 30.00% | 17.00% | NA | NA | NA |
| Kirby 2020 | United States | 26 | No study name provided | 18+ | College students | Anorexia; self-report | Prevalence | 7.70% | NA | NA | NA | NA | NA | NA | NA |
| Kirby 2020 | United States | 26 | No study name provided | 18+ | College students | Bulimia; self-report | Prevalence | 19.00% | NA | NA | NA | NA | NA | NA | NA |
| Kirby 2020 | United States | 26 | No study name provided | 18+ | College students | Eating disorders (other than anorexia/bulimia); self-report | Prevalence | 15.00% | NA | NA | NA | NA | NA | NA | NA |
| Konrad 2020 | Germany | 585 | Disease Analyzer database (IQVIA) | M=34.8 | NA | Reaction to severe stress and adjustment disorders; diagnosis | Prevalence | 10.00% | NA | NA | NA | NA | NA | NA | NA |
| Konrad 2020 | Germany | 585 | Disease Analyzer database (IQVIA) | M=34.8 | NA | Somatoform disorders; diagnosis | Prevalence | 9.70% | NA | NA | NA | NA | NA | NA | NA |
| Kuper 2020 | United States | 148 | No study name provided | NA | NA | NSSI; diagnosis | Prevalence | Ever: 52.0%; P3M: 10.0% | NA | NA | NA | NA | NA | NA | NA |
| Lacombe-Duncan 2021 | Canada | 54 | CHIWOS (Canadian HIV Women's Sexual and Reproductive Health Cohort Study) | 18+ | TW living with HIV | Clinically significant PTSD symptoms; PTSD-C | Prevalence | NA | NA | NA | 45.30% | NA | NA | NA | NA |
| Lehmann 2020 | Northern Ireland | NA | No study name provided | 16+ | NA | Autism traits; Autism Questionnaire and RAADS-14 | Prevalence | 17.20% | NA | NA | NA | NA | NA | NA | NA |
| Leon 2021 | United States | 185 | Gender Wellness Center Pediatric Patient Registry | 25-Jul | NA | NSSI ever; diagnosis | Prevalence | 36.80% | NA | NA | NA | NA | NA | NA | NA |
| Leon 2021 | United States | 185 | Gender Wellness Center Pediatric Patient Registry | 25-Jul | NA | Mood disorder ever; diagnosis | Prevalence | 61.10% | NA | NA | NA | NA | NA | NA | NA |
| Levit 2021 | Israel | 115 | No study name provided | NA | NA | Eating disorder; diagnosis | Prevalence | NA | NA | NA | 36.30% | 5.80% | NA | NA | NA |
| Levit 2021 | Israel | 115 | No study name provided | NA | NA | PTSD; diagnosis | Prevalence | NA | NA | NA | 18.80% | 11.70% | NA | NA | NA |
| Levit 2021 | Israel | 115 | No study name provided | NA | NA | ADHD; diagnosis | Prevalence | NA | NA | NA | 9.00% | 0.00% | NA | NA | NA |
| Lim 2021 | Australia | 134 | No study name provided | 18+ | NA | Mental health condition; self-report of physician diagnosis | Prevalence | 81.00% | NA | NA | NA | NA | NA | NA | NA |
| Lim 2021 | Australia | 134 | No study name provided | 18+ | NA | Self-diagnosed mental health condition; self-report | Prevalence | 5.00% | NA | NA | NA | NA | NA | NA | NA |
| Linsenmeyer 2021 | United States | 164 | No study name provided | 23-Dec | NA | Eating disorder; self-report of physician diagnosis | Prevalence | 8.70% | NA | NA | NA | NA | NA | NA | NA |
| Linsenmeyer 2021 | United States | 164 | No study name provided | 23-Dec | NA | Anorexia nervosa or bulimia nervosa; Sick, Control, One Stone, Fat, Food Questionnaire (SCOFF) | Prevalence | 28.00% | NA | NA | NA | NA | NA | NA | NA |
| Linsenmeyer 2021 | United States | 164 | No study name provided | 23-Dec | NA | Binge eating; Adolescent Binge Eating Disorder Questionnaire | Prevalence | 9.10% | NA | NA | NA | NA | NA | NA | NA |
| Linsenmeyer 2021 | United States | 164 | No study name provided | 23-Dec | NA | Avoidant/restrictive eating disorder; Avoidant/Restrictive Food Intake Disorder Screen | Prevalence | 75.00% | NA | NA | NA | NA | NA | NA | NA |
| Majumder 2020 | India | 120 | No study name provided | 18-70 | NA | Depression and/or anxiety; diagnosis | Prevalence | 3.80% | NA | NA | NA | NA | NA | NA | NA |
| McNichols 2020 | United States | 246 | No study name provided | NA | NA | Social phobia ever; self-report | Prevalence | NA | NA | NA | NA | 23.00% | NA | NA | NA |
| McNichols 2020 | United States | 246 | No study name provided | NA | NA | Panic disorder ever; self-report | Prevalence | NA | NA | NA | NA | 18.00% | NA | NA | NA |
| McNichols 2020 | United States | 246 | No study name provided | NA | NA | Obsessive compulsive disorder ever; self-report | Prevalence | NA | NA | NA | NA | 16.00% | NA | NA | NA |
| McNichols 2020 | United States | 246 | No study name provided | NA | NA | Self-harm ever; self-report | Prevalence | NA | NA | NA | NA | 0.80% | NA | NA | NA |
| McNichols 2020 | United States | 246 | No study name provided | NA | NA | Eating disorder pure; self-report | Prevalence | NA | NA | NA | NA | 0.80% | NA | NA | NA |
| ModregoPardo 2020 | Spain | 190 | No study name provided | NA | NA | NSSI ideation; diagnosis | Prevalence | 35.00% | NA | NA | 28.00% | 39.00% | NA | NA | NA |
| ModregoPardo 2020 | Spain | 190 | No study name provided | NA | NA | NSSI behavior; diagnosis | Prevalence | 31.00% | NA | NA | 23.00% | 36.00% | NA | NA | NA |
| Murphy 2020 | NA | 124 | No study name provided | NA | NA | Autism; AQ-50 | Prevalence | 40.30% | NA | NA | 22.90% | 51.30% | NA | NA | NA |
| Nagata 2020 | United States | 484 | PRIDE study | 18+ | NA | Eating disorder ever; self-report diagnosis | Prevalence | NA | NA | NA | 8.10% | 10.60% | NA | NA | NA |
| Nagata 2020 | United States | 484 | PRIDE study | 18+ | NA | Dietary restraint P1M; self-report | Prevalence | NA | NA | NA | Any: 25.5%; Regular: 6.7% | Any: 27.9%; Regular: 9.9% | NA | NA | NA |
| Nagata 2020 | United States | 484 | PRIDE study | 18+ | NA | Binge eating P1M; self-report | Prevalence | NA | NA | NA | Any: 11.2%; Regular: 6.4% | Any: 12.8%; Regular: 4.7% | NA | NA | NA |
| Nagata 2020 | United States | 484 | PRIDE study | 18+ | NA | Self-induced vomiting P1M; self-report | Prevalence | NA | NA | NA | Any: 1.6%; Regular: 1.0% | Any: 1.7%; Regular: 1.2% | NA | NA | NA |
| Nagata 2020 | United States | 484 | PRIDE study | 18+ | NA | Laxative misuse P1M; self-report | Prevalence | NA | NA | NA | Any: 0.3%; Regular: 0.3% | Any: 0.6%; Regular: 0.6% | NA | NA | NA |
| Nagata 2020 | United States | 484 | PRIDE study | 18+ | NA | Excessive exercise P1M; self-report | NA | NA | NA | NA | Any: 8.0%; Regular: 1.0% | Any: 8.1%; Regular: 1.7% | NA | NA | NA |
| Nobili 2020 | United Kingdom | 118 | No study name provided | NA | NA | Autism; Autism Spectrum Quotient, short version | Prevalence | 34.70% | 32.20% | 37.30% | NA | NA | NA | NA | NA |
| Nowaskie 2021 | United States | 166 | No study name provided | NA | NA | Eating disorders ever; self-report diagnosis | Prevalence | 13.90% | NA | NA | 16.10% | 11.40% | NA | NA | NA |
| Parr 2020 | United States | 1523 | Healthy Minds Study | NA | College students | NSSI P12M; self-report | Prevalence | NA | NA | NA | 62.00% | NA | NA | NA | NA |
| Peterson 2020a. | United States | 249 | No study name provided | NA | NA | Binge eating P1M; self-report | Prevalence | 23.40% | NA | NA | 24.60% | 20.60% | NA | NA | NA |
| Peterson 2020a. | United States | 249 | No study name provided | NA | NA | Self-induced vomiting P1M; self-report | Prevalence | 3.90% | NA | NA | 2.90% | 3.90% | NA | NA | NA |
| Peterson 2020b. | United States | 86 | No study name provided | 26-Oct | NA | Binge eating P1M; EDE-Q | Prevalence | 25.60% | NA | NA | NA | NA | NA | NA | NA |
| Peterson 2020b. | United States | 86 | No study name provided | 26-Oct | NA | NSSI ever; self-report | Prevalence | 62.00% | NA | NA | NA | NA | NA | NA | NA |
| Polonijo 2020 | United States | 90 | Inland Empire Transgender Health and Wellness Profile, 2015 | NA | NA | Depression or anxiety disorder; self-report | Prevalence | 67.80% | NA | NA | NA | NA | NA | NA | NA |
| Poteat 2020c. | United States | 1020 | LITE (American Cohort to Study HIV Acquisition Among Transgender Women in High Risk Areas) | 18+ | NA | PTSD; self-report | Prevalence | NA | NA | NA | 41.00% | NA | NA | NA | NA |
| Progovac 2021 | United States | 916 | No study name provided | Dec-80 | NA | Schizophrenia or psychosis; diagnosis | Prevalence | 9.00% | NA | NA | NA | NA | NA | NA | NA |
| Progovac 2021 | United States | 916 | No study name provided | Dec-80 | NA | Bipolar disorder; diagnosis | Prevalence | 18.70% | NA | NA | NA | NA | NA | NA | NA |
| Restar 2020a. | United States | 297 | Project LifeSkills | 16-29 | NA | PTSD recent; self-report | Prevalence | NA | NA | NA | 8.50% | NA | NA | NA | NA |
| Sanfacon 2021 | United States | 74 | No study name provided | NA | Adults who were born or became deaf in both ears before 13 years of age | Depression or anxiety disorder; self-report diagnosis | Prevalence | 48.60% | NA | NA | NA | NA | NA | NA | 63.60% |
| Sartaj 2021 | India | 50 | No study name provided | 18-60 | Hijra | Somatoform disorder; self-report | Prevalence | NA | 6.00% | NA | NA | NA | NA | NA | NA |
| Sartaj 2021 | India | 50 | No study name provided | 18-60 | Hijra | Bulimia nervosa; self-report | Prevalence | NA | 2.00% | NA | NA | NA | NA | NA | NA |
| Segev-Becker 2020 | Israel | 106 | No study name provided | 18-Apr | NA | Eating disorder; diagnosis | Prevalence | NA | 8.00% | 3.50% | NA | NA | NA | NA | NA |
| Segev-Becker 2020 | Israel | 106 | No study name provided | 18-Apr | NA | Autism spectrum disorder; diagnosis | Prevalence | NA | 10.00% | NA | NA | NA | NA | NA | NA |
| Segev-Becker 2020 | Israel | 106 | No study name provided | 18-Apr | NA | ADD/ADHD; diagnosis | Prevalence | NA | 21.00% | 31.00% | NA | NA | NA | NA | NA |
| Sergi 2021 | United States | 631 | Trans* National Study | 18-75 | NA | PTSD ever; self-report diagnosis | Prevalence | NA | NA | NA | Filler users: 32.3%; Non-filler users: 34.2% | NA | NA | NA | NA |
| She 2020 | Canada | 92 | No study name provided | NA | NA | Mood disorder ever; diagnosis | Prevalence | Ever: 54.3%; Current: 38.0% | NA | NA | NA | NA | NA | NA | NA |
| She 2020 | Canada | 92 | No study name provided | NA | NA | Psychotic disorders ever; diagnosis | Prevalence | Ever: <5.4%; Current: <5.4% | NA | NA | NA | NA | NA | NA | NA |
| She 2020 | Canada | 92 | No study name provided | NA | NA | PTSD ever; diagnosis | Prevalence | Ever: 14.1%; Current: 7.6% | NA | NA | NA | NA | NA | NA | NA |
| She 2020 | Canada | 92 | No study name provided | NA | NA | Neurodevelopmental disorder ever; diagnosis | Prevalence | Ever: 20.7%; Current: 14.1% | NA | NA | NA | NA | NA | NA | NA |
| She 2020 | Canada | 92 | No study name provided | NA | NA | Eating disorder ever; diagnosis | Prevalence | Ever: <5.4%; Current: <5.4% | NA | NA | NA | NA | NA | NA | NA |
| Silveri 2021 | United States | 35 | No study name provided | 13-17 | Adolescents in residential psychiatric treatment | Bipolar disorder I and II; MINI-KID | Prevalence | I: 12.9%; II: 0.0% | NA | NA | NA | NA | NA | NA | NA |
| Silveri 2021 | United States | 35 | No study name provided | 13-17 | Adolescents in residential psychiatric treatment | Panic disorder; MINI-KID | Prevalence | 24.10% | NA | NA | NA | NA | NA | NA | NA |
| Silveri 2021 | United States | 35 | No study name provided | 13-17 | Adolescents in residential psychiatric treatment | Social phobia; MINI-KID | Prevalence | 60.00% | NA | NA | NA | NA | NA | NA | NA |
| Silveri 2021 | United States | 35 | No study name provided | 13-17 | Adolescents in residential psychiatric treatment | Obsessive Compulsive Disorder; MINI-KID | Prevalence | 12.50% | NA | NA | NA | NA | NA | NA | NA |
| Silveri 2021 | United States | 35 | No study name provided | 13-17 | Adolescents in residential psychiatric treatment | PTSD; MINI-KID | Prevalence | 25.80% | NA | NA | NA | NA | NA | NA | NA |
| Silveri 2021 | United States | 35 | No study name provided | 13-17 | Adolescents in residential psychiatric treatment | ADHD; MINI-KID | Prevalence | 18.80% | NA | NA | NA | NA | NA | NA | NA |
| Silveri 2021 | United States | 35 | No study name provided | 13-17 | Adolescents in residential psychiatric treatment | Anorexia; MINI-KID | Prevalence | 6.30% | NA | NA | NA | NA | NA | NA | NA |
| Silveri 2021 | United States | 35 | No study name provided | 13-17 | Adolescents in residential psychiatric treatment | Bulimia; MINI-KID | Prevalence | 6.30% | NA | NA | NA | NA | NA | NA | NA |
| Simone 2020 | United States | 238 | College Student Health Survey | 18+ | College students | Anorexia nervosa; self-report diagnosis | Prevalence | 5.10% | NA | NA | NA | NA | NA | NA | NA |
| Simone 2020 | United States | 238 | College Student Health Survey | 18+ | College students | Bulimia nervosa; self-report diagnosis | Prevalence | 1.70% | NA | NA | NA | NA | NA | NA | NA |
| Sorbara 2020 | Canada | 184 | No study name provided | 15+ | Patients in puberty under 18 | Self-harm ever; diagnosis | Prevalence | 40.00% | NA | NA | NA | NA | NA | NA | NA |
| Sorbara 2020 | Canada | 184 | No study name provided | 15+ | Patients in puberty under 18 | Autism spectrum disorder; diagnosis | Prevalence | 6.00% | NA | NA | NA | NA | NA | NA | NA |
| Spanos 2021 | Australia | 589 | No study name provided | 21+ | Patients receiving consultations for hormone therapy | ADHD; diagnosis | Prevalence | 6.90% | NA | NA | NA | NA | NA | NA | NA |
| Spanos 2021 | Australia | 589 | No study name provided | 21+ | Patients receiving consultations for hormone therapy | Borderline personality disorder; diagnosis | Prevalence | 5.80% | NA | NA | NA | NA | NA | NA | NA |
| Spanos 2021 | Australia | 589 | No study name provided | 21+ | Patients receiving consultations for hormone therapy | Autism spectrum disorder; diagnosis | Prevalence | 5.50% | NA | NA | NA | NA | NA | NA | NA |
| Spanos 2021 | Australia | 589 | No study name provided | 21+ | Patients receiving consultations for hormone therapy | PTSD; diagnosis | Prevalence | 3.10% | NA | NA | NA | NA | NA | NA | NA |
| Spanos 2021 | Australia | 589 | No study name provided | 21+ | Patients receiving consultations for hormone therapy | Schizophrenia; diagnosis | Prevalence | 2.40% | NA | NA | NA | NA | NA | NA | NA |
| Spanos 2021 | Australia | 589 | No study name provided | 21+ | Patients receiving consultations for hormone therapy | Bipolar disorder; diagnosis | Prevalence | 2.70% | NA | NA | NA | NA | NA | NA | NA |
| Spanos 2021 | Australia | 589 | No study name provided | 21+ | Patients receiving consultations for hormone therapy | Obsessive compulsive disorder; diagnosis | Prevalence | 1.00% | NA | NA | NA | NA | NA | NA | NA |
| Spanos 2021 | Australia | 589 | No study name provided | 21+ | Patients receiving consultations for hormone therapy | Eating disorder; diagnosis | Prevalence | 1.00% | NA | NA | NA | NA | NA | NA | NA |
| Spanos 2021 | Australia | 589 | No study name provided | 21+ | Patients receiving consultations for hormone therapy | Schizoaffective disorder; diagnosis | Prevalence | 0.70% | NA | NA | NA | NA | NA | NA | NA |
| Strang 2021a. | United States | 124 | No study name provided | 21-Nov | NA | Autism spectrum disorder; diagnosis | Prevalence | 28.20% | NA | NA | NA | NA | NA | NA | NA |
| Strang 2021b. | United States | 66 | No study name provided | 13-21 | Binary TGD individuals | Internalizing symptoms in clinical range; Achenbach System of Empirically Based Assessment | Prevalence | Youth with ASD: 51.0% | NA | NA | NA | NA | NA | NA | NA |
| Strang 2021b. | United States | 66 | No study name provided | 13-21 | Binary TGD individuals | Self-harm; Achenbach System of Empirically Based Assessment | Prevalence | Youth with ASD: ~39.0%; allistic youth: ~15.0% | NA | NA | NA | NA | NA | NA | NA |
| Strauss 2020a. | Australia | 869 | Trans Pathways | 14-25 | NA | Self-harm ever; self-report | Prevalence | 79.70% | 65.00% | 84.60% | NA | NA | NA | NA | NA |
| Strauss 2020a. | Australia | 869 | Trans Pathways | 14-25 | NA | PTSD ever; diagnosis | Prevalence | 25.10% | 21.00% | 26.50% | NA | NA | NA | NA | NA |
| Strauss 2020a. | Australia | 869 | Trans Pathways | 14-25 | NA | Personality disorder ever; self-report of physician diagnosis | Prevalence | 20.50% | 19.00% | 21.10% | NA | NA | NA | NA | NA |
| Strauss 2020a. | Australia | 869 | Trans Pathways | 14-25 | NA | Psychosis ever; self-report of physician diagnosis | Prevalence | 16.20% | 16.40% | 16.10% | NA | NA | NA | NA | NA |
| Strauss 2020a. | Australia | 869 | Trans Pathways | 14-25 | NA | Autism spectrum disorder; self-report of physician diagnosis | Prevalence | 22.50% | 25.10% | 21.60% | NA | NA | NA | NA | NA |
| Strauss 2020b. | Australia | 859 | Trans Pathways | 14-25 | NA | PTSD; self-report of physician diagnosis | Prevalence | 25.10% | NA | NA | NA | NA | NA | NA | NA |
| Strauss 2020b. | Australia | 859 | Trans Pathways | 14-25 | NA | Eating disorder ever; self-report of physician diagnosis | Prevalence | 22.70% | NA | NA | NA | NA | NA | NA | NA |
| Strauss 2020b. | Australia | 859 | Trans Pathways | 14-25 | NA | Personality disorder ever; self-report of physician diagnosis | Prevalence | 20.50% | NA | NA | NA | NA | NA | NA | NA |
| Strauss 2020b. | Australia | 859 | Trans Pathways | 14-25 | NA | Psychosis ever; self-report of physician diagnosis | Prevalence | 16.20% | NA | NA | NA | NA | NA | NA | NA |
| Strauss 2020b. | Australia | 859 | Trans Pathways | 14-25 | NA | Self-harm behavior P12M; self-report | Prevalence | 79.70% | NA | NA | NA | NA | NA | NA | NA |
| Strauss 2021 | Australia | 859 | Trans Pathways | 14-25 | NA | Autism spectrum disorder; self-report of physician diagnosis | Prevalence | 22.50% | NA | NA | NA | NA | NA | NA | NA |
| Strauss 2021 | Australia | 859 | Trans Pathways | 14-25 | NA | PTSD ever; self-report of physician diagnosis | Prevalence | With ASD: 57.0%; without ASD: 15.9% | NA | NA | NA | NA | NA | NA | NA |
| Strauss 2021 | Australia | 859 | Trans Pathways | 14-25 | NA | Personality disorder ever; self-report of physician diagnosis | Prevalence | With ASD: 51.2%; without ASD: 6.1% | NA | NA | NA | NA | NA | NA | NA |
| Strauss 2021 | Australia | 859 | Trans Pathways | 14-25 | NA | Psychosis ever; self-report of physician diagnosis | Prevalence | With ASD: 51.2%; without ASD: 6.1% | NA | NA | NA | NA | NA | NA | NA |
| Strauss 2021 | Australia | 859 | Trans Pathways | 14-25 | NA | Eating disorder ever; self-report of physician diagnosis | Prevalence | With ASD: 52.3%; without ASD: 14.2% | NA | NA | NA | NA | NA | NA | NA |
| Strauss 2021 | Australia | 859 | Trans Pathways | 14-25 | NA | Self-harm ever; self-report | Prevalence | With ASD: 85.1%; without ASD: 78.2% | NA | NA | NA | NA | NA | NA | NA |
| Suarez 2021 | United States | 131 | No study name provided | 21-64 | NA | Self-harm ever; self-report | Prevalence | NA | NA | 57.1%-81.4% (across number of adverse childhood experiences) | NA | NA | NA | NA | NA |
| Suarez 2021 | United States | 131 | No study name provided | 21-64 | NA | PTSD symptoms ever; Primary Care PTSD Screen | Prevalence | NA | NA | 14.3%-40.4% (across number of adverse childhood experiences) | NA | NA | NA | NA | NA |
| Tan 2021a. | Aotearoa/New Zealand | 49 | Counting Ourselves: Aotearoa New Zealand Trans and Non-Binary Health Survey | 14+ | Asian TGD individuals | Bipolar disorder; self-report of physician diagnosis | Prevalence | 13.50% | NA | NA | NA | NA | NA | NA | NA |
| Tan 2021a. | Aotearoa/New Zealand | 49 | Counting Ourselves: Aotearoa New Zealand Trans and Non-Binary Health Survey | 14+ | Asian TGD individuals | Binge eating P12M; self-report | Prevalence | 29.70% | NA | NA | NA | NA | NA | NA | NA |
| Tan 2021a. | Aotearoa/New Zealand | 49 | Counting Ourselves: Aotearoa New Zealand Trans and Non-Binary Health Survey | 14+ | Asian TGD individuals | NSSI P12M; self-report | Prevalence | 37.80% | NA | NA | NA | NA | NA | NA | NA |
| Tan 2021b. | Aotearoa/New Zealand | 1178 | Counting Ourselves: the Aotearoa New Zealand Trans and Non-Binary Health Survey | 14+ | NA | NSSI P12M; self-report | Prevalence | 42.00% | NA | NA | NA | NA | NA | NA | NA |
| Taylor 2020 | Australia | 474 | Who I Am Study | 18+ | Bisexual Australians | Bipolar disorder; self-report of physician diagnosis | Prevalence | 10.70% | NA | NA | NA | NA | NA | NA | NA |
| Taylor 2020 | Australia | 474 | Who I Am Study | 18+ | Bisexual Australians | Schizophrenia; self-report of physician diagnosis | Prevalence | 3.10% | NA | NA | NA | NA | NA | NA | NA |
| Taylor 2020 | Australia | 474 | Who I Am Study | 18+ | Bisexual Australians | Borderline personality disorder; self-report of physician diagnosis | Prevalence | 11.60% | NA | NA | NA | NA | NA | NA | NA |
| Taylor 2020 | Australia | 474 | Who I Am Study | 18+ | Bisexual Australians | Eating disorder; self-report of physician diagnosis | Prevalence | 12.60% | NA | NA | NA | NA | NA | NA | NA |
| Taylor 2020 | Australia | 474 | Who I Am Study | 18+ | Bisexual Australians | Dissociative identity disorder; self-report of physician diagnosis | Prevalence | 3.90% | NA | NA | NA | NA | NA | NA | NA |
| Taylor 2020 | Australia | 474 | Who I Am Study | 18+ | Bisexual Australians | PTSD; self-report of physician diagnosis | Prevalence | 17.70% | NA | NA | NA | NA | NA | NA | NA |
| Taylor 2020 | Australia | 474 | Who I Am Study | 18+ | Bisexual Australians | Obsessive compulsive disorder; self-report of physician diagnosis | Prevalence | 2.40% | NA | NA | NA | NA | NA | NA | NA |
| Taylor 2020 | Australia | 474 | Who I Am Study | 18+ | Bisexual Australians | ADHD; self-report of physician diagnosis | Prevalence | 2.70% | NA | NA | NA | NA | NA | NA | NA |
| Taylor 2020 | Australia | 474 | Who I Am Study | 18+ | Bisexual Australians | Self-harm ever and P24M; self-report | Prevalence | Ever: 75.1%; P24M: 35.0% | NA | NA | NA | NA | NA | NA | NA |
| Torres 2021 | Brazil | 86 | No study name provided | 18+ | NA | Mental health problems during COVID-19 social distancing; self-report | Prevalence | 88.40% | NA | NA | NA | NA | NA | NA | NA |
| Treharne 2020 | New Zealand; Australia | 392 | No study name provided | 18+ | NA | Self-harm ever and P1M; Deliberate Self-Harm Inventory | Prevalence | Ever: 88.0%; P1M: 36.9% | NA | NA | NA | NA | NA | NA | NA |
| Turner 2021 | Canada | NA | No study name provided | 18-Dec | NA | Deliberate self-harm; self-report | Prevalence | 68.00% | NA | NA | NA | NA | NA | NA | NA |
| VanCauwenberg 2021 | Belgium | 177 | No study name provided | 18-Dec | Referred to pediatric gender clinic | Self-harm behavior and/or 1+ suicide attempt P6M; item from Youth Self-Report | Prevalence | 32.10% | NA | NA | NA | NA | NA | NA | NA |
| Wang 2020a. | China | 2111 | No study name provided | Grades 7-11 students | NA | NSSI ideation P1M; self-report | Prevalence | NA | NA | NA | 27.30% | 33.50% | 25.90% | 31.30% | Questioning AMAB: 24.6%; questioning AFAB: 28.8% |
| Wang 2020a. | China | 2111 | No study name provided | Grades 7-11 students | NA | NSSI P1M; self-report | Prevalence | NA | NA | NA | 21.00% | 23.10% | 20.00% | 23.20% | Questioning AMAB: 17.3%; questioning AFAB: 19.8% |
| Wang 2021a. | United States | 2890 | Musculoskeletal diagnosis (MSD) cohort | 18+ | NA | PTSD; diagnosis | Prevalence | 19.40% | NA | NA | NA | NA | NA | NA | NA |
| Wathelet 2020 | France | 784 | COSAMe | NA | University students during COVID-19 pandemic | Distress arising from a traumatic life event; Impact of Event Scale-Revised | Prevalence | NA | NA | NA | NA | NA | NA | NA | Moderate: 14.3%; severe: 35.2% |
| Wathelet 2020 | France | 784 | COSAMe | NA | University students during COVID-19 pandemic | Perceived stress; Perceived Stress Scale | Prevalence | NA | NA | NA | NA | NA | NA | NA | Moderate: 44.4%; high: 47.4% |
| Wathelet 2021 | France | 337 | COSAMe | NA | University students during COVID-19 pandemic | Probable PTSD; PTSD Checklist for DSM-5 (French) | Prevalence | 36.50% | NA | NA | NA | NA | NA | NA | NA |
| Williams 2021a. | United States | 1392 | No study name provided | 18+ | Veterans in VA care with documented unhealthy alcohol use | Any mental health condition; diagnosis | Prevalence | 67.40% | NA | NA | NA | NA | NA | NA | NA |
| Williams 2021b. | United States | 8619 | No study name provided | 18+ | Veterans in VA care | Any mental health diagnosis; diagnosis | Prevalence | 61.30% | NA | NA | NA | NA | NA | NA | NA |
| Wilson 2021c. | Nepal | 200 | Sweekar | 18+ | NA | Diagnoses of both depression and anxiety P12M; self-report | Prevalence | 44.50% | NA | NA | NA | NA | NA | NA | NA |
| Wolford-Clevenger 2021b | United States | 38 | No study name provided | 18+ | NA | Probable diagnosis of PTSD; PTSD Checklist for DSM-5 | Prevalence | 42.10% | NA | NA | NA | NA | NA | NA | NA |
| Zwickl 2021a. | Australia | 1019 | No study name provided | 16+ | NA | Thoughts of self-harm or suicide P2W; PHQ-9 | Prevalence | 49.00% | NA | NA | 46.20% | 45.50% | NA | NA | 58.00% |
| Zwickl 2021b. | Australia | 928 | No study name provided | 18+ | NA | Intentional self-harm ever; self-report | Prevalence | 63.00% | NA | NA | NA | NA | NA | NA | NA |
| Psychological distress | | | | | | | | | | | | | | | |
| Becerra 2021 | United States | 1369 | USTS | 18+ | Asian Americans | Severe psychological distress P12M; Kessler-6 | Prevalence | 39.10% | NA | NA | 34.40% | 34.80% | NA | NA | 46.80% |
| Chen 2020 | China | 250 | No study name provided | 18+ | NA | Psychological distress P1M; Kessler-10 | Incidence | NA | NA | NA | Mild: 24.0%; moderate: 16.8%; severe: 26.8% | NA | NA | NA | NA |
| Gonzales 2020 | United States | 201 | No study name provided | 18-25 | College students | Frequent mental distress; self-report | Prevalence | Transgender: 79.1% | NA | NA | NA | NA | NA | NA | GNC: 69.2%; other: 76.3% |
| Harper 2021 | Kenya | 62 | No study name provided | 18+ | NA | Clinically Significant Levels of Psychological Distress (t-score > 62); Brief Symptom Inventory-18 (BSI-18) Global Severity Index | Prevalence | 12.70% | NA | NA | NA | NA | NA | NA | NA |
| Hibbert 2020 | United Kingdom | 500 | LGBT Sex and Lifestyles Survey | 18+ | NA | Psychological distress; Kessler 6 | Prevalence | 13.0% moderate, 25.0% high, 56.0% very high | NA | NA | NA | NA | NA | NA | NA |
| Hillman 2021 | United States | 3462 | USTS | 50+ | NA | Severe distress P1M; Kessler-6 | Prevalence | 12.20% | NA | NA | NA | NA | NA | NA | NA |
| Hughto 2021b. | United States | 545 | No study name provided | 18+ | NA | Global psychological distress; Brief Symptom Inventory | Prevalence | 12.10% | NA | NA | NA | NA | NA | NA | NA |
| Hunt 2021 | United States | 83 | No study name provided | 18+ | University students | Severe psychological distress; Kessler -6 | Prevalence | 48.20% | NA | NA | NA | NA | NA | NA | NA |
| Kattari 2020b. | United States | 20195 | USTS | 18+ | NA | Serious psychological distress; Kessler -6 | Prevalence | 37.70% | NA | NA | NA | NA | NA | NA | NA |
| Kcomt 2020a. | United States | 27715 | USTS | 18+ | NA | Serious psychological distress; Kessler -6 | Prevalence | 29.80% | NA | NA | NA | NA | NA | NA | NA |
| Kidd 2021a. | United States | NA | USTS | 18+ | NA | Serious psychological distress P1M; Kessler -6 | Prevalence | NA | NA | NA | 32.80% | 37.20% | 36.40% | 50.70% | NA |
| Kidd 2021b. | United States | 208 | Project AFFIRM | 18+ | NA | Cinically significant psychological distress during pandemic; Brief Symptom Inventory | Prevalence | 41.80% | NA | NA | NA | NA | NA | NA | NA |
| Lett 2020 | United States | 3151 | BRFSS (Behavioral Risk Factor Surveillance System) | NA | NA | Severe mental distress P1M; HRQOL | Prevalence | Black: 24.4%; White: 22.9% | NA | NA | NA | NA | NA | NA | NA |
| Maksut 2020 | United States | 381 | No study name provided | 15+ | NA | Severe psychological distress P1M; Kessler -6 | Prevalence | NA | NA | NA | 52.00% | NA | NA | NA | NA |
| Poteat 2020c. | United States | 1020 | LITE (American Cohort to Study HIV Acquisition Among Transgender Women in High Risk Areas) | 18+ | NA | Psychological distress; K6 | Prevalence | NA | NA | NA | 27.40% | NA | NA | NA | NA |
| Reisner 2020 | United States | 843 | No study name provided | 18+ | HIV-negative transmasculine people who had sex with cis men in P6M | Psychological distress P1M; K6 | Prevalence | NA | NA | Moderate: 27.5%; Severe: 19.8% | NA | NA | NA | NA | NA |
| Restar 2020b. | United States | 600 | No study name provided | 18+ | NA | Psychological distress P1W; Brief Symptom Inventory | Prevalence | 12.20% | NA | NA | NA | NA | NA | NA | NA |
| Scheim 2020 | United States | 22286 | USTS | 18+ | NA | Psychological distress P1M; K6 | Prevalence | 38.40% | NA | NA | NA | NA | NA | NA | NA |
| Seelman 2021 | United States | 7950 | USTS | 18+ | NA | Psychological distress current; K6 | Prevalence | NA | NA | NA | NA | 36.60% | NA | NA | NA |
| Swartz 2021 | United States | 223 | No study name provided | 18+ | TWLHIV | Severe psychological distress; Kessler -6 | Prevalence | NA | NA | NA | 28.30% | NA | NA | NA | NA |
| Tan 2020 | Aotearoa/New Zealand | 1178 | Counting Ourselves: the Aotearoa New Zealand Trans and Non-Binary Health Survey | 14+ | NA | High or very high psychological distress; Kessler -10 | Prevalence | 72.00% | NA | NA | NA | NA | NA | NA | NA |
| Tan 2021a. | Aotearoa/New Zealand | 49 | Counting Ourselves: Aotearoa New Zealand Trans and Non-Binary Health Survey | 14+ | Asian TGD individuals | Psychological distress P1M; Kessler -10 | Prevalence | 35.10% | NA | NA | NA | NA | NA | NA | NA |
| Tan 2021b. | Aotearoa/New Zealand | 1178 | Counting Ourselves: the Aotearoa New Zealand Trans and Non-Binary Health Survey | 14+ | NA | Very high psychological distress P1M; Kessler -10 | Prevalence | 46.20% | NA | NA | NA | NA | NA | NA | NA |
| Taylor 2020 | Australia | 474 | Who I Am Study | 18+ | Bisexual Australians | Psychological distress; Kessler -10 | Prevalence | High: 31.6%; very high: 40.1% | NA | NA | NA | NA | NA | NA | NA |
| Turban 2020 | United States | 3494 | USTS | 18-36 | TGD individuals who wanted puberty blockers | Severe psychological distress P1M; Kessler -6 | Prevalence | Received pubertal suppression: 37.2%; did not receive pubertal suppression: 55.1% | NA | NA | NA | NA | NA | NA | NA |
| Turban 2021 | United States | 9711 | USTS | 18+ | TGD and gender diverse people who had undergone social transition and realized they were TGD during childhood | Severe psychological distress P1M; Kessler-6 | Prevalence | 32.30% | NA | NA | NA | NA | NA | NA | NA |
| Valente 2020 | United States | 330 | Project AFFIRM | 16+ | NA | Gender psychological distress P1W; Brief Symptom Inventory-18 | Prevalence | NA | 39.90% | 36.60% | NA | NA | NA | NA | NA |
| Witte 2020 | United States; United Kingdom | 32 | No study name provided | 18+ | LGBTQ+ veterinary professionals | Serious psychological distress; Kessler-6 | Prevalence | 40.60% | NA | NA | NA | NA | NA | NA | NA |
| Suicidality | | | | | | | | | | | | | | | |
| Achille 2020 | United States | 50 | No study name provided | 25-Sep | NA | Suicidal ideation; PHQ-9 | Prevalence | 10.00% | 11.80% | 9.10% | NA | NA | NA | NA | NA |
| Allen 2021 | Australia | 142 | First Assessment Single-Session Triage (FASST) Study | 17-Aug | NA | High risk for suicide; Columbia Suicide Severity Rating Scale | Prevalence | 13.40% | NA | NA | NA | NA | NA | NA | NA |
| Anderssen 2020 | Norway | 115 | SHoT2018 | Full-time higher education students | NA | Suicide attempt ever; self-report | Prevalence | Binary transgender: 21.4% | NA | NA | NA | NA | NA | NA | 23.20% |
| Anderssen 2020 | Norway | 115 | SHoT2018 | Full-time higher education students | NA | Suicide thoughts ever; self-report | Prevalence | Binary transgender: 64.3% | NA | NA | NA | NA | NA | NA | 62.30% |
| Andrew 2020 | NA | 155 | No study name provided | NA | NA | High suicide risk; Suicide Behaviors Questionnaire-Revised | Prevalence | 71.00% | NA | NA | NA | NA | NA | NA | NA |
| Andrew 2020 | NA | 155 | No study name provided | NA | NA | Prior suicide attempt; Suicide Behaviors Questionnaire-Revised | Prevalence | 9.00% | NA | NA | NA | NA | NA | NA | NA |
| Andrew 2020 | NA | 155 | No study name provided | NA | NA | Likely to attempt suicide in future; Suicide Behaviors Questionnaire-Revised | Prevalence | 12.00% | NA | NA | NA | NA | NA | NA | NA |
| AndrewYockey 2020 | United States | 790 | USTS | NA | African Americans | Thought about suicide ever; self-report | Prevalence | 33.50% | NA | NA | 34.40% | 39.00% | 28.80% | 25.40% | Crossdressers: 44.4% |
| AndrewYockey 2020 | United States | 790 | USTS | NA | African Americans | Planned to attempt suicide ever; self-report | Prevalence | 20.00% | NA | NA | 19.70% | 25.00% | 10.80% | 15.80% | Crossdressers: 21.5% |
| AndrewYockey 2020 | United States | 790 | USTS | NA | African Americans | Attempted suicide ever; self-report | Prevalence | 38.00% | NA | NA | 31.00% | 41.10% | 26.10% | 45.60% | Crossdressers: 30.5% |
| Aristegui 2021 | Argentina | 61 | TransViiV | 18+ | Newly diagnosed TW living with HIV initiating ART | Suicide attempt ever; self-report | Prevalence | NA | NA | NA | 38.30% | NA | NA | NA | NA |
| Atteberry-Ash 2021 | United States | 396 | 2015 Healthy Kids Colorado Survey (HKCS) | 15-18 | NA | Suicide attempt; self-report | Prevalence | 28.30% | 20.50% | 27.70% | NA | NA | NA | NA | Trans other: 27.7%; don't know: 24.1% |
| Austin 2020 | United States; Canada | 372 | Project #Queery | 14-18 | NA | Pervious suicide attempt; single item from DSM-5 Self-Rated Level 1 Cross-Cutting Symptom Measure‚ Child | Prevalence | 56.00% | NA | NA | NA | NA | NA | NA | NA |
| Austin 2020 | United States; Canada | 372 | Project #Queery | 14-18 | NA | Suicidal ideation P6M; single item from DSM-5 Self-Rated Level 1 Cross-Cutting Symptom Measure‚ Child | Prevalence | 86.00% | NA | NA | NA | NA | NA | NA | NA |
| Becerra 2021 | United States | 1369 | USTS | 18+ | Asian Americans | Suicidal thoughts P12M; self-report | Prevalence | 81.80% | NA | NA | 79.40% | 84.70% | NA | NA | 81.40% |
| Becerra 2021 | United States | 1369 | USTS | 18+ | Asian Americans | Suicidal attempt P12M; self-report | Prevalence | 40.30% | NA | NA | 39.90% | 44.90% | NA | NA | 37.20% |
| Blosnich 2021 | United States | 8981 | No study name provided | NA | VA patients | Death by suicide; diagnosis | Period prevalence | 0.80% | NA | NA | NA | NA | NA | NA | NA |
| Boskey 2020 | United States | 158 | No study name provided | 14-35 | AFAB trans people with a diagnosis of GD, seeking top surgery | Suicidal ideation or self harm, P6M and ever; self-report | Prevalence | Ever: 64.0%; P6M: 13.0% | NA | NA | NA | NA | NA | NA | NA |
| Boyer 2021 | United States | 8112 | No study name provided | 18+ | VA patients | Death by suicide; diagnosis | Prevalence | 0.80% | NA | NA | NA | NA | NA | NA | NA |
| Branstrom 2020 | Sweden | NA | No study name provided | NA | NA | Suicide attempt resulting in hospitalization; diagnosis | Prevalence | 0.80% | NA | NA | NA | NA | NA | NA | NA |
| Bryant-Genevier 2021 | United States | 147 | No study name provided | NA | State, Tribal, local, and territorial public health workers during the COVID-19 pandemic | Suicidal ideation; one item of the PHQ-9 (indication that they would be better off dead or thought of hurting themselves at any time P2W) | Prevalence | 30.40% | NA | NA | NA | NA | NA | NA | NA |
| Busby 2020 | United States | 87 | eBridge | 18+ | College students | Suicidal ideation P12M; self-report | Prevalence | 44.80% | NA | NA | NA | NA | NA | NA | NA |
| Cerel 2021 | United States | 2784 | TransLifeline | 18+ | NA | Suicidal ideation P12M; self-report | Prevalence | 52.60% | NA | NA | NA | NA | NA | NA | NA |
| Cerel 2021 | United States | 2784 | TransLifeline | 18+ | NA | Suicide attempt P12M; self-report | Prevalence | 6.80% | NA | NA | NA | NA | NA | NA | NA |
| Cerel 2021 | United States | 2784 | TransLifeline | 18+ | NA | Suicidal ideation ever; self-report | Prevalence | 82.40% | NA | NA | NA | NA | NA | NA | NA |
| Cerel 2021 | United States | 2784 | TransLifeline | 18+ | NA | Suicide attempt ever; self-report | Prevalence | 6.80% | NA | NA | NA | NA | NA | NA | NA |
| Chen 2020 | China | 250 | No study name provided | 18+ | NA | Suicide ideation; self-report | Prevalence | NA | NA | NA | 22.00% | NA | NA | NA | NA |
| Chen 2020 | China | 250 | No study name provided | 18+ | NA | Prior suicide attempt; self-report | Prevalence | NA | NA | NA | 25.60% | NA | NA | NA | NA |
| Cheung 2020 | Australia | 895 | No study name provided | NA | NA | Previous suicide attempt; diagnosis | Prevalence | Adult binary: 9.4% | NA | NA | NA | NA | NA | NA | Adult non-binary: 16.2% |
| Cramer 2020 | United States | 45 | No study name provided | 18+ | Members of the National Coalition for Sexual Freedom, and on their listserv | Suicidal ideation ever; Suicidal Behaviors Questionnaire-Revised (SBQ-R) | Prevalence | 64.40% | NA | NA | NA | NA | NA | NA | NA |
| Cramer 2020 | United States | 45 | No study name provided | 18+ | Members of the National Coalition for Sexual Freedom, and on their listserv | Suicidal attempt ever; Suicidal Behaviors Questionnaire-Revised (SBQ-R) | Prevalence | 24.40% | NA | NA | NA | NA | NA | NA | NA |
| DelRio-Gonzalez 2021 | Colombia | 469 | No study name provided | Adults | NA | Suicidal ideation ever; item adapted from the Columbia Suicide Severity Rating Scale (C-SSRS) | Prevalence | NA | NA | NA | 48.60% | 68.90% | 69.40% | 81.00% | NA |
| DelRio-Gonzalez 2021 | Colombia | 469 | No study name provided | Adults | NA | Suicide planning ever; single item adapted from the Columbia Suicide Severity Rating Scale (C-SSRS) | Prevalence | NA | NA | NA | 50.00% | 67.60% | 73.10% | 77.00% | NA |
| DelRio-Gonzalez 2021 | Colombia | 469 | No study name provided | Adults | NA | Suicide attempt ever; single item adapted from the Columbia Suicide Severity Rating Scale (C-SSRS) | Prevalence | NA | NA | NA | 26.30% | 41.90% | 33.30% | 42.00% | NA |
| Denby 2021 | United States | 427 | No study name provided | 18+ | Patients seeking GAHT | Suicide attempt; diagnosis | Prevalence | 6.10% | NA | NA | NA | NA | NA | NA | NA |
| Dinger 2020 | United States | 2487 | National College Health Assessment | 18-25 | College students | Seriously considered suicide ever; self-report | Prevalence | Transgender: 32.9% | NA | NA | NA | NA | NA | NA | Non-binary, non-trans: 30.4% |
| Dinger 2020 | United States | 2487 | National College Health Assessment | 18-25 | College students | Attempted suicide ever; self-report | Prevalence | Transgender: 6.3% | NA | NA | NA | NA | NA | NA | Non-binary, non-trans: 6.2% |
| Dinger 2020 | United States | 2487 | National College Health Assessment | 18-25 | College students | Attempted suicide ever; self-report | Prevalence | Transgender: 6.3% | NA | NA | NA | NA | NA | NA | Non-binary, non-trans: 6.2% |
| Drescher 2021 | United States | 70 | No study name provided | 18+ | NA | Suicidal ideation ever; self-report | Prevalence | 52.90% | NA | NA | NA | NA | NA | NA | NA |
| Drescher 2021 | United States | 70 | No study name provided | 18+ | NA | Suicide attempt ever; self-report | Prevalence | 37.10% | NA | NA | NA | NA | NA | NA | NA |
| Fan 2021 | China | 220 | No study name provided | 18+ | SW | Suicidal ideation P12M; self-report | Prevalence | NA | NA | NA | 12.70% | NA | NA | NA | NA |
| Guzman-Gonzalez 2020 | Chile | 377 | No study name provided | 18+ | NA | Suicidal ideation P12M; self-report | Prevalence | 68.80% | NA | NA | NA | NA | NA | NA | NA |
| Guzman-Gonzalez 2020 | Chile | 377 | No study name provided | 18+ | NA | Suicide attempt P12M; self-report | Prevalence | 34.10% | NA | NA | NA | NA | NA | NA | NA |
| Halli 2021 | India | 282 | No study name provided | 18+ | NA | Thought or attempted suicide P1M; self-report | Prevalence | 42.30% | NA | NA | NA | NA | NA | NA | NA |
| Heino 2021 | Finland | 36 | Adolescent Mental Health Cohort and Replication Study | 15-16 | NA | Severe suicidal ideation; self-report | Prevalence | 14.30% | NA | NA | NA | NA | NA | NA | NA |
| Hickson 2020 | Europe | 1047 | The European MSM Internet Survey 2017 | Legal age of consent | NA | Suicidal ideation P2W; self-report | Prevalence | NA | NA | NA | NA | AMAB trans men: 26.9%  AFAB trans men: 50.1%  AFAB men: 33.0% | NA | NA | NA |
| Hillman 2021 | United States | 3462 | USTS | 50+ | NA | Suicide attempt ever; self-report | Prevalence | 27.90% | NA | NA | NA | NA | NA | NA | NA |
| Hisle-Gorman 2021 | United States | 3754 | No study name provided | <18 at first contact | Youth in the military healthcare system | Suicidal ideation or self-harm; diagnosis | Prevalence | 18.20% | NA | NA | NA | NA | NA | NA | NA |
| Horwitz 2020 | United States | NA | eBridge Study | 18+ | College students | Suicidal ideation; self-report | Prevalence | NA | NA | NA | 36.40% | 46.40% | 35.30% | 40.90% | NA |
| Horwitz 2020 | United States | NA | eBridge Study | 18+ | College students | Suicide attempt ever; self-report | Prevalence | NA | NA | NA | 24.20% | 30.90% | 24.50% | 23.80% | NA |
| Hughto 2020 | United States | 288 | Transgender Stress and Health Study | 18+ | NA | Contemplated suicide; self-report | Prevalence | Before gender affirmation: 73.3%; after GA: 43.4% | NA | NA | NA | NA | NA | NA | NA |
| Hughto 2020 | United States | 288 | Transgender Stress and Health Study | 18+ | NA | Attempted suicide; self-report | Prevalence | Before gender affirmation: 35.8%; after GA: 9.4% | NA | NA | NA | NA | NA | NA | NA |
| James 2020 | United States | 82 | Rochester Epidemiology Project | NA | Patients seeking gender-related healthcare | Suicide ideation; diagnosis | Prevalence | NA | 67.30% | 41.90% | NA | NA | NA | NA | NA |
| James 2020 | United States | 82 | Rochester Epidemiology Project | NA | Patients seeking gender-related healthcare | Suicide attempt; diagnosis | Prevalence | NA | 22.40% | 19.40% | NA | NA | NA | NA | NA |
| Jarrett 2021 | Multinational | 964 | COVID Disparities Working Group | 18+ | Hornet or Her app users | Suicidal ideation; self-report | Prevalence | Increased: 10.0%  Frequent: 7.6% | Increased: 11.6%  Frequent: 7.8% | Increased: 8.3%  Frequent: 8.3% | NA | NA | NA | NA | Increased: 9.5%  Frequent: 7.5% |
| Jin 2020 | United States | 297 | No study name provided | 16-29 | Report engaging in condomless sex | Suicidal ideation; self-report | Prevalence | NA | NA | NA | 18.90% | NA | NA | NA | NA |
| Joshi 2021 | India | 33 | No study name provided | 18+ | NA | High risk for suicidal behavior; Suicidal Behaviors Questionnaire-Revised | Prevalence | NA | NA | NA | 75.80% | NA | NA | NA | NA |
| Kaltiala 2020 | Finland | 52 | No study name provided | 15-19 | NA | Suicidality/self-harm during and before gender assessment; diagnosis | Prevalence | 35.00% | NA | NA | NA | NA | NA | NA | NA |
| Kattari 2020a. | United States | 659 | Michigan Trans Health Survey | 18+ | NA | Suicidal thoughts P12M; self-report | Prevalence | 45.40% | NA | NA | NA | NA | NA | NA | NA |
| Kattari 2020a. | United States | 659 | Michigan Trans Health Survey | 18+ | NA | Suicide attempt P12M; self-report | Prevalence | 7.70% | NA | NA | NA | NA | NA | NA | NA |
| Kattari 2020b. | United States | 20195 | USTS | 18+ | NA | Suicidal thoughts P12M; self-report | Prevalence | 48.40% | NA | NA | NA | NA | NA | NA | NA |
| Kittiteerasack 2020 | Thailand | 96 | No study name provided | 18-60 | NA | Suicidal ideation; self-report | Prevalence | Lifetime: 40.6%; P12M: 22.9% | NA | NA | NA | NA | NA | NA | NA |
| Kittiteerasack 2020 | Thailand | 96 | No study name provided | 18-60 | NA | Previous suicide attempt; self-report | Prevalence | 12.50% | NA | NA | NA | NA | NA | NA | NA |
| Kota 2020 | United States | 92 | No study name provided | 18-65 | NA | Suicidal ideation P12M; diagnosis | Prevalence | NA | NA | NA | 33.00% | NA | NA | NA | NA |
| Kuper 2020 | United States | 148 | No study name provided | NA | NA | Suicide attempt; diagnosis | Prevalence | Ever: 15.0%; P3M: 2.0% | NA | NA | NA | NA | NA | NA | NA |
| Lee 2020a. | South Korea | 255 | Rainbow Connection Project II - Korean Transgender Adults Health Study | 19+ | Diagnosed with gender identity disorder, ever used hormone therapy, or received any kind of gender affirmation surgery. | Suicidal ideation P12M; self-report | Prevalence | 53.80% | 56.90% | 49.00% | NA | NA | NA | NA | NA |
| Lee 2020a. | South Korea | 255 | Rainbow Connection Project II - Korean Transgender Adults Health Study | 19+ | Diagnosed with gender identity disorder, ever used hormone therapy, or received any kind of gender affirmation surgery. | Suicide attempt P12M.; self-report | Prevalence | 15.30% | 16.20% | 13.90% | NA | NA | NA | NA | NA |
| Lee 2020b. | South Korea | 207 | Rainbow Connection Project II - Korean Transgender Adults Health Study | 18+ | NA | Suicidal ideation P12M; self-report | Prevalence | 52.20% | NA | NA | 56.20% | 45.50% | NA | NA | NA |
| Lee 2020b. | South Korea | 207 | Rainbow Connection Project II - Korean Transgender Adults Health Study | 18+ | NA | Suicide attempts P12M; self-report | Prevalence | 15.00% | NA | NA | 17.70% | 10.40% | NA | NA | NA |
| Lee 2021 | South Korea | 557 | No study name provided | 18+ | NA | Suicidal ideation P12M; self-report | Prevalence | 63.00% | NA | NA | NA | NA | NA | NA | NA |
| Lee 2021 | South Korea | 557 | No study name provided | 18+ | NA | Suicide attempt P12M; self-report | Prevalence | 19.80% | NA | NA | NA | NA | NA | NA | NA |
| Leon 2021 | United States | 185 | Gender Wellness Center Pediatric Patient Registry | 25-Jul | NA | Suicidal ideation or attempt ever; diagnosis | Prevalence | 47.00% | NA | NA | NA | NA | NA | NA | NA |
| Liu 2020 | China | 1304 | No study name provided | M=22 | NA | Suicidal or self-injury ideation; self-report | Prevalence | 26.50% | NA | NA | 30.50% | 21.10% | NA | NA | NA |
| Liu 2020 | China | 1304 | No study name provided | M=22 | NA | Suicide attempt or self-injury; self-report | Prevalence | 16.60% | NA | NA | 20.00% | 12.00% | NA | NA | NA |
| Lozano-Verduzco 2021 | Mexico | 148 | No study name provided | NA | NA | Suicidal ideation P12M; self-report | Prevalence | 58.80% | NA | NA | NA | NA | NA | NA | NA |
| Lozano-Verduzco 2021 | Mexico | 148 | No study name provided | NA | NA | Suicide attempt P12M; self-report | Prevalence | 55.40% | NA | NA | NA | NA | NA | NA | NA |
| Mak 2020 | United States | 6327 | No study name provided | NA | NA | Crude rate of suicide attempts; diagnosis | Incidence | NA | 3.0 per 100,000 person-days | 4.8 per 100,000 person-days | NA | NA | NA | NA | NA |
| Maksut 2020 | United States | 381 | No study name provided | 15+ | NA | Suicidal ideation P12M; self-report | Prevalence | NA | NA | NA | 59.30% | NA | NA | NA | NA |
| Maksut 2020 | United States | 381 | No study name provided | 15+ | NA | Suicide attempt P12M; self-report | Prevalence | NA | NA | NA | 13.10% | NA | NA | NA | NA |
| McDowell 2020 | United States | 28980 | IBM MarketScan Commercial Database | 18+ | NA | Suicidality P12M; diagnosis | Prevalence | 1.3% to 7.6% across years and policy cohorts | NA | NA | NA | NA | NA | NA | NA |
| McInroy 2020 | United States; Canada | 2429 | No study name provided | 14-29 | NA | Attempted suicide ever; self-report | Prevalence | Age 14-19: 55.8%; Age 20-25: 47.5% | NA | NA | NA | NA | NA | NA | NA |
| McNichols 2020 | United States | 246 | No study name provided | NA | NA | Suicide attempt ever; self-report | Prevalence | NA | NA | NA | NA | 27.00% | NA | NA | NA |
| Mereish 2021 | United States | 265 | No study name provided | 24-Dec | NA | Suicide attempt; diagnosis | Prevalence | 41.70% | NA | NA | NA | NA | NA | NA | NA |
| Meyer 2021 | United States | 94 | Generations Study | 18-25, 34-41, 52-29 | NA | Suicidal ideation; Army Study to Assess Risk and Resilience in Service Members instrument | Prevalence | NA | NA | NA | NA | NA | NA | NA | Equality cohort (age 18-25): 94.2%  Visibility cohort (age 34-41): 92.1%  Pride cohort (age 52-59): 88.8% |
| Meyer 2021 | United States | 94 | Generations Study | 18-25, 34-41, 52-29 | NA | Suicide plan; Army Study to Assess Risk and Resilience in Service Members instrument | Prevalence | NA | NA | NA | NA | NA | NA | NA | Equality cohort (age 18-25): 82.8% Visibility cohort (age 34-41): 92.1%  Pride cohort (age 52-59): 70.2% |
| Meyer 2021 | United States | 94 | Generations Study | 18-25, 34-41, 52-29 | NA | Suicide attempt; Army Study to Assess Risk and Resilience in Service Members instrument | Prevalence | NA | NA | NA | NA | NA | NA | NA | Equality cohort (age 18-25): 34.5%  Visibility cohort (age 34-41): 40.0%  Pride cohort (age 52-59): n=6, percentage NR |
| ModregoPardo 2020 | Spain | 190 | No study name provided | NA | NA | Suicidal Ideation; diagnosis | Prevalence | 50.00% | NA | NA | 47.00% | 51.00% | NA | NA | NA |
| ModregoPardo 2020 | Spain | 190 | No study name provided | NA | NA | Suicide attempt; diagnosis | Prevalence | 21.00% | NA | NA | 21.00% | 21.00% | NA | NA | NA |
| Mohajer 2020 | Iran | 21 | No study name provided | NA | Patients without psychiatric comorbidity | Suicidal ideation; Beck's Scale for Suicidal Ideation (BSSI) | Prevalence | 80.90% | NA | NA | NA | NA | NA | NA | NA |
| Nematollahi 2021 | Iran | 127 | No study name provided | NA | NA | Suicidal ideation ever; self-report | Prevalence | NA | NA | NA | 71.70% | NA | NA | NA | NA |
| Nematollahi 2021 | Iran | 127 | No study name provided | NA | NA | Suicide attempt ever; self-report | Prevalence | NA | NA | NA | 50.40% | NA | NA | NA | NA |
| Newcomb 2020 | United States | 214 | RADAR and FAB 400 | NA | NA | Suicidal ideation P6M; self-report | Prevalence | 30.00% | NA | NA | 13.80% | 35.70% | 50.00% | 31.70% | NA |
| Newcomb 2020 | United States | 214 | RADAR and FAB 400 | NA | NA | Suicide plan P6M; self-report | Prevalence | 12.40% | NA | NA | 10.30% | 7.10% | 17.90% | 14.60% | NA |
| Newcomb 2020 | United States | 214 | RADAR and FAB 400 | NA | NA | Suicide attempt P6M; self-report | Prevalence | 6.70% | NA | NA | 12/1% | 0.00% | 14.30% | 3.70% | NA |
| Parr 2020 | United States | 1523 | Healthy Minds Study | NA | College students | Suicide ideation P12M; self-report | Prevalence | NA | NA | NA | 39.00% | NA | NA | NA | NA |
| Peterson 2020b. | United States | 86 | No study name provided | 26-Oct | NA | Suicidal ideation ever; self-report | Prevalence | 66.00% | NA | NA | NA | NA | NA | NA | NA |
| Peterson 2020b. | United States | 86 | No study name provided | 26-Oct | NA | Suicide attempt ever; self-report | Prevalence | 48.00% | NA | NA | NA | NA | NA | NA | NA |
| Platt 2020 | United States | 1963 | No study name provided | NA | College and university students accessing mental health services | Suicidal ideation ever; self-report | Prevalence | 62.60% | NA | NA | NA | NA | NA | NA | 65.50% |
| Platt 2020 | United States | 1963 | No study name provided | NA | College and university students accessing mental health services | Suicide attempt ever; self-report | Prevalence | 26.50% | NA | NA | NA | NA | NA | NA | 24.30% |
| Polonijo 2020 | United States | 90 | Inland Empire Transgender Health and Wellness Profile, 2015 | NA | NA | Suicide ideation ever; self-report | Prevalence | 74.40% | NA | NA | NA | NA | NA | NA | NA |
| Poteat 2020c. | United States | 1020 | LITE (American Cohort to Study HIV Acquisition Among Transgender Women in High Risk Areas) | 18+ | NA | Suicidal ideation P6M; self-report | Prevalence | NA | NA | NA | 28.00% | NA | NA | NA | NA |
| Price-Feeney 2020 | United States | 8367 | No study name provided | 13-24 | NA | Suicidal ideation P12M; self-report | Prevalence | 54.20% | NA | NA | 51.80% | 61.70% | 43.10% | 51.10% | NA |
| Price-Feeney 2020 | United States | 8367 | No study name provided | 13-24 | NA | Suicide attempt P12M; self-report | Prevalence | 28.60% | NA | NA | 27.00% | 34.70% | 23.10% | 25.10% | NA |
| Price-Feeney 2021 | United States | 7370 | No study name provided | 13-24 | NA | Suicidal ideation P12M.; self-report | Prevalence | 54.30% | NA | NA | NA | NA | NA | NA | NA |
| Price-Feeney 2021 | United States | 7370 | No study name provided | 13-24 | NA | Suicide attempt P12M; self-report | Prevalence | 29.10% | NA | NA | NA | NA | NA | NA | NA |
| Progovac 2020 | United States | 8696 | No study name provided | 18+ | Medicare beneficiaries | Suicide attempt; diagnosis | Prevalence | Disabled: 5.1%; 65+: 0.5% | NA | NA | NA | NA | NA | NA | NA |
| Progovac 2020 | United States | 8696 | No study name provided | 18+ | Medicare beneficiaries | Suicidal ideation; diagnosis | Prevalence | Disabled: 15.4%; 65+: 1.1% | NA | NA | NA | NA | NA | NA | NA |
| Progovac 2020 | United States | 8696 | No study name provided | 18+ | Medicare beneficiaries | Any suicidality; diagnosis | Prevalence | Disabled: 18.5%; 65+: 2.9% | NA | NA | NA | NA | NA | NA | NA |
| Progovac 2021 | United States | 916 | No study name provided | 12-80 | NA | Suicide attempt; diagnosis | Prevalence | 5.20% | NA | NA | NA | NA | NA | NA | NA |
| Progovac 2021 | United States | 916 | No study name provided | 12-80 | NA | Suicidal ideation; diagnosis | Prevalence | 20.50% | NA | NA | NA | NA | NA | NA | NA |
| Rabasco 2020 | United States | 133 | No study name provided | 18+ | NA | Suicide attempt ever; Beck Scale for Suicidal Ideation (BSS) | Prevalence | 46.60% | NA | NA | NA | NA | NA | NA | NA |
| Rabasco 2021 | NA | 180 | No study name provided | 18+ | NA | Suicide attempt ever; self-report | Prevalence | 42.00% | NA | NA | NA | NA | NA | NA | NA |
| Radusky 2021b. | Argentina | 41 | COPA2 (Conexiones y Opciones Positivas en la Argentina 2) | 18+ | Individuals disengaged from HIV care | Suicide attempt ever; self-report | Prevalence | NA | NA | NA | 26.80% | NA | NA | NA | NA |
| Radusky 2021b. | Argentina | 41 | COPA2 (Conexiones y Opciones Positivas en la Argentina 2) | 18+ | Individuals disengaged from HIV care | Suicidal ideation P2W; BDI-II | Prevalence | NA | NA | NA | 22.00% | NA | NA | NA | NA |
| Real 2021 | Brazil | 34 | No study name provided | 18+ | NA | Suicidal ideation; Columbia Suicide Severity Rating Scale | Prevalence | NA | NA | NA | 58.10% | NA | NA | NA | NA |
| Real 2021 | Brazil | 34 | No study name provided | 18+ | NA | Suicide attempts; Columbia Suicide Severity Rating Scale | Prevalence | NA | NA | NA | 33.30% | NA | NA | NA | NA |
| Scheim 2020 | United States | 22286 | USTS | 18+ | NA | Suicidal ideation P12M; self-report | Prevalence | 48.80% | NA | NA | NA | NA | NA | NA | NA |
| Scheim 2020 | United States | 22286 | USTS | 18+ | NA | Suicide plan P12M; self-report | Prevalence | 24.10% | NA | NA | NA | NA | NA | NA | NA |
| Scheim 2020 | United States | 22286 | USTS | 18+ | NA | Suicide attempt P12M; self-report | Prevalence | 7.60% | NA | NA | NA | NA | NA | NA | NA |
| Schweizer 2020 | United States | 350 | THIS (Virginia Transgender Health Initiative Study) | 18+ | NA | Suicidal ideation; self-report | Prevalence | NA | NA | NA | 57.30% | 78.00% | NA | NA | NA |
| Seelman 2021 | United States | 7950 | USTS | 18+ | NA | Suicidal ideation ever; self-report | Prevalence | NA | NA | NA | NA | 84.80% | NA | NA | NA |
| Seelman 2021 | United States | 7950 | USTS | 18+ | NA | Suicide attempt ever; self-report | Prevalence | NA | NA | NA | NA | 44.30% | NA | NA | NA |
| Segev-Becker 2020 | Israel | 106 | No study name provided | 18-Apr | NA | Suicidal ideation; diagnosis | Prevalence | NA | 10.00% | 12.00% | NA | NA | NA | NA | NA |
| Segev-Becker 2020 | Israel | 106 | No study name provided | 18-Apr | NA | Suicide attempt; diagnosis | Prevalence | NA | 8.00% | 17.00% | NA | NA | NA | NA | NA |
| She 2020 | Canada | 92 | No study name provided | NA | NA | Suicide attempts, suicidal ideation or self-harm behaviors ever; diagnosis | Prevalence | Ever: 22.8%; Current: 10.9% | NA | NA | NA | NA | NA | NA | NA |
| She 2021a. | China | 235 | No study name provided | 18+ | TW SW | Suicidal ideation P12M; self-report | Prevalence | NA | NA | NA | Sample 1: 27.0%; Sample 2: 23.9% | NA | NA | NA | NA |
| Silva 2021 | Brazil | 189 | No study name provided | NA | NA | Suicidal ideation; self-report | Prevalence | 46.80% | NA | NA | 46.40% | 47.40% | NA | NA | NA |
| Silva 2021 | Brazil | 189 | No study name provided | NA | NA | Suicide attempt; self report | Prevalence | 31.60% | NA | NA | 31.20% | 32.10% | NA | NA | NA |
| Snooks 2021a. | United States; Australia; United Kingdom; Canada | 848 | No study name provided | 18+ | NA | At risk for suicide; Suicidal Behaviors Questionnaire-Revised | Prevalence | 73.00% | NA | NA | NA | NA | NA | NA | NA |
| Snooks 2021b. | United States; Australia; United Kingdom; Canada | 237 | No study name provided | 18-70 | NA | High risk of suicide; Suicidal Behaviors Questionnaire-Revised | Prevalence | 79.30% | NA | NA | NA | NA | NA | NA | NA |
| Sorbara 2020 | Canada | 184 | No study name provided | 15+ | Patients in puberty under 18 | Suicidal ideation current; diagnosis | Prevalence | 52.00% | NA | NA | NA | NA | NA | NA | NA |
| Sorbara 2020 | Canada | 184 | No study name provided | 15+ | Patients in puberty under 18 | Suicide attempt ever; diagnosis | Prevalence | 17.00% | NA | NA | NA | NA | NA | NA | NA |
| Srivastava 2021c. | United States | 261 | No study name provided | 24-Dec | Individuals accessing crisis services | Suicide attempt ever; self-report | Prevalence | TGD participants: 44.4% | NA | NA | NA | NA | NA | NA | 43.70% |
| Srivastava 2021c. | United States | 261 | No study name provided | 24-Dec | Individuals accessing crisis services | Suicidal ideation ever; self-report | Prevalence | TGD participants: 14.7% | NA | NA | NA | NA | NA | NA | 13.90% |
| Strang 2021b. | United States | 66 | No study name provided | 13-21 | Binary TGD individuals | Suicidal ideation; Achenbach System of Empirically Based Assessment | Prevalence | Youth with ASD: ~60.0%; allistic youth: ~35.0% | NA | NA | NA | NA | NA | NA | NA |
| Strauss 2020a. | Australia | 869 | Trans Pathways | 14-25 | NA | Suicidal thoughts ever; self-report | Prevalence | 82.40% | 77.30% | 84.10% | NA | NA | NA | NA | NA |
| Strauss 2020a. | Australia | 869 | Trans Pathways | 14-25 | NA | Suicide attempt ever; self-report | Prevalence | 48.10% | 43.10% | 49.80% | NA | NA | NA | NA | NA |
| Strauss 2020b. | Australia | 859 | Trans Pathways | 14-25 | NA | Suicidal thoughts P12M; self-report | Prevalence | 82.40% | NA | NA | NA | NA | NA | NA | NA |
| Strauss 2020b. | Australia | 859 | Trans Pathways | 14-25 | NA | Suicide attempt P12M; self-report | Prevalence | 48.10% | NA | NA | NA | NA | NA | NA | NA |
| Strauss 2021 | Australia | 859 | Trans Pathways | 14-25 | NA | Suicidal thoughts ever; self-report | Prevalence | With ASD: 87.7%; without ASD: 80.9% | NA | NA | NA | NA | NA | NA | NA |
| Strauss 2021 | Australia | 859 | Trans Pathways | 14-25 | NA | Suicide attempt ever; self-report | Prevalence | With ASD: 57.2%; without ASD: 45.6% | NA | NA | NA | NA | NA | NA | NA |
| Suarez 2021 | United States | 131 | No study name provided | 21-64 | NA | Suicide attempt ever; self-report | Prevalence | NA | NA | 17.2%-50.9% (across number of adverse childhood experiences) | NA | NA | NA | NA | NA |
| Tan 2021a. | Aotearoa/New Zealand | 49 | Counting Ourselves: Aotearoa New Zealand Trans and Non-Binary Health Survey | 14+ | Asian TGD individuals | Thought about attempting suicide P12M; self-report | Prevalence | 50.00% | NA | NA | NA | NA | NA | NA | NA |
| Tan 2021a. | Aotearoa/New Zealand | 49 | Counting Ourselves: Aotearoa New Zealand Trans and Non-Binary Health Survey | 14+ | Asian TGD individuals | Attempted suicide P12M; self-report | Prevalence | 11.10% | NA | NA | NA | NA | NA | NA | NA |
| Tan 2021b. | Aotearoa/New Zealand | 1178 | Counting Ourselves: the Aotearoa New Zealand Trans and Non-Binary Health Survey | 14+ | NA | Suicidal ideation P12M; self-report | Prevalence | 56.10% | NA | NA | NA | NA | NA | NA | NA |
| Tan 2021b. | Aotearoa/New Zealand | 1178 | Counting Ourselves: the Aotearoa New Zealand Trans and Non-Binary Health Survey | 14+ | NA | Suicide attempt P12M; self-report | Prevalence | 11.00% | NA | NA | NA | NA | NA | NA | NA |
| Taylor 2020 | Australia | 474 | Who I Am Study | 18+ | Bisexual Australians | Suicidal ideation ever and P24M; self-report | Prevalence | Ever: 91.6%; P24M: 54.6% | NA | NA | NA | NA | NA | NA | NA |
| Taylor 2020 | Australia | 474 | Who I Am Study | 18+ | Bisexual Australians | Attempted suicide ever and P24M; self-report | Prevalence | Ever: 48.8%; P24M: 12.7% | NA | NA | NA | NA | NA | NA | NA |
| Torres 2021 | Brazil | 86 | No study name provided | 18+ | NA | Suicidal thoughts during COVID-19 social distancing; self-report | Prevalence | 26.70% | NA | NA | NA | NA | NA | NA | NA |
| Treharne 2020 | New Zealand; Australia | 392 | No study name provided | 18+ | NA | Suicidal ideation ever and P1M; self-report and Suicidal Ideation Attributes Scale, respectively | Prevalence | Ever: 93.6%; P1M: 40.1% | NA | NA | NA | NA | NA | NA | NA |
| Treharne 2020 | New Zealand; Australia | 392 | No study name provided | 18+ | NA | Suicide attempt ever and P12M; self-report | Prevalence | Ever: 53.4%; P12M: 16.8% | NA | NA | NA | NA | NA | NA | NA |
| Turban 2020 | United States | 3494 | USTS | 18-36 | TGD individuals who wanted puberty blockers | Suicidal ideation ever and P12M; self-report | Prevalence | P12M: Received pubertal suppression: 50.6%; did not receive pubertal suppression: 64.8%  Ever:  Received pubertal suppression: 75.3%; did not receive pubertal suppression: 90.2% | NA | NA | NA | NA | NA | NA | NA |
| Turban 2020 | United States | 3494 | USTS | 18-36 | TGD individuals who wanted puberty blockers | Suicidal ideation with a plan P12M; self-report | Prevalence | Received pubertal suppression: 55.6%; did not receive pubertal suppression: 58.2% | NA | NA | NA | NA | NA | NA | NA |
| Turban 2020 | United States | 3494 | USTS | 18-36 | TGD individuals who wanted puberty blockers | Suicidal ideation with plan and attempt P12M and suicide attempt resulting in inpatient care P12M; self-report | Prevalence | Plan and attempt:  Received pubertal suppression: 24.4%; did not receive pubertal suppression: 21.5%  Attempt resulting in inpatient care:  Received pubertal suppression: 45.5%; did not receive pubertal suppression: 22.8% | NA | NA | NA | NA | NA | NA | NA |
| Turban 2020 | United States | 3494 | USTS | 18-36 | TGD individuals who wanted puberty blockers | Suicide attempt ever; self-report | Prevalence | Received pubertal suppression: 41.6%; did not receive pubertal suppression: 51.2% | NA | NA | NA | NA | NA | NA | NA |
| Turban 2021 | United States | 9711 | USTS | 18+ | TGD and gender diverse people who had undergone social transition and realized they were TGD during childhood | Suicidal ideation ever and P12M; self-report | Prevalence | Ever: 84.8%; P12M: 46.2% | NA | NA | NA | NA | NA | NA | NA |
| Turban 2021 | United States | 9711 | USTS | 18+ | TGD and gender diverse people who had undergone social transition and realized they were TGD during childhood | Suicidal ideation with plan P12M; self-report | Prevalence | 24.50% | NA | NA | NA | NA | NA | NA | NA |
| Turban 2021 | United States | 9711 | USTS | 18+ | TGD and gender diverse people who had undergone social transition and realized they were TGD during childhood | Suicide attempt ever; suicide attempt P12M; suicide attempt resulting in medical attention P12M; self-report | Prevalence | Suicide attempt ever: 47.4%; suicide attempt P12M: 8.2%; suicide attempt resulting in medical attention P12M: 3.9% | NA | NA | NA | NA | NA | NA | NA |
| Turner 2021 | Canada | NA | No study name provided | 18-Dec | NA | Suicidal ideation; self-report | Prevalence | 68.00% | NA | NA | NA | NA | NA | NA | NA |
| VanCauwenberg 2021 | Belgium | 177 | No study name provided | 18-Dec | Referred to pediatric gender clinic | Suicidal thoughts; item from Youth Self-Report | Prevalence | 40.40% | NA | NA | NA | NA | NA | NA | NA |
| VanCauwenberg 2021 | Belgium | 177 | No study name provided | 18-Dec | Referred to pediatric gender clinic | Suicide; not specified | Prevalence | 2.80% | NA | NA | NA | NA | NA | NA | NA |
| Vance 2021 | United States | 356 | 2015-2017 Biennial California Healthy Kids Survey | Grades 9 and 11 students | NA | Suicidal ideation P12M; self-report | Prevalence | Black and Latinx: 46.0%; White: 47.0% | NA | NA | NA | NA | NA | NA | NA |
| Wang 2020a. | China | 2111 | No study name provided | Grades 7-11 students | NA | Suicidal thoughts P1M; self-report | Prevalence | NA | NA | NA | 32.20% | 31.10% | 27.60% | 31.80% | Questioning AMAB: 23.7%; questioning AFAB: 31.8% |
| Wang 2020a. | China | 2111 | No study name provided | Grades 7-11 students | NA | Suicide plan P1M; self-report | Prevalence | NA | NA | NA | 12.70% | 8.20% | 14.80% | 11.60% | Questioning AMAB: 5.4%; questioning AFAB: 7.3% |
| Wang 2020a. | China | 2111 | No study name provided | Grades 7-11 students | NA | Suicide attempt ever; self-report | Prevalence | NA | NA | NA | 14.60% | 10.30% | 13.40% | 10.70% | Questioning AMAB: 9.3%; questioning AFAB: 7.0% |
| Wichaidit 2021 | Thailand | 755 | National School Survey on Alcohol Consumption, Substance Use and Other Health-Risk Behaviors | Years 7, 9 and 11 students | NA | Suicidal ideation P12M; self-report | Prevalence | NA | NA | NA | 11.50% | 16.00% | NA | NA | NA |
| Wichaidit 2021 | Thailand | 755 | National School Survey on Alcohol Consumption, Substance Use and Other Health-Risk Behaviors | Years 7, 9 and 11 students | NA | Suicide planning P12M; self-report | Prevalence | NA | NA | NA | 9.10% | 13.90% | NA | NA | NA |
| Wichaidit 2021 | Thailand | 755 | National School Survey on Alcohol Consumption, Substance Use and Other Health-Risk Behaviors | Years 7, 9 and 11 students | NA | Suicide attempt P12M; self-report | Prevalence | NA | NA | NA | 8.20% | 8.80% | NA | NA | NA |
| Wiepjes 2020 | Netherlands | 8263 | No study name provided | 4+ | NA | Death by suicide; National Civil Record Registry, hospital registration system, medical, and psychological files | Prevalence | 0.60% | NA | NA | 0.80% | 0.30% | NA | NA | NA |
| Wiepjes 2020 | Netherlands | 8263 | No study name provided | 4+ | NA | Death by suicide; National Civil Record Registry, hospital registration system, medical, and psychological files | Incidence | NA | NA | NA | 64.0 per 100,000PY | 29.0 per 100,000PY | NA | NA | NA |
| Witte 2020 | United States; United Kingdom | 32 | No study name provided | 18+ | LGBTQ+ veterinary professionals | Suicidal ideation; self-report | Prevalence | 50.00% | NA | NA | NA | NA | NA | NA | NA |
| Witte 2020 | United States; United Kingdom | 32 | No study name provided | 18+ | LGBTQ+ veterinary professionals | Suicide attempt ever; self-report | Prevalence | 15.60% | NA | NA | NA | NA | NA | NA | NA |
| Wolford-Clevenger 2021a | United States | 38 | No study name provided | 18+ | NA | Baseline suicidal ideation P1W and suicidal ideation at least once P30D; Hopelessness Depression Symptom Questionnaire-Suicidality Sub-scale and Paykel Suicide Scale, respectively | Prevalence | Baseline P1W: 59.5%; P30D: 71.0% | NA | NA | NA | NA | NA | NA | NA |
| Wolford-Clevenger 2021b | United States | 38 | No study name provided | 18+ | NA | Suicidal ideation; Hopelessness Depression Symptom Questionnaire-Suicidality Sub-scale | Prevalence | 59.50% | NA | NA | NA | NA | NA | NA | NA |
| Yan 2021 | China | 222 | No study name provided | 18+ | NA | Attempted suicide ever; self-report | Prevalence | NA | NA | NA | 25.70% | NA | NA | NA | NA |
| Yockey 2020 | United States | 27715 | USTS | 18+ | NA | Suicidal ideation P12M; self-report | Prevalence | NA | NA | NA | 48.00% | 43.10% | NA | NA | Non-binary: 53.0%; part time one gender, part time another: 54.2% |
| Zwickl 2021b. | Australia | 928 | No study name provided | 18+ | NA | Suicide attempts ever; self-report | Prevalence | 43.00% | NA | NA | NA | NA | NA | NA | NA |

ADD/ADHD = attention deficit (hyperactivity) disorder; AFAB = assigned female at birth; AMAB = assigned male at birth; ART = antiretroviral treatment; AUD = alcohol use disorder; AUDIT = Alcohol Use Disorder Identification Tool; BDI = Beck Depression Inventory; BMI = body mass index; CAGE = Cut, Annoyed, Guilty, and Eye substance abuse screening tool; CESD = Center for Epidemiologic Studies Depression screener; COPD = chronic obstructive pulmonary disease; CVD = cardiovascular disease; DAST = Drug Abuse Screening Test; GAD = generalized anxiety disorder; GAHT = gender-affirming hormone therapy; GD = gender dysphoria; HIV = human immunodeficiency virus; HPV = human papilloma virus; MSM = men who have sex with men; NA = not applicable, NSSI = non-suicidal self-injury; OCD = obsessive compulsive disorder; P#M = past # of months; P#W = past # of weeks; PHQ = Patient Health Questionnaire; PLHIV = people living with HIV; PTSD = post-traumatic stress disorder, SGM = sexual and gender minority; STI = sexually transmitted infection; SUD = substance use disorder; SW = sex worker; TGD = transgender and gender diverse; TM = transgender men or transmasculine; TW = transgender women or transfeminine; USTS = United States Transgender Survey; VA = U.S. Veterans Administration
