# Supplementary material for: Health status of transgender people globally: A systematic review of research on disease burden and correlates
Source: PLoS One. 2024 Mar 11;19(3):e0299373. doi: 10.1371/journal.pone.0299373 (PMC10927095; doi:10.1371/journal.pone.0299373)
Supplement: S4 Table — (DOCX) [file pone.0299373.s004.docx]

**Supplementary Table S4. Risk and protective factors (n=136 studies)**

| **Condition** | **Community, social support, resilience** | **Demographics and socioeconomic position** | **Gender affirmation and dysphoria/ incongruence** | **Health behaviors, status, and health care use** | **Minority stressors and violence** | **Policies** |
| --- | --- | --- | --- | --- | --- | --- |
| **Chronic conditions** | | | | | | |
| Cardiovascular/ cerebrovascular |  | Higher income (+); Black race (-); Older age (-); Black race (-) | Feminizing hormone therapy (+); Female sex (-);  Masculinizing hormone therapy (-);  Feminizing hormone therapy (-) | All-cause mortality (-);  Chronic disease (-);  Geographic-related Hospital factors related to geography, increased length of stay and cost (-);  Psychological distress (-) |  |  |
| Cancer |  | Older age (-);  Black race/ethnicity (-) |  |  |  |  |
| Metabolic/endocrine (e.g., diabetes) |  | Black or Latinx identity (-);  Older age (-);  Black race (-) |  |  | Adverse childhood experiences (-) |  |
| Respiratory |  | Older age (-) |  |  |  |  |
| Other chronic conditions |  | Being unmarried (-);  Older age (-);  Unemployment (-) | Feminizing hormone therapy (-) | Higher BMI (-); Eating disorder (-) |  |  |
| **Mental health and substance use disorders** | | | | | | |
| Depression | Childhood physical neglect (+);  Left family because of sexuality (+);  Social support (+); Experience of loss (-);  Isolated from other trans people (-);  Issues at school (-);  Lack of family support (-);  Low social support (-);  Lower resilience (-);  Lower social support (-);  Poor social relationship (-) | Lower educational attainment (+);  Older age (+);  Racial/ethnic minority (+); AFAB (-);  Asexuality (-);  COVID-19-related socioeconomic losses (-);  Disability (-);  Employment issues (-);  European, American, or Eastern Mediterranean region (-);  Ever married (-);  Financial strain (-);  Gender minority identity (-);  Non-binary, trans woman or man gender identity (-);  Higher education (-);  Housing instability (-);  Housing issues (-);  Increased work hours (-);  Lower education or SES (-);  Lower educational attainment (-);  Lower income (-);  Married not living with spouse (-);  Minority sexual orientation (-);  More COVID-19 related work activities (-);  Non-binary identity (-);  Older age (-);  Racial/ethnic minority (-);  Sex work(-);  Trans men and non-binary identities (-);  Trans woman identity (-);  Trans woman or non-binary identity (-);  Transfeminine identity (-);  Younger age (-) | Legal name change (+);  More time on hormones (+);  Puberty suppression (+);  Visual conformity with gender (+); Body dysphoria (-);  Compulsory gender conformity (-);  COVID-19-related reductions in access to gender-affirming services (-);  Denial of health care (-);  Gender non-affirmation from partners (-);  Gender-affirming surgery (-);  Hormone therapy (-);  Loss of gender affirmation access (-);  Loss of gender expression (-);  Negative health care provider experience (-);  Negative self-image (-);  Stigma (-);  Trans-negative media messaging (-) | Alcohol use (-);  Anxiety (-);  ASD (-);  Condomless anal sex (-);  High sexual relationship (-);  Isolated from services (-);  Lower ART adherence (-);  Lower quality of life (-);  Paranoia (-);  Poor physical health-related quality of life (-);  Poor physical health (-);  Psychopathic deviate personality (-);  Substance use (-);  Suicidality (-);  Suicide attempt (-) | Adverse childhood experiences (-);  Bathroom discrimination (-);  Bullying (-);  Childhood abuse (-);  Childhood emotional abuse (-);  Childhood physical abuse (-);  Childhood sexual abuse (-);  Discrimination (-);  Familial sexual abuse (-);  Family- or acquaintance-perpetrated violence (-);  Gender identity stigma (-);  Internalized transphobia (-);  Interpersonal violence (-);  Knowledge of anti-trans state legislation (-);  Mistreatment (-);  Peer rejection (-);  Physical violence (-);  Stigma (-);  Unsafe in household (-);  Violence (-) | Concern over loss of rights (-);  COVID-19 pandemic restriction (-) |
| Suicidality | Neighborhood belonging (+);  Primary social ties (+);  School belonging (+);  Social support (+);  Trans community belonging (+); Experience of loss (-);  Family emotional neglect (-);  Issues at school (-);  Lack of family support (-);  Peer rejection (-);  Perceived burdensomeness (-);  Perceived lack of regional area safety (-)  Unsupportive mother (-) | Asian identity (+);  Higher income (+);  Non-precarious immigration status (+);  Older age (+); AFAB (-);  AMAB (-);  Disability (-);  Employment issues (-);  Female gender identity (-);  Financial strain (-);  Gender minority (-);  Higher education attainment (-);  Homelessness (-);  Housing issues (-);  Increased work hours (-);  Lack of school enrollment (-);  Rural location (-);  Lower income (-);  Male gender (-);  Medicaid eligibility (-);  Minority racial identity (-);  Minority sexual orientation (-);  More COVID-19 related work activities (-);  Non-binary identity (-);  Non-binary/gender queer identity (-);  Non-questioning gender identity (-);  Older age (-);  Part time one gender/part time another (-);  Poverty (-);  White race (-);  Racial/ethnic minority (-);  Residing with others (-);  Sexual minority identity (-); Bisexual, pansexual, or queer sexual orientation (-);  Trans woman identity (-);  Unemployment (-);  White or American Indian race/ethnicity (-);  Woman gender identity (-);  Younger age (-); | Gender affirmation (+);  Gender-concordant ID (+);  Presumed male-at-birth (+); Presumed male-at-birth (+);  Age of social transition (-);  Bathroom discrimination (-);  Body dysphoria (-);  Desire for gender-affirming surgery (-);  Desire to change physical appearance (-);  Gender-affirming surgery cancelled or postponed (-);  Lack of family support (-);  Loss of gender affirmation access (-);  Lower hormone access (-);  No hormone therapy (-); | Greater nightmare severity (+);  Acquired capability (-);  Alcohol use (-);  Anxiety (-);  ASD (-);  Behavioral health conditions (-);  Chronic disease (-);  Clinic referral in adulthood (-);  Depression (-);  Depressive symptoms (-);  Distress (-);  Greater nightmare frequency (-);  Health care avoidance (-);  Isolated from services (-);  Less dispositional optimism (-);  Loneliness (-);  No mental health care (-);  No mental health provider (-);  Poor health care treatment (-);  Poor health status (-);  Psychological distress (-);  PTSD (-);  Reduced cognition (-);  Smoking (-);  Substance use (-) | Substance use (-);  Abuse/violence (-);  Adverse childhood experiences (-);  Bathroom discrimination (-);  Bullying (-);  Discrimination (-);  Enacted stigma (-);  Extrafamilial physical abuse (-);  Familial physical abuse (-);  Forced sex (-);  Gun violence (-);  Harassment (-);  Health care mistreatment (-);  Institutional discrimination (-);  Internalized homophobia (-);  Internalized stigma (-);  Internalized transphobia (-);  Interpersonal violence (-);  IPV (-);  Microaggressions (-);  Peer rejection (-);  Physical assault (-);  Physical violence (-);  Sexual Abuse (-);  Sexual violence (-);  Stigma (-);  Stress (-);  Trans-specific negative experiences with doctor (-);  Unsafe in household (-);  Verbal abuse (-);  Verbal harassment or assault in healthcare setting (-);  Violence (-) | Nondiscrimination policies (+);  Protective state policies (+) |
| Anxiety | Experience of loss (-);  Isolated from other trans people (-);  Issues at school (-);  Lack of family support (-) | COVID-19-related socioeconomic losses (-);  Disability (-);  Employment issues (-);  European, American, or Eastern Mediterranean region (-);  Housing issues (-);  Increased work hours (-);  Lower education or SES (-);  Lower educational attainment (-);  More COVID-19 related work activities (-);  Non-binary AFAB (-);  Non-binary identity (-);  Older age (-);  Trans men and non-binary identities (-);  Transfeminine identity (-);  Younger age (-) | Gender marker change (+);  Legal name change (+);  Longer time since last gender affirming surgery (+);  Social and healthcare-related gender affirmation (+);  Visual conformity with gender (+); Conversion therapy exposure (-);  COVID-19-related reductions in access to gender-affirming services (-);  Denial of health care (-);  Gender non-affirmation from partners (-);  Lack of gender affirmation (-);  Loss of gender affirmation access (-);  Loss of gender expression (-);  Trans-negative media messaging (-) | Alcohol use (-);  ASD (-);  Depression (-);  Isolated from services (-);  Suicidality (-) | Adulthood abuse (-);  Bullying (-);  Discrimination (-);  Extrafamilial physical abuse (-);  Familial sexual abuse (-);  Interpersonal violence (-);  Peer rejection (-);  Stigma (-);  Violence (-) | Concern over loss of rights (-);  COVID-19 pandemic restriction (-) |
| Psychological distress | Neighborhood belongingness (+);  Participation in LGBTI community events (+);  Primary social ties (+);  Transgender community belongingness (+);  Lower social support (-);  Peer rejection (-) | Bisexual/unsure sexual orientation (-);  Financial insecurity (-);  Rural location (-);  West, Midwest region (-);  Lower educational attainment (-);  Lower income (-);  Minority sexual orientation (-);  Non-binary identity (-);  Older age (-);  Bi, pan, queer sexual orientation (-);  Younger age (-) | Gender marker change (+);  Gender-concordant ID (+);  Legal name change (+);  Trans-negative media messaging (-) | Alcohol use (+);  Condomless receptive anal intercourse (-);  Lack of premeditation (-);  No self-harm urgency (-);  Prescription drug misuse (-);  E-cigarettes/vaping (-) | Verbal abuse for being trans (+);  Abuse/violence (-);  Adulthood abuse (-);  Enacted stigma (-);  Felt sexuality is bad/wrong (-);  Forced sex (-);  Health care stigma (-);  Interpersonal violence (-);  IPV (-);  Stigma (-);  Trans-specific negative experiences with doctor (-);  Verbal abuse (-);  Verbal harassment or assault in health care setting (-) | Concern over loss of rights (-) |
| Other mental health condition | Neighborhood belonging (+);  Primary social ties (+);  Social support (+);  Transgender community belonging (+);  Experience of loss (-);  Isolated from other trans people (-);  Issues at school (-);  Lack of family support (-);  Low social support (-);  Lower resilience (-);  No religious preference (-) | "Other" race/ethnicity (+);  AFAB (-);  Disability (-);  Employment issues (-);  Female gender identity (-);  Financial insecurity (-);  Housing issues (-);  Increased work hours (-);  Lower educational attainment (-);  More COVID-19 related work activities (-);  Non-binary identity (-);  Older age (-);  Trans girl identity (-);  Trans woman identity (-);  Transmasculine identity (-) | Gender affirmation (+);  Hormones and/or surgery (+);  Longer time since last gender affirming surgery (+);  Negative self-image (-);  Trans-negative media messaging (-) | Alcohol use (-);  ASD (-);  BMI (-);  Distress (-);  Hospitalization (-);  Isolated from services (-);  Lower ART adherence (-);  Mood disorder history (-);  Suicidal Ideation (-) | Abuse (-);  Adulthood abuse (-);  Adverse childhood experiences (-);  Bullying (-);  Discrimination (-);  Enacted stigma (-);  Extrafamilial physical abuse (-);  Familial physical abuse (-);  Familial sexual abuse (-);  Peer rejection (-);  Stigma (-) |  |
| Alcohol use disorder | Family functioning (+); Social support (+) | Employed (+); AFAB (-); AMAB (-); Non-binary gender identity (-)  Older age (-); Transfeminine identity (-) | Gender dysphoria (-); Identity concealment (-) | Depression (+); Alcohol use (-); Anal sex with men for money/gifts (-); Lower condom use (-); No self-harm urgency (-); substance use (-) | Childhood verbal abuse by family (-); Discrimination (-); Dropped out of school due to trans status (-); Gender identity stigma (-); Higher gender minority stress (-); Internalized transphobia (-); Interpersonal violence (-); IPV (-); Stigma (-); Stranger-perpetuated violence (-); Violence (-) |  |
| Nicotine/ tobacco use disorder | Resilience (+) | AMAB (-);  Transfeminine identity (-) | Gender-related pride (+) |  |  |  |
| Drug use disorder | Family functioning (+);  Resilience (+);  Social Support (+);  Lower resilience (-) | Race (Asian Pacific Islander) (+);  Racial/ethnic minority (+);  Transmasculine identity (+);  Gender identity (nonbinary, trans masculine)) (-);  Housing issues (-);  Lower educational attainment (-);  Non-binary AMAB (-);  Non-binary identity (-);  Poverty (-);  Race/Ethnicity (Biracial, Multiracial) (-);  Sexual minority identity (-);  Trans girl identity (-);  Transfeminine gender identity (-) |  | ASD (-);  Depression (-);  Lack of health insurance (-);  Poorer health status (-);  Psychological distress (-);  PTSD (-) | Discrimination (-);  Gender identity stigma (-);  Gender-based discrimination (-);  Higher gender minority stress levels (-);  IPV (-);  Transgender-related discrimination (-) |  |
| **Infectious diseases** | | | | | | |
| HIV |  | AMAB (-);  Black or Latina identity (-);  Black race (-);  Food insecurity (-);  Homelessness (-);  Low income (-);  Lower educational attainment (-);  Male sex on ID (-);  Neighborhood (-);  Neighborhood location (-);  Older age (-);  Poverty (-);  Public insurance (-);  Race/ethnicity (Black) (-);  Race/ethnicity (Latinx, other race/ethnicity) (-);  Sex work (-);  Travesti identity (-);  Unemployment (-);  Younger age (-) |  | Mental health symptoms (+);  Regular male partner (+);  Anal intercourse (-);  First time HIV testing (-);  History of previous STIs (-);  Incarceration (-);  Inconsistent condom use (-);  Insurance (Medicaid) (-);  Lack of health insurance (-);  STI (-);  Substance use (-) |  |  |
| Other STIs |  | Education (+);  Lower educational attainment (-);  Lower neighborhood income (-);  Older age (-);  Travesti identity (-);  Younger age (-) |  | PrEP use (+);  Anorectal chlamydia (-);  Higher number of insertive anal sex partners (-);  Higher number of receptive anal sex partners (-);  HIV (-);  Illicit substance use (-);  Inconsistent condom use (-);  Previous STI (-);  Receptive anal sex (-);  Receptive penile vaginal sex (-);  STI (-) | Arrest history (-) |  |
| Other infectious disease |  | Older age (-) |  |  |  |  |

AFAB = assigned female at birth; AMAB = assigned male at birth; ART **=** antiretroviral treatment; ASD = autism spectrum disorder; BMI = body mass index; HIV = human immunodeficiency virus; ID = identity documents; IPV = intimate partner violence; LGBTI = lesbian, gay, bisexual, transgender, intersex; PrEP = pre-exposure prophylaxis; PTSD = post-traumatic stress disorder; SES = socioeconomic status; STI = sexually transmitted infection.
