# Supplementary material for: Health status of transgender people globally: A systematic review of research on disease burden and correlates
Source: PLoS One. 2024 Mar 11;19(3):e0299373. doi: 10.1371/journal.pone.0299373 (PMC10927095; doi:10.1371/journal.pone.0299373)
Supplement: S1 Appendix — (DOCX) [file pone.0299373.s005.docx]

**All Articles Included in Review**

1. Abramovich A, de Oliveira C, Kiran T, Iwajomo T, Ross LE, Kurdyak P. Assessment of Health Conditions and Health Service Use Among Transgender Patients in Canada. JAMA Network Open 2020; 3(8): e2015036.
2. Achille C, Taggart T, Eaton NR, et al. Longitudinal impact of gender-affirming endocrine intervention on the mental health and well-being of transgender youths: preliminary results. International Journal of Pediatric Endocrinology 2020; 2020: 8.
3. Aguilar G, Samudio T, Lopez G, et al. High HIV prevalence among transgender women in Paraguay. International Journal of STD & AIDS 2020; 31(13): 1308-14.
4. Aldridge Z, Patel S, Guo B, et al. Long-term effect of gender-affirming hormone treatment on depression and anxiety symptoms in transgender people: A prospective cohort study. Andrology 2020; 10: 10.
5. Allen SD, Tollit MA, McDougall R, Eade D, Hoq M, Pang KC. A Waitlist Intervention for Transgender Young People and Psychosocial Outcomes. Pediatrics 2021; 148(2): 1-10.
6. Alvarado B, Mueses HF, Galindo J, Martinez-Cajas JL. Application of the "syndemics" theory to explain unprotected sex and transactional sex: A crosssectional study in men who have sex with men (MSM), transgender women, and non-MSM in Colombia. Biomedica 2020; 40(2): 391-403.
7. Anderssen N, Sivertsen B, Lonning KJ, Malterud K. Life satisfaction and mental health among transgender students in Norway. BMC Public Health 2020; 20(1): 138.
8. Andrew SJ, Cogan CM, Scholl JA, Davis JL. Nightmares as a unique predictor of suicide risk in a transgender and gender diverse sample. Dreaming 2020; 30(4): 329-37.
9. Andrew Yockey R, King KA, Vidourek RA. Correlates to Lifetime Suicide Attempts, Thoughts, and Planning Behaviors Among African American Transgender Individuals. Journal of Primary Prevention 2020; 41(6): 487-501.
10. Angoff HD, McGraw JS, Docherty M. Intersecting Identities and Nonsuicidal Self-Injury among Youth. Identity-an International Journal of Theory and Research 2021; 21(2): 98-114.
11. Antebi-Gruszka N, Talan AJ, Reisner SL, Rendina HJ. Sociodemographic and behavioural factors associated with testing for HIV and STIs in a US nationwide sample of transgender men who have sex with men. Sexually Transmitted Infections 2020; 96(6): 422-7.
12. Antwi-Amoabeng D, Doshi R, Adalja D, et al. Burden of arrythmias in transgender patients hospitalized for gender-affirming surgeries. Journal of Arrhythmia 2020; 36(4): 797-800.
13. Anzani A, Panfilis C, Scandurra C, Prunas A. Personality Disorders and Personality Profiles in a Sample of Transgender Individuals Requesting Gender-Affirming Treatments. International Journal of Environmental Research & Public Health [Electronic Resource] 2020; 17(5): 27.
14. Arikawa AY, Ross J, Wright L, Elmore M, Gonzalez AM, Wallace TC. Results of an Online Survey about Food Insecurity and Eating Disorder Behaviors Administered to a Volunteer Sample of Self-Described LGBTQ+ Young Adults Aged 18 to 35 Years. Journal of the Academy of Nutrition & Dietetics 2021; 121(7): 1231-41.
15. Aristegui I, Radusky PD, Zalazar V, et al. Correlates of depressive symptoms in transgender women initiating HIV treatment in Argentina. Journal of Gay & Lesbian Mental Health 2021; 25(2): 208-25.
16. Aspia NK, Djuwita R. The relationship between selling sex and HIV status among transgender in indonesia (Analysis of integreted biological and behavioral survey data 2015). Indian Journal of Public Health Research and Development 2020; 11(6): 1416-22.
17. Atteberry-Ash B, Kattari SK, Harner V, et al. Differential Experiences of Mental Health among Transgender and Gender-Diverse Youth in Colorado. Behavioral sciences (Basel, Switzerland) 2021; 11(4).
18. Austin A, Craig SL, D'Souza S, McInroy LB. Suicidality Among Transgender Youth: Elucidating the Role of Interpersonal Risk Factors. Journal of Interpersonal Violence 2020: 886260520915554.
19. Balcerek MI, Nolan BJ, Brownhill A, et al. Feminizing Hormone Therapy Prescription Patterns and Cardiovascular Risk Factors in Aging Transgender Individuals in Australia. Frontiers in Endocrinology 2021; 12.
20. Banks K, Kyinn M, Leemaqz SY, Sarkodie E, Goldstein D, Irwig MS. Blood Pressure Effects of Gender-Affirming Hormone Therapy in Transgender and Gender-Diverse Adults. Hypertension 2021: HYPERTENSIONAHA12016839.
21. Barger BT, Obedin-Maliver J, Capriotti MR, Lunn MR, Flentje A. Characterization of substance use among underrepresented sexual and gender minority participants in The Population Research in Identity and Disparities for Equality (PRIDE) Study. Substance Abuse 2021; 42(1): 104-15.
22. Batchelder AW, Stanton AM, Kirakosian N, et al. Mental Health and Substance Use Diagnoses and Treatment Disparities by Sexual Orientation and Gender in a Community Health Center Sample. LGBT Health 2021; 8(4): 290-9.
23. Batista RL, Verduguez EDR, Inacio M, et al. Impact of schooling in the HIV/AIDS prevalence among Brazilian transgender women. Archives of Endocrinology & Metabolism 2020; 64(4): 369-73.
24. Becerra MB, Rodriquez EJ, Avina RM, Becerra BJ. Experiences of violence and mental health outcomes among Asian American transgender adults in the United States. PLoS ONE [Electronic Resource] 2021; 16(3): e0247812.
25. Biedermann SV, Asmuth J, Schröder J, Briken P, Auer MK, Fuss J. Childhood adversities are common among trans people and associated with adult depression and suicidality. Journal of Psychiatric Research 2021; 141: 318-24.
26. Blosnich JR, Boyer TL, Brown GR, Kauth MR, Shipherd JC. Differences in Methods of Suicide Death Among Transgender and Nontransgender Patients in the Veterans Health Administration, 1999-2016. Medical Care 2021; 59: S31-S5.
27. Boehmer U, Gereige J, Winter M, Ozonoff A, Scout N. Transgender individuals' cancer survivorship: Results of a cross-sectional study. Cancer 2020; 126(12): 2829-36.
28. Bohlmann ML, Dolan AJ, Jones NC, Knox BD. Negative Healthcare Experiences, Healthcare-Seeking Behavior, and Mental Health in Tampa Bay Area Transgender and Gender-Nonconforming Individuals. Southern medical journal 2021; 114(6): 361-7.
29. Boskey ER, Jolly D, Tabaac AR, Ganor O. Behavioral Health Concerns and Eligibility Factors Among Adolescents and Young Adults Seeking Gender-Affirming Masculinizing Top Surgery. Lgbt Health 2020; 7(4): 182-9.
30. Boyer TL, Youk AO, Haas AP, et al. Suicide, Homicide, and All-Cause Mortality Among Transgender and Cisgender Patients in the Veterans Health Administration. Lgbt Health 2021; 8(3): 173-80.
31. Branstrom R, Pachankis JE. Reduction in Mental Health Treatment Utilization Among Transgender Individuals After Gender-Affirming Surgeries: A Total Population Study. American Journal of Psychiatry 2020; 177(8): 727-34.
32. Bretherton I, Thrower E, Zwickl S, et al. The Health and Well-Being of Transgender Australians: A National Community Survey. Lgbt Health 2021; 8(1): 42-9.
33. Brown MJ, Patterson R. Subjective Cognitive Decline Among Sexual and Gender Minorities: Results from a U.S. Population-Based Sample. Journal of Alzheimer's Disease 2020; 73(2): 477-87.
34. Bryant-Genevier J, Rao CY, Lopes-Cardozo B, et al. Symptoms of Depression, Anxiety, Post-Traumatic Stress Disorder, and Suicidal Ideation Among State, Tribal, Local, and Territorial Public Health Workers During the COVID-19 Pandemic - United States, March-April 2021. MMWR Morbidity and mortality weekly report 2021; 70(26): 947-52.
35. Burns ZT, Bitterman DS, Perni S, et al. Clinical Characteristics, Experiences, and Outcomes of Transgender Patients With Cancer. JAMA Oncology 2021; 7(1): e205671.
36. Busby DR, Horwitz AG, Zheng K, et al. Suicide risk among gender and sexual minority college students: The roles of victimization, discrimination, connectedness, and identity affirmation. Journal of Psychiatric Research 2020; 121: 182-8.
37. Buspavanich P, Lech S, Lermer E, et al. Well-being during COVID-19 pandemic: A comparison of individuals with minoritized sexual and gender identities and cis-heterosexual individuals. PLoS ONE 2021; 16(6): e0252356.
38. Cantu AL, Moyer DN, Connelly KJ, Holley AL. Changes in Anxiety and Depression from Intake to First Follow-Up Among Transgender Youth in a Pediatric Endocrinology Clinic. Transgender Health 2020; 5(3): 196-200.
39. Cardona-Arias JA, Catano Correa JC, Higuita-Gutierrez LF. HIV Prevalence and Associated Factors in Key Groups and General Population Treated at a Health Care Institution in Colombia in 2019: A Cross-Sectional Study. HIV/AIDS Research and Palliative Care 2020; 12: 381-91.
40. Cerel J, Tucker RR, Aboussouan A, Snow A. Suicide exposure in transgender and gender diverse adults. Journal of Affective Disorders 2021; 278: 165-71.
41. Chen Y, Chen S, Arayasirikul S, et al. A cross-sectional study of mental health, suicidal ideation and suicide attempt among transgender women in Jiangsu province, China. Journal of Affective Disorders 2020; 277: 869-74.
42. Cheung AS, Leemaqz SY, Wong JWP, et al. Non-Binary and Binary Gender Identity in Australian Trans and Gender Diverse Individuals. Archives of Sexual Behavior 2020; 49(7): 2673-81.
43. Chumakov EM, Ashenbrenner YV, Petrova NN, Zastrozhin MS, Azarova LA, Limankin OV. Anxiety and Depression Among Transgender People: Findings from a Cross-Sectional Online Survey in Russia. LGBT Health 2021.
44. Cicero EC, Reisner SL, Merwin EI, Humphreys JC, Silva SG. The health status of transgender and gender nonbinary adults in the United States. PLoS ONE [Electronic Resource] 2020; 15(2): e0228765.
45. Clark J, Reisner S, Perez-Brumer A, et al. TransPrEP: Results from the Pilot Study of a Social Network-Based Intervention to Support PrEP Adherence Among Transgender Women in Lima, Peru. AIDS & Behavior 2021; 25(6): 1873-83.
46. Coakley KE, Le H, Silva SR, Wilks A. Anxiety is associated with appetitive traits in university students during the COVID-19 pandemic. Nutrition journal 2021; 20(1): 45.
47. Cocchetti C, Castellini G, Iacuaniello D, et al. Does Gender-Affirming Hormonal Treatment Affect 30-Year Cardiovascular Risk in Transgender Persons? A Two-Year Prospective European Study (ENIGI). Journal of Sexual Medicine 2021; 18: 18.
48. Cramer RJ, Langhinrichsen-Rohling J, Kaniuka AR, et al. Preferences in Information Processing, Marginalized Identity, and Non-Monogamy: Understanding Factors in Suicide-Related Behavior among Members of the Alternative Sexuality Community. International Journal of Environmental Research & Public Health [Electronic Resource] 2020; 17(9): 06.
49. Crowell TA, Lawlor J, Lombardi K, et al. Anorectal and Urogenital Mycoplasma genitalium in Nigerian Men Who Have Sex With Men and Transgender Women: Prevalence, Incidence, and Association With HIV. Sexually Transmitted Diseases 2020; 47(3): 202-6.
50. Davy-Mendez T, Napravnik S, Eron JJ, et al. Racial, ethnic, and gender disparities in hospitalizations among persons with HIV in the United States and Canada, 2005-2015. Aids 2021; 11: 11.
51. de Blok CJ, Dijkman BA, Wiepjes CM, et al. Frequency and outcomes of benign breast biopsies in trans women: A nationwide cohort study. Breast 2021; 57: 118-22.
52. de Nie I, de Blok CJM, van der Sluis TM, et al. Prostate Cancer Incidence under Androgen Deprivation: Nationwide Cohort Study in Trans Women Receiving Hormone Treatment. Journal of Clinical Endocrinology & Metabolism 2020; 105(9): 01.
53. de Nie II, Wiepjes CC, de Blok CC, et al. Incidence of testicular cancer in trans women using gender-affirming hormonal treatment: a nationwide cohort study. BJU international 2021.
54. Del Rio-Gonzalez AM, Zea MC, Florez-Donado J, et al. Sexual Orientation and Gender Identity Change Efforts and Suicide Morbidity Among Sexual and Gender Minority Adults in Colombia. LGBT Health 2021.
55. Denby KJ, Cho L, Toljan K, Patil M, Ferrando CA. Assessment of cardiovascular risk in transgender patients presenting for gender-affirming care. The American Journal of Medicine 2021; 134(8): 1002-8.
56. Deutsch MB, Reisner SL, Peitzmeier S, Potter J, Pardee D, Hughto JMW. Recent Penile Sexual Contact Is Associated With an Increased Odds of High-Risk Cervical Human Papillomavirus Infection in Transgender Men. Sexually Transmitted Diseases 2020; 47(1): 48-53.
57. Dinger MK, Brittain DR, Patten L, et al. Gender Identity and Health-related Outcomes in a National Sample of College Students. American Journal of Health Education 2020; 51(6): 383-94.
58. Drescher CF, Griffin JA, Casanova T, et al. Associations of physical and sexual violence victimisation, homelessness, and perceptions of safety with suicidality in a community sample of transgender individuals. Psychology & Sexuality 2021; 12(1-2): 52-63.
59. Druckler S, van Rooijen MS, de Vries HJC. Substance Use and Sexual Risk Behavior Among Male and Transgender Women Sex Workers at the Prostitution Outreach Center in Amsterdam, the Netherlands. Sexually Transmitted Diseases 2020; 47(2): 114-21.
60. Du Bois SN, Guy AA, Legate N, Kendall AD. Examining partnership-health associations among transgender individuals using Behavioral Risk Factor Surveillance System (BRFSS) data. Psychology of Sexual Orientation and Gender Diversity 2020.
61. Eastwood EA, Nace AJ, Hirshfield S, Birnbaum JM. Young Transgender Women of Color: Homelessness, Poverty, Childhood Sexual Abuse and Implications for HIV Care. AIDS & Behavior 2021; 25: 96-106.
62. Fan X, Lau JTF, Cai Y, et al. Prevalence and associated factors of sexualized drug use in sex work among transgender women sex workers in China. AIDS Care 2021; 33(8): 1098-106.
63. Fearon E, Tenza S, Mokoena C, et al. HIV testing, care and viral suppression among men who have sex with men and transgender individuals in Johannesburg, South Africa. PLoS ONE [Electronic Resource] 2020; 15(6): e0234384.
64. Fein LA, Cunha IR, Wong A, Schlumbrecht MP, Duthely LM, Potter JE. Low Perceived Anal Cancer Risk and Screening Utilization Among High-Risk Transgender Men and Women Living in an HIV / STI Epicenter. AIDS & Behavior 2021; 25(7): 2210-8.
65. Felner JK, Haley SJ, Jun HJ, Wisdom JP, Katuska L, Corliss HL. Sexual orientation and gender identity disparities in co-occurring depressive symptoms and probable substance use disorders in a national cohort of young adults. Addictive Behaviors 2021; 117: 106817.
66. Ferlatte O, Panwala V, Rich AJ, et al. Identifying Health Differences Between Transgender and Cisgender Gay, Bisexual and Other Men Who Have Sex With Men Using a Community-Based Approach. Journal of Sex Research 2020; 57(8): 1005-13.
67. Ferlatte O, Salway T, Rice SM, Oliffe JL, Knight R, Ogrodniczuk JS. Inequities in depression within a population of sexual and gender minorities. Journal of Mental Health 2020; 29(5): 573-80.
68. Fish JN, Bishop MD, Russell ST. Developmental Differences in Sexual Orientation and Gender Identity-Related Substance Use Disparities: Findings From Population-Based Data. Journal of Adolescent Health 2021; 18: 18.
69. Fish JN, Bishop MD, Russell ST. Developmental Differences in Sexual Orientation and Gender Identity–Related Substance Use Disparities: Findings From Population-Based Data. Journal of Adolescent Health 2021; 68(6): 1162-9.
70. Flatt JD, Cicero EC, Lambrou NH, et al. Subjective cognitive decline higher among sexual and gender minorities in the United States, 2015-2018. Alzheimer's & dementia (New York, N Y) 2021; 7(1): e12197.
71. Flentje A, Barger BT, Capriotti MR, et al. Screening gender minority people for harmful alcohol use. PLoS ONE [Electronic Resource] 2020; 15(4): e0231022.
72. Fontanari AMV, Vilanova F, Schneider MA, et al. Gender Affirmation Is Associated with Transgender and Gender Nonbinary Youth Mental Health Improvement. Lgbt Health 2020; 7(5): 237-47.
73. Frola CE, Zalazar V, Cardozo N, et al. Home-based HIV testing: Using different strategies among transgender women in Argentina. PLoS ONE [Electronic Resource] 2020; 15(3): e0230429.
74. Frost MC, Blosnich JR, Lehavot K, et al. Disparities in Documented Drug Use Disorders Between Transgender and Cisgender U.S. Veterans Health Administration Patients. Journal of Addiction Medicine 2020; 25: 25.
75. Fuxman S, Valenti M, Kessel Schneider S, O'Brien KHM, O'Donnell L. Substance use among transgender and cisgender high school students. Journal of LGBT Youth 2021; 18(1): 40-59.
76. Gabrick KS, Chouiari F, Park KE, et al. A comparison of perioperative safety for breast augmentation in cis- vs. trans patients. Annals of Translational Medicine 2021; 9(7).
77. Gamarel KE, Watson RJ, Mouzoon R, Wheldon CW, Fish JN, Fleischer NL. Family Rejection and Cigarette Smoking Among Sexual and Gender Minority Adolescents in the USA. International Journal of Behavioral Medicine 2020; 27(2): 179-87.
78. Garg PR, Uppal L, Mehra S, Mehra D. Mobile Health App for Self-Learning on HIV Prevention Knowledge and Services Among a Young Indonesian Key Population: Cohort Study. JMIR MHealth and UHealth 2020; 8(9): e17646.
79. Gava G, Fisher AD, Alvisi S, et al. Mental health and endocrine telemedicine consultations in transgender subjects during the COVID-19 outbreak in Italy: A cross-sectional web-based survey. Journal of Sexual Medicine 2021; 18(5): 900-7.
80. Gilbert PA, Lee AA, Pass L, et al. Queer in the Heartland: Cancer Risks, Screenings, and Diagnoses among Sexual and Gender Minorities in Iowa. Journal of Homosexuality 2020: 1-17.
81. Goldstein Z, Martinson T, Ramachandran S, Lindner R, Safer JD. Improved Rates of Cervical Cancer Screening Among Transmasculine Patients Through Self-Collected Swabs for High-Risk Human Papillomavirus DNA Testing. Transgender Health 2020; 5(1): 10-7.
82. Gonzales G, Loret de Mola E, Gavulic KA, McKay T, Purcell C. Mental Health Needs Among Lesbian, Gay, Bisexual, and Transgender College Students During the COVID-19 Pandemic. Journal of Adolescent Health 2020; 67(5): 645-8.
83. Gorrell S, Le Grange D, Blalock DV, et al. Gender identity, race/ethnicity and eating pathology in a treatment-seeking community sample. Journal of Behavioral and Cognitive Therapy 2021; 31(1): 77-89.
84. Gosiker BJ, Lesko CR, Rich AJ, et al. Cardiovascular disease risk among transgender women living with HIV in the United States. PLoS ONE [Electronic Resource] 2020; 15(7): e0236177.
85. Grammer AC, Vazquez MM, Fitzsimmons-Craft EE, et al. Characterizing eating disorder diagnosis and related outcomes by sexual orientation and gender identity in a national sample of college students. Eating Behaviors 2021; 42: 101528.
86. Guzman-Gonzalez M, Barrientos J, Saiz JL, et al. [Mental health in a sample of transgender people]. Revista Medica de Chile 2020; 148(8): 1113-20.
87. Halli SS, Isac S, Bhattacharjee P, et al. Suicidality among gender minorities in Karnataka, South India. BMC Psychiatry 2021; 21(1): 25.
88. Harper GW, Crawford J, Lewis K, et al. Mental Health Challenges and Needs among Sexual and Gender Minority People in Western Kenya. International Journal of Environmental Research & Public Health [Electronic Resource] 2021; 18(3): 01.
89. Hawkins M, Deutsch MB, Obedin-Maliver J, et al. Endometrial findings among transgender and gender nonbinary people using testosterone at the time of gender-affirming hysterectomy. Fertility & Sterility 2021; 12: 12.
90. Heino E, Frojd S, Marttunen M, Kaltiala R. Transgender identity is associated with severe suicidal ideation among Finnish adolescents. International Journal of Adolescent Medicine & Health 2021; 15: 15.
91. Heller AT, Berg SS, Prichard JR. University housing reinforces the negative relationship between interpersonal violence, psychological distress, and suicidality in undergraduates, particularly among gender diverse students. Journal of American College Health 2021: 1-9.
92. Henry RS, Perrin PB, Coston BM, Calton JM. Intimate Partner Violence and Mental Health Among Transgender/Gender Nonconforming Adults. Journal of Interpersonal Violence 2021; 36(7-8): 3374-99.
93. Hernandez CJ, Trujillo D, Sicro S, et al. High hepatitis C virus seropositivity, viremia, and associated risk factors among trans women living in San Francisco, California. PLoS ONE [Electronic Resource] 2021; 16(3): e0249219.
94. Hershner S, Jansen EC, Gavidia R, Matlen L, Hoban M, Dunietz GL. Associations Between Transgender Identity, Sleep, Mental Health and Suicidality Among a North American Cohort of College Students. Nature & Science of Sleep 2021; 13: 383-98.
95. Hibbert MP, Wolton A, Weeks H, et al. Psychosocial and sexual factors associated with recent sexual health clinic attendance and HIV testing among trans people in the UK. BMJ Sexual & Reproductive Health 2020; 46(2): 116-25.
96. Hickson F, Appenroth M, Koppe U, Schmidt AJ, Reid D, Weatherburn P. Sexual and Mental Health Inequalities across Gender Identity and Sex-Assigned-at-Birth among Men-Who-Have-Sex-with-Men in Europe: Findings from EMIS-2017. International Journal of Environmental Research & Public Health [Electronic Resource] 2020; 17(20): 10.
97. Hillman J. Lifetime prevalence of intimate partner violence and health-related outcomes among transgender adults age 50. The Gerontologist 2021.
98. Hisle-Gorman E, Schvey NA, Adirim TA, et al. Mental Healthcare Utilization of Transgender Youth Before and After Affirming Treatment. The journal of sexual medicine 2021; 18(8): 1444-54.
99. Holloway IW, Green D, Pickering C, et al. Mental Health and Health Risk Behaviors of Active Duty Sexual Minority and Transgender Service Members in the United States Military. Lgbt Health 2021; 8(2): 152-61.
100. Horwitz AG, Berona J, Busby DR, et al. Variation in Suicide Risk among Subgroups of Sexual and Gender Minority College Students. Suicide & Life-Threatening Behavior 2020; 50(5): 1041-53.
101. Hotton AL, Balthazar C, Jadwin-Cakmak L, et al. Socio-structural Factors Associated with Mental Health, Substance Use, and HIV Risk Among Black Sexual and Gender Minorities in the House and Ball Community. AIDS & Behavior 2020; 24(8): 2319-26.
102. Hughto JMW, Gunn HA, Rood BA, Pantalone DW. Social and Medical Gender Affirmation Experiences Are Inversely Associated with Mental Health Problems in a U.S. Non-Probability Sample of Transgender Adults. Archives of Sexual Behavior 2020; 49(7): 2635-47.
103. Hughto JMW, Meyers DJ, Mimiaga MJ, Reisner SL, Cahill S. Uncertainty and Confusion Regarding Transgender Non-discrimination Policies: Implications for the Mental Health of Transgender Americans. Sexuality Research and Social Policy 2021.
104. Hughto JMW, Pletta D, Gordon L, Cahill S, Mimiaga MJ, Reisner SL. Negative Transgender-Related Media Messages Are Associated with Adverse Mental Health Outcomes in a Multistate Study of Transgender Adults. Lgbt Health 2021; 8(1): 32-41.
105. Hughto JMW, Quinn EK, Dunbar MS, Rose AJ, Shireman TI, Jasuja GK. Prevalence and Co-occurrence of Alcohol, Nicotine, and Other Substance Use Disorder Diagnoses Among US Transgender and Cisgender Adults. JAMA Network Open 2021; 4(2): e2036512.
106. Hung P, Osias E, Konda KA, et al. High Lifetime Prevalence of Syphilis in Men Who Have Sex With Men and Transgender Women Versus Low Lifetime Prevalence in Female Sex Workers in Lima, Peru. Sexually Transmitted Diseases 2020; 47(8): 549-55.
107. Hunt C, Gibson GC, Vander Horst A, et al. Gender diverse college students exhibit higher psychological distress than male and female peers during the novel coronavirus (COVID-19) pandemic. Psychology of Sexual Orientation and Gender Diversity 2021; 8(2): 238-44.
108. Imhof RL, Davidge-Pitts CJ, Miest RYN, Nippoldt TB, Tollefson MM. Dermatologic disorders in transgender patients: A retrospective cohort of 442 patients. Journal of the American Academy of Dermatology 2020; 83(5): 1516-8.
109. Indrawati F, Sudaryo MK. Consistency of condom use associated with HIV among transgender in indonesia: Secondary data analysis of IBBS 2015. Indian Journal of Public Health Research and Development 2020; 11(3): 1984-9.
110. Jacoby A, Rifkin W, Zhao LC, Bluebond-Langner R. Incidence of Cancer and Premalignant Lesions in Surgical Specimens of Transgender Patients. Plastic & Reconstructive Surgery 2021; 147(1): 194-8.
111. James HA, Chang AY, Imhof RL, et al. A community-based study of demographics, medical and psychiatric conditions, and gender dysphoria/incongruence treatment in transgender/gender diverse individuals. Biology of sex differences 2020; 11(1): 55.
112. Jarrett BA, Peitzmeier SM, Restar A, et al. Gender-affirming care, mental health, and economic stability in the time of COVID-19: A multi-national, cross-sectional study of transgender and nonbinary people. PLoS ONE 2021; 16(7): e0254215.
113. Jin H, Restar A, Goedel WC, et al. Maternal Support Is Protective Against Suicidal Ideation Among a Diverse Cohort of Young Transgender Women. Lgbt Health 2020; 7(7): 349-57.
114. Jones BA, Bowe M, McNamara N, Guerin E, Carter T. Exploring the mental health experiences of young trans and gender diverse people during the Covid-19 pandemic. International Journal of Transgender Health 2021.
115. Joshi N, Vinu V, Jois SN, Begum F, Salagame KK. The Need, Feasibility and Willingness to Explore "Meditation on Twin Hearts" as a Self-administered Tool for Mental Health Management among Transgender Women: An Exploratory Survey. Community Mental Health Journal 2021.
116. Kaltiala R, Heino E, Tyolajarvi M, Suomalainen L. Adolescent development and psychosocial functioning after starting cross-sex hormones for gender dysphoria. Nordic Journal of Psychiatry 2020; 74(3): 213-9.
117. Kattari SK, Bakko M, Hecht HK, Kattari L. Correlations between healthcare provider interactions and mental health among transgender and nonbinary adults. SSM - Population Health 2020; 10: 100525.
118. Kattari SK, Kattari L, Johnson I, Lacombe-Duncan A, Misiolek BA. Differential Experiences of Mental Health among Trans/Gender Diverse Adults in Michigan. International Journal of Environmental Research & Public Health [Electronic Resource] 2020; 17(18): 18.
119. Katz-Wise SL, Sarda V, Austin SB, Harris SK. Longitudinal effects of gender minority stressors on substance use and related risk and protective factors among gender minority adolescents. PLoS ONE 2021; 16(6): e0250500.
120. Kcomt L, Evans-Polce RJ, Boyd CJ, McCabe SE. Association of transphobic discrimination and alcohol misuse among transgender adults: Results from the U.S. Transgender Survey. Drug & Alcohol Dependence 2020; 215: 108223.
121. Kcomt L, Evans-Polce RJ, Veliz PT, Boyd CJ, McCabe SE. Use of cigarettes and e-cigarettes/vaping among transgender people: Results from the 2015 US Transgender Survey. American Journal of Preventive Medicine 2020; 59(4): 538-47.
122. Khorashad BS, Talaei A, Aghili Z, Arabi A. Psychiatric morbidity adult transgender people in Iran. Journal of Psychiatric Research 2021; 142: 33-9.
123. Kidd JD, Goetz TG, Shea EA, Bockting WO. Prevalence and minority-stress correlates of past 12-month prescription drug misuse in a national sample of transgender and gender nonbinary adults: Results from the U.S. Transgender Survey. Drug & Alcohol Dependence 2021; 219: 108474.
124. Kidd JD, Jackman KB, Barucco R, et al. Understanding the Impact of the COVID-19 Pandemic on the Mental Health of Transgender and Gender Nonbinary Individuals Engaged in a Longitudinal Cohort Study. Journal of Homosexuality 2021; 68(4): 592-611.
125. Kirby SR, Linde JA. Understanding the Nutritional Needs of Transgender and Gender-Nonconforming Students at a Large Public Midwestern University. Transgender Health 2020; 5(1): 33-41.
126. Kittiteerasack P, Matthews AK, Steffen A, et al. The influence of minority stress on indicators of suicidality among lesbian, gay, bisexual and transgender adults in Thailand. Journal of Psychiatric & Mental Health Nursing 2020; 15: 15.
127. Klaver M, de Mutsert R, van der Loos M, et al. Hormonal Treatment and Cardiovascular Risk Profile in Transgender Adolescents. Pediatrics 2020; 145(3): 03.
128. Klemmer CL, Arayasirikul S, Raymond HF. Transphobia-Based Violence, Depression, and Anxiety in Transgender Women: The Role of Body Satisfaction. Journal of Interpersonal Violence 2021; 36(5-6): 2633-55.
129. Kneale D, Becares L. Discrimination as a predictor of poor mental health among LGBTQ+ people during the COVID-19 pandemic: cross-sectional analysis of the online Queerantine study. BMJ Open 2021; 11(6): e049405.
130. Komarudin U, Wahyono TYM. Risk factors associated with HIV infection among male to transvestites in five cities in Indonesia in 2015. Indian Journal of Public Health Research and Development 2020; 11(1): 1606-11.
131. Konrad M, Kostev K. Increased prevalence of depression, anxiety, and adjustment and somatoform disorders in transsexual individuals. Journal of Affective Disorders 2020; 274: 482-5.
132. Kota KK, Salazar LF, Culbreth RE, Crosby RA, Jones J. Psychosocial mediators of perceived stigma and suicidal ideation among transgender women. BMC Public Health 2020; 20(1): 125.
133. Kuper LE, Stewart S, Preston S, Lau M, Lopez X. Body Dissatisfaction and Mental Health Outcomes of Youth on Gender-Affirming Hormone Therapy. Pediatrics 2020; 145(4): 04.
134. Lacombe-Duncan A, Warren L, Kay ES, et al. Mental health among transgender women living with HIV in Canada: findings from a national community-based research study. AIDS Care 2021; 33(2): 192-200.
135. Lane M, Kirsch MJ, Sluiter EC, et al. Prevalence of Psychosocial Distress in Transmen Seeking Gender-Affirming Mastectomy. Plastic & Reconstructive Surgery 2020; 146(6): 1376-80.
136. Lee H, Operario D, van den Berg JJ, Yi H, Choo S, Kim SS. Health Disparities Among Transgender Adults in South Korea. Asia-Pacific Journal of Public Health 2020; 32(2-3): 103-10.
137. Lee H, Tomita KK, Habarth JM, et al. Internalized transphobia and mental health among transgender adults: A nationwide cross-sectional survey in South Korea. International Journal of Transgender Health 2020; 21(2): 182-93.
138. Lee H, Yi H, Rider GN, et al. Transgender Adults' Public Bathroom-Related Stressors and Their Association with Depressive Symptoms: A Nationwide Cross-Sectional Study in South Korea. LGBT Health 2021.
139. Lehmann K, Rosato M, McKenna H, Leavey G. Autism trait prevalence in treatment seeking adolescents and adults attending specialist gender services. European Psychiatry: the Journal of the Association of European Psychiatrists 2020; 63(1): e23.
140. Leon K, O'Bryan J, Wolf-Gould C, Turell SC, Gadomski A. Prevalence and Risk Factors for Nonsuicidal Self-Injury in Transgender and Gender-Expansive Youth at a Rural Gender Wellness Clinic. Transgender Health 2021; 6(1): 43-50.
141. Lett E, Dowshen NL, Baker KE. Intersectionality and Health Inequities for Gender Minority Blacks in the U.S. American Journal of Preventive Medicine 2020; 59(5): 639-47.
142. Levit D, Yaish I, Shtrozberg S, Aloush V, Greenman Y, Ablin JN. Pain and transition: evaluating fibromyalgia in transgender individuals. Clinical & Experimental Rheumatology 2021; 13: 13.
143. Lim G, Waling A, Lyons A, Pepping CA, Brooks A, Bourne A. Trans and Gender‐Diverse peoples' experiences of crisis helpline services. Health & Social Care in the Community 2021; 29(3): 672-84.
144. Lindley L, Bauerband L, Galupo MP. Using a Comprehensive Proximal Stress Model to Predict Alcohol Use. Transgender Health 2021; 6(3): 164-74.
145. Linsenmeyer WR, Katz IM, Reed JL, Giedinghagen AM, Lewis CB, Garwood SK. Disordered Eating, Food Insecurity, and Weight Status Among Transgender and Gender Nonbinary Youth and Young Adults: A Cross-Sectional Study Using a Nutrition Screening Protocol. LGBT Health 2021; 8(5): 359-66.
146. Liu Y, Xin Y, Qi J, et al. The Desire and Status of Gender-Affirming Hormone Therapy and Surgery in Transgender Men and Women in China: A National Population Study. Journal of Sexual Medicine 2020; 17(11): 2291-8.
147. Logie CH, Lacombe-Duncan A, Wang Y, et al. Adapting the psychological mediation framework for cisgender and transgender sexual minorities in Jamaica: Implications from latent versus observed variable approaches to sexual stigma. Social Science & Medicine 2020; 245: 112663.
148. Logie CH, Wang Y, Marcus N, Lalor P, Williams D, Levermore K. Pathways from Police, Intimate Partner, and Client Violence to Condom Use Outcomes among Sex Workers in Jamaica. International Journal of Behavioral Medicine 2020; 27(4): 378-88.
149. Long JE, Ulrich A, White E, et al. Characterizing Men Who Have Sex with Transgender Women in Lima, Peru: Sexual Behavior and Partnership Profiles. AIDS & Behavior 2020; 24(3): 914-24.
150. LoSchiavo C, Acuna N, Halkitis PN. Evidence for the Confluence of Cigarette Smoking, Other Substance Use, and Psychosocial and Mental Health in a Sample of Urban Sexual Minority Young Adults: The P18 Cohort Study. Annals of Behavioral Medicine 2021; 55(4): 308-20.
151. LoSchiavo C, Greene RE, Halkitis PN. Human Papillomavirus Prevalence, Genotype Diversity, and Risk Factors Among Transgender Women and Nonbinary Participants in the P18 Cohort Study. AIDS Patient Care & STDs 2020; 34(12): 502-5.
152. Lozano-Verduzco I, Melendez R. Transgender individuals in Mexico: exploring characteristics and experiences of discrimination and violence. Psychology & Sexuality 2021; 12(3): 235-47.
153. Luz PM, Jalil EM, Castilho J, et al. Association of Discrimination, Violence, and Resilience with Depressive Symptoms Among Transgender Women in Rio de Janeiro, Brazil: A Cross-Sectional Analysis. Transgender Health 2021.
154. Lyons CE, Olawore O, Turpin G, et al. Intersectional stigmas and HIV-related outcomes among a cohort of key populations enrolled in stigma mitigation interventions in Senegal. AIDS 2020; 34 Suppl 1: S63-S71.
155. Madhavan M, Reddy MM, Chinnakali P, Kar SS, Lakshminarayanan S. High levels of non-communicable diseases risk factors among transgenders in Puducherry, South India. Journal of Family Medicine & Primary Care 2020; 9(3): 1538-43.
156. Majumder A, Chatterjee S, Chaudhuri S, Chakraborty S. Lives of Gender Incongruent Community: An Indian Subset Chants 'All is Well'. Indian Journal of Endocrinology and Metabolism 2020; 24(6): 525-31.
157. Mak J, Shires DA, Zhang Q, et al. Suicide Attempts Among a Cohort of Transgender and Gender Diverse People. American Journal of Preventive Medicine 2020; 59(4): 570-7.
158. Maksut JL, Sanchez TH, Wiginton JM, et al. Gender identity and sexual behavior stigmas, severe psychological distress, and suicidality in an online sample of transgender women in the United States. Annals of Epidemiology 2020; 52: 15-22.
159. Martino RJ, Krause KD, Griffin M, et al. A Nationwide Survey of COVID-19 Testing in LGBTQ+ Populations in the United States. Public Health Reports 2021; 136(4): 493-507.
160. Martinson TG, Ramachandran S, Lindner R, Reisman T, Safer JD. High Body Mass Index Is a Significant Barrier to Gender-Confirmation Surgery for Transgender and Gender-Nonbinary Individuals. Endocrine Practice 2020; 26(1): 6-15.
161. McDowell A, Raifman J, Progovac AM, Rose S. Association of Nondiscrimination Policies With Mental Health Among Gender Minority Individuals. JAMA Psychiatry 2020; 77(9): 952-8.
162. McFarland W, Wesson P, Turner C, et al. High HIV Incidence Among Young and Racial/Ethnic Minority Transgender Women in San Francisco: Results of a Longitudinal Cohort Study. Journal of Acquired Immune Deficiency Syndromes: JAIDS 2020; 84(1): e7-e10.
163. McInroy LB, Beaujolais B, Leung VWY, Craig SL, Eaton AD, Austin A. Comparing asexual and non-asexual sexual minority adolescents and young adults: stressors, suicidality and mental and behavioural health risk outcomes. Psychology & Sexuality 2020.
164. McNichols CHL, O'Brien-Coon D, Fischer B. Patient-reported satisfaction and quality of life after trans male gender affirming surgery. International Journal of Transgender Health 2020; 21(4): 410-7.
165. Mereish EH, Feinstein BA, Mamey MR, Goldbach JT. Characteristics and Perceptions of Sexual and Gender Minority Youth Who Utilized a National Sexual and Gender Minority-Focused Crisis Service. Crisis: Journal of Crisis Intervention & Suicide 2021: 1-8.
166. Meyer IH, Blosnich JR, Choi SK, Harper GW, Russell ST. Suicidal Behavior and Coming Out Milestones in Three Cohorts of Sexual Minority Adults. LGBT Health 2021; 8(5): 340-8.
167. Miller WM, Miller WC, Barrington C, et al. Sex work, discrimination, drug use and violence: a pattern for HIV risk among transgender sex workers compared to MSM sex workers and other MSM in Guatemala. Global Public Health 2020; 15(2): 262-74.
168. Modrego Pardo I, Gomez Balaguer M, Hurtado Murillo F, Cunat Navarro E, Sola Izquierdo E, Morillas Arino C. Self-injurious and suicidal behaviour in a transsexual adolescent and young adult population, treated at a specialised gender identity unit in Spain. Endocrinologia Diabetes y Nutricion 2020; 16: 16.
169. Mohajer MA, Adibi A, Mozafari AA, Sahebi A, Bakhtiyari A. Suicidal ideation in patients with gender identity disorder in western iran from march 2019 to march 2020. International Journal of Medical Toxicology and Forensic Medicine 2020; 10(4).
170. Mohr S, Gygax LN, Imboden S, Mueller MD, Kuhn A. Screening for HPV and dysplasia in transgender patients: Do we need it? European Journal of Obstetrics, Gynecology, & Reproductive Biology 2021; 260: 177-82.
171. Mueses Marín HF, Alvarado Llano BE, Tello Bolívar IC, Martínez Cajas JL, Galindo Quintero J. Examining a syndemic framework for HIV and Sexually Transmitted Infections Risk in Cali, Colombia. Hacia promoc salud 2020; 25(2): 140-53.
172. Mullins ES, Geer R, Metcalf M, et al. Thrombosis Risk in Transgender Adolescents Receiving Gender-Affirming Hormone Therapy. Pediatrics 2021; 147(4): 1-9.
173. Mullins ES, Geer R, Metcalf M, et al. Thrombosis Risk in Transgender Adolescents Receiving Gender-Affirming Hormone Therapy. Pediatrics 2021; 22: 22.
174. Murphy J, Prentice F, Walsh R, Catmur C, Bird G. Autism and transgender identity: Implications for depression and anxiety. Research in Autism Spectrum Disorders 2020; 69.
175. Mustanski B, Ryan DT, Newcomb ME, D'Aquila RT, Matson M. Very High HIV Incidence and Associated Risk Factors in a Longitudinal Cohort Study of Diverse Adolescent and Young Adult Men Who Have Sex with Men and Transgender Women. AIDS & Behavior 2020; 24(6): 1966-75.
176. Nadoushan AHJ, Bahramian A, Taban M, et al. High-risk sexual behaviors among transgender individuals in tehran, iran. Acta Medica Iranica 2021; 59(2): 113-7.
177. Nagata JM, Murray SB, Compte EJ, et al. Community norms for the Eating Disorder Examination Questionnaire (EDE-Q) among transgender men and women. Eating Behaviors 2020; 37: 101381.
178. Nanthaprut P, Manojai N, Chanlearn P, et al. Comparison of HIV-Positive Incidence Among Transgender Women and Men Who Have Sex with Men at Stand-Alone and Mobile Voluntary Counseling and Testing Facilities in Chiang Mai Province, Thailand. AIDS Patient Care & Stds 2021; 35(4): 116-25.
179. Nematollahi A, Farnam F, Gharibzadeh S, Khoda-Khah P. Discrimination, violence, and suicide in transgender women in Iran. Health care for women international 2021: 1-12.
180. Newcomb ME, Hill R, Buehler K, Ryan DT, Whitton SW, Mustanski B. High Burden of Mental Health Problems, Substance Use, Violence, and Related Psychosocial Factors in Transgender, Non-Binary, and Gender Diverse Youth and Young Adults. Archives of Sexual Behavior 2020; 49(2): 645-59.
181. Niforatos JD, Wanta JW, Durbak E, Cavendish J, Yax JA. Prevalence of Human Immunodeficiency Virus and Opportunistic Infections Among Transgender Patients in the Clinical Setting: An All-Payer Electronic Health Record Database Study. Transgender Health 2020; 5(3): 191-5.
182. Nobili A, Glazebrook C, Bouman WP, Baron-Cohen S, Arcelus J. The stability of autistic traits in transgender adults following cross-sex hormone treatment. International Journal of Transgender Health 2020; 21(4): 431-9.
183. Nolan BJ, Leemaqz SY, Ooi O, et al. Prevalence of polycythaemia with different formulations of testosterone therapy in transmasculine individuals. Internal Medicine Journal 2021; 51(6): 873-8.
184. Nowaskie DZ, Filipowicz AT, Choi Y, Fogel JM. Eating disorder symptomatology in transgender patients: Differences across gender identity and gender affirmation. International Journal of Eating Disorders 2021; 54(8): 1493-9.
185. Papadopulos NA, Lelle JD, Zavlin D, et al. Psychological Pathologies and Sexual Orientation in Transgender Women Undergoing Gender Confirming Treatment. Annals of Plastic Surgery 2020; 84(3): 312-6.
186. Parr NJ. Sexual Assault and Co-Occurrence of Mental Health Outcomes Among Cisgender Female, Cisgender Male, and Gender Minority U.S. College Students. Journal of Adolescent Health 2020; 67(5): 722-6.
187. Passaro RC, Segura ER, Gonzales-Saavedra W, et al. Sexual Partnership-Level Correlates of Intimate Partner Violence Among Men Who Have Sex with Men and Transgender Women in Lima, Peru. Archives of Sexual Behavior 2020; 49(7): 2703-13.
188. Passaro RC, Segura ER, Lama JR, et al. High-Risk, but Hidden: Binge Drinking among Men Who Have Sex with Men and Transgender Women in Lima, Peru, 2012-2014. Substance Use & Misuse 2020; 55(3): 399-404.
189. Paulino-Ramirez R, Hearld KR, Butane SA, et al. Serological Confirmed Syphilis Among Transgender Women in Dominican Republic. Transgender Health 2021.
190. Peitzmeier SM, Silberholz J, Gardner IH, Weinand J, Acevedo K. Time to First Onset of Chest Binding-Related Symptoms in Transgender Youth. Pediatrics 2021; 147(3).
191. Peterson CM, Mara CA, Conard LAE, Grossoehme D. The relationship of the UPPS model of impulsivity on bulimic symptoms and non-suicidal self-injury in transgender youth. Eating Behaviors 2020; 39: 101416.
192. Peterson CM, Toland MD, Matthews A, Mathews S, Thompson F, Conard LAE. Exploring the Eating Disorder Examination Questionnaire in treatment seeking transgender youth. Psychology of Sexual Orientation and Gender Diversity 2020; 7(3): 304-15.
193. Pharr JR, Batra K. Propensity Score Analysis Assessing the Burden of Non-Communicable Diseases among the Transgender Population in the United States Using the Behavioral Risk Factor Surveillance System (2017-2019). Healthcare (Basel, Switzerland) 2021; 9(6).
194. Pines HA, Semple SJ, Magis-Rodriguez C, et al. A comparison of the effectiveness of respondent-driven and venue-based sampling for identifying undiagnosed HIV infection among cisgender men who have sex with men and transgender women in Tijuana, Mexico. Journal of the International AIDS Society 2021; 24(3): e25688.
195. Pitasi MA, Clark HA, Chavez PR, DiNenno EA, Delaney KP. HIV Testing and Linkage to Care Among Transgender Women Who Have Sex with Men: 23 U.S. Cities. AIDS & Behavior 2020; 24(8): 2442-50.
196. Platt LF. The Presenting Concerns of Transgender and Gender Nonconforming Clients at University Counseling Centers. Counseling Psychologist 2020; 48(3): 407-31.
197. Plummer RM, Kelting S, Madan R, O'Neil M, Dennis K, Fan F. Cervical Papanicolaou tests in the female-to-male transgender population: should the adequacy criteria be revised in this population? An Institutional Experience. Journal of the American Society of Cytopathology JASC 2021; 29: 29.
198. Polizopoulos-Wilson N, Kindratt TB, Hansoti E, et al. A Needs Assessment Among Transgender Patients at an LGBTQ Service Organization in Texas. Transgender Health 2021; 6(3): 175-83.
199. Polonijo AN, Gardner A, Clinton T, Brown B. Transgender and Gender Nonconforming Patient Experience in the Inland Empire, California. Transgender Health 2020; 5(4): 267-71.
200. Poteat T, Malik M, van der Merwe LLA, et al. PrEP awareness and engagement among transgender women in South Africa: a cross-sectional, mixed methods study. The Lancet HIV 2020; 7(12): e825-e34.
201. Poteat TC, Celentano DD, Mayer KH, et al. Depression, sexual behavior, and HIV treatment outcomes among transgender women, cisgender women and men who have sex with men living with HIV in Brazil and Thailand: a short report. AIDS Care 2020; 32(3): 310-5.
202. Poteat TC, Divsalar S, Streed CG, Feldman JL, Bockting WO, Meyer IH. Cardiovascular Disease in a Population-Based Sample of Transgender and Cisgender Adults. American Journal of Preventive Medicine 2021.
203. Poteat TC, Reisner SL, Miller M, Wirtz AL, American Cohort To Study HIVAATW. Vulnerability to COVID-19-related Harms Among Transgender Women With and Without HIV Infection in the Eastern and Southern U.S. Journal of Acquired Immune Deficiency Syndromes: JAIDS 2020; 85(4): e67-e9.
204. Poteat VP, O'Brien MD, Rosenbach SB, Finch EK, Calzo JP. Depression, Anxiety, and Interest in Mental Health Resources in School-Based Gender-Sexuality Alliances: Implications for Sexual and Gender Minority Youth Health Promotion. Prevention Science 2021; 22(2): 237-46.
205. Prescott MR, Santos GM, Arayasirikul S, Wilson E. Partnership-level correlates of sexual risk taking within the sexual partnerships of young transwomen in San Francisco, California. Sexually Transmitted Infections 2020; 96(1): 58-61.
206. Price-Feeney M, Green AE, Dorison S. Understanding the Mental Health of Transgender and Nonbinary Youth. Journal of Adolescent Health 2020; 66(6): 684-90.
207. Price-Feeney M, Green AE, Dorison SH. Impact of Bathroom Discrimination on Mental Health Among Transgender and Nonbinary Youth. Journal of Adolescent Health 2021; 68(6): 1142-7.
208. Progovac AM, Mullin BO, Dunham E, et al. Disparities in Suicidality by Gender Identity Among Medicare Beneficiaries. American Journal of Preventive Medicine 2020; 58(6): 789-98.
209. Progovac AM, Tran NM, Mullin BO, et al. Elevated Rates of Violence Victimization and Suicide Attempt Among Transgender and Gender Diverse Patients in an Urban, Safety Net Health System. World Medical and Health Policy 2021; 13(2): 176-98.
210. Pyra M, Casimiro I, Rusie L, et al. An Observational Study of Hypertension and Thromboembolism Among Transgender Patients Using Gender-Affirming Hormone Therapy. Transgender Health 2020; 5(1): 1-9.
211. Rabasco A, Andover M. The Influence of State Policies on the Relationship Between Minority Stressors and Suicide Attempts Among Transgender and Gender-Diverse Adults. Lgbt Health 2020; 7(8): 457-60.
212. Rabasco A, Andover M. Suicidal ideation among transgender and gender diverse adults: A longitudinal study of risk and protective factors. Journal of Affective Disorders 2021; 278: 136-43.
213. Radusky PD, Aristegui I, Mandell LN, et al. Examining Factors Associated with Gender Identity Among Individuals Disengaged from HIV Care in Argentina. International Journal of Behavioral Medicine 2021.
214. Radusky PD, Cardozo N, Duarte M, et al. Mental health, substance use, experiences of violence, and access to health care among transgender and non-binary people during the COVID-19 lockdown in Argentina. International Journal of Transgender Health 2021.
215. Radusky PD, Zalazar V, Cardozo N, et al. Reduction of Gender Identity Stigma and Improvements in Mental Health Among Transgender Women Initiating HIV Treatment in a Trans-Sensitive Clinic in Argentina. Transgender Health 2020; 5(4): 216-24.
216. Rajan S, Kumar P, Sangal B, Kumar A, Ramanathan S, Ammassari S. HIV/AIDS-Related risk behaviors, HIV prevalence, and determinants for HIV prevalence among hijra/transgender people in India: Findings from the 2014-2015 integrated biological and behavioural surveillance. Indian Journal of Public Health 2020; 64(Supplement): S53-S60.
217. Ramadhani HO, Crowell TA, Nowak RG, et al. Association of age with healthcare needs and engagement among Nigerian men who have sex with men and transgender women: cross-sectional and longitudinal analyses from an observational cohort. Journal of the International AIDS Society 2020; 23 Suppl 6: e25599.
218. Real AG, Fontanari AMV, Costa AB, et al. Gender dysphoria: prejudice from childhood to adulthood, but no impact on inflammation. A cross-sectional controlled study. Trends in Psychiatry & Psychotherapy 2021; 43(1): 37-46.
219. Reisner SL, Moore CS, Asquith A, Pardee DJ, Mayer KH. Gender Non-affirmation from Cisgender Male Partners: Development and Validation of a Brief Stigma Scale for HIV Research with Transgender Men Who Have Sex with Men (Trans MSM). AIDS & Behavior 2020; 24(1): 331-43.
220. Restar A, Jin H, Breslow A, et al. Legal gender marker and name change is associated with lower negative emotional response to gender-based mistreatment and improve mental health outcomes among trans populations. SSM - Population Health 2020; 11: 100595.
221. Restar AJ, Jin H, Jarrett B, et al. Characterising the impact of COVID-19 environment on mental health, gender affirming services and socioeconomic loss in a global sample of transgender and non-binary people: a structural equation modelling. BMJ Global Health 2021; 6(3): 03.
222. Restar AJ, Jin H, Ogunbajo A, et al. Differences in HIV risk and healthcare engagement factors in Filipinx transgender women and cisgender men who have sex with men who reported being HIV negative, HIV positive or HIV unknown. Journal of the International AIDS Society 2020; 23(8): e25582.
223. Restar AJ, Jin H, Ogunbajo A, et al. Prevalence and Risk Factors of Nonmedical Prescription Opioid Use Among Transgender Girls and Young Women. JAMA Network Open 2020; 3(3): e201015.
224. Rich AJ, Poteat T, Koehoorn M, et al. Development of a computable phenotype to identify a transgender sample for health research purposes: a feasibility study in a large linked provincial healthcare administrative cohort in British Columbia, Canada. BMJ Open 2021; 11(3): e040928.
225. Robbins SJ, Dauda W, Kokogho A, et al. Oral sex practices among men who have sex with men and transgender women at risk for and living with HIV in Nigeria. PLoS ONE [Electronic Resource] 2020; 15(9): e0238745.
226. Rocha A, Barros C, Generoso IP, Bastos FI, Veras MA. HIV continuum of care among trans women and travestis living in Sao Paulo, Brazil. Revista de Saude Publica 2020; 54: 118.
227. Rodriguez-Hart C, Obeng B, Radix A, Goldstein Z, Torian L. Improving Data on the HIV Epidemic in New York City by Identifying Transgender Persons in Medicaid in 2013-2017. Transgender Health 2021.
228. Rothenberg KA, Gologorsky RC, Hojilla JC, et al. Gender-affirming mastectomy in transmasculine patients: does obesity increase complications or revisions? Annals of plastic surgery 2021; 87(1): 24.
229. Rouhani S, Decker MR, Tomko C, et al. Resilience among Cisgender and Transgender Women in Street-Based Sex Work in Baltimore, Maryland. Womens Health Issues 2021; 31(2): 148-56.
230. Rutherford L, Stark A, Ablona A, et al. Health and well-being of trans and non-binary participants in a community-based survey of gay, bisexual, and queer men, and non-binary and Two-Spirit people across Canada. PLoS ONE [Electronic Resource] 2021; 16(2): e0246525.
231. Sabino TE, Avelino-Silva VI, Cavalcantte C, et al. Adherence to antiretroviral treatment and quality of life among transgender women living with HIV/AIDS in Sao Paulo, Brazil. AIDS Care 2021; 33(1): 31-8.
232. Sandfort TGM, Mbilizi Y, Sanders EJ, Guo X, Cummings V, Hamilton EL. HIV incidence in a multinational cohort of men and transgender women who have sex with men in sub-Saharan Africa: Findings from HPTN 075 (vol 16, e0247195, 2021). PLoS ONE 2021; 16(3).
233. Sanfacon K, Leffers A, Miller C, et al. Cross-Sectional Analysis of Medical Conditions in the U.S. Deaf Transgender Community. Transgender Health 2021; 6(3): 132-8.
234. Santos PMRd, Santos KCd, Magalhães LS, et al. Travestis and transsexual women: who are at higher risk for sexually transmitted infections? Rev bras epidemiol 2021; 24: e210017-e.
235. Sartaj D, Krishnan V, Rao R, Ambekar A, Dhingra N, Sharan P. Mental illnesses and related vulnerabilities in the Hijra community: A cross-sectional study from India. International Journal of Social Psychiatry 2020: 20764020950775.
236. Scheim AI, Perez-Brumer AG, Bauer GR. Gender-concordant identity documents and mental health among transgender adults in the USA: a cross-sectional study. The lancet Public Health 2020; 5(4): e196-e203.
237. Schlissel AC, Carpenter R, Avripas S, et al. Substance Misuse and Condomless Sex among Transgender Youth. Transgender Health 2021.
238. Schvey NA, Klein DA, Pearlman AT, Riggs DS. A Descriptive Study of Transgender Active Duty Service Members in the U.S. Military. Transgender Health 2020; 5(3): 149-57.
239. Schweizer VJ, Mowen TJ. Discrimination and Risky Sexual Behavior, Substance Use, and Suicidality among Transgender Individuals. Deviant Behavior 2020.
240. Secco AA, Akselrod H, Czeresnia J, et al. Sexually transmitted infections in persons living with HIV infection and estimated HIV transmission risk: trends over time from the DC Cohort. Sexually Transmitted Infections 2020; 96(2): 89-95.
241. Seelman KL, Kattari SK, Harvey P, Bakko M. Trans Men's Access to Knowledgeable Providers and Their Experiences in Health Care Settings: Differences by Demographics, Mental Health, and Degree of Being "Out" to Providers. Health & Social Work 2021; 45(4): 229-39.
242. Segev-Becker A, Israeli G, Elkon-Tamir E, et al. Children and Adolescents with Gender Dysphoria in Israel: Increasing Referral and Fertility Preservation Rates. Endocrine Practice 2020; 26(4): 423-8.
243. Sergi FD, Wilson EC. Filler Use among Trans Women: Correlates of Feminizing Subcutaneous Injections and Their Health Consequences. Transgender Health 2021; 6(2): 82-90.
244. Sevelius JM, Neilands TB, Dilworth S, Castro D, Johnson MO. Sheroes: Feasibility and Acceptability of a Community-Driven, Group-Level HIV Intervention Program for Transgender Women. AIDS & Behavior 2020; 24(5): 1551-9.
245. Sevelius JM, Xavier J, Chakravarty D, Keatley J, Shade S, Rebchook G. Correlates of Engagement in HIV Care Among Transgender Women of Color in the United States of America. AIDS & Behavior 2021; 25: 3-12.
246. She J, McCall J, Pudwell J, Kielly M, Waddington A. An assessment of the mental health history of patients in a transgender clinic in Kingston, Ontario. The Canadian Journal of Psychiatry / La Revue canadienne de psychiatrie 2020; 65(4): 281-3.
247. She R, Mo PK, Ma T, Liu Y, Lau JT. Impact of Minority Stress and Poor Mental Health on Sexual Risk Behaviors among Transgender Women Sex Workers in Shenyang, China. AIDS & Behavior 2021; 12: 12.
248. She R, Mo PKH, Cai Y, Ma T, Liu Y, Lau JTF. Mental health service utilisation among transgender women sex workers who are at risk of mental health problems in Shenyang, China: An application of minority stress theory. Health & Social Care in the Community 2021.
249. Shrestha R, Galka JM, Azwa I, et al. Willingness to Use HIV Self-Testing and Associated Factors Among Transgender Women in Malaysia. Transgender Health 2020; 5(3): 182-90.
250. Silva DC, Salati LR, Villas-Bôas AP, et al. Factors Associated With Ruminative Thinking in Individuals With Gender Dysphoria. Frontiers in Psychiatry 2021; 12.
251. Silveri MM, Schuttenberg EM, Schmandt K, et al. Clinical Outcomes Following Acute Residential Psychiatric Treatment in Transgender and Gender Diverse Adolescents. JAMA Network Open 2021; 4(6): e2113637-e.
252. Simone M, Askew A, Lust K, Eisenberg ME, Pisetsky EM. Disparities in self-reported eating disorders and academic impairment in sexual and gender minority college students relative to their heterosexual and cisgender peers. International Journal of Eating Disorders 2020; 53(4): 513-24.
253. Singer S, Tkachenko E, Hartman RI, Mostaghimi A. Gender Identity and Lifetime Prevalence of Skin Cancer in the United States. JAMA Dermatology 2020; 156(4): 458-60.
254. Smeaton LM, Kileel EM, Grinsztejn B, et al. Characteristics of REPRIEVE Trial Participants Identifying Across the Transgender Spectrum. Journal of Infectious Diseases 2020; 222(Suppl 1): S31-S40.
255. Snooks MP, McLaren S. Dispositional optimism and suicide among trans and gender diverse adults. Death Studies 2021: 1-9.
256. Snooks MP, McLaren S. Resilience among trans and gender-diverse adults: The protective role of dispositional hope in the perceived burdensomeness-suicide relationship. Psychology of Sexual Orientation and Gender Diversity 2021; 8(1): 57-67.
257. Sok S, Hong R, Chhoun P, et al. HIV risks and recent HIV testing among transgender women in Cambodia: Findings from a national survey. PLoS ONE [Electronic Resource] 2020; 15(9): e0238314.
258. Sorbara JC, Chiniara LN, Thompson S, Palmert MR. Mental Health and Timing of Gender-Affirming Care. Pediatrics 2020; 146(4): 10.
259. Spanos C, Grace JA, Leemaqz SY, et al. The Informed Consent Model of Care for Accessing Gender-Affirming Hormone Therapy Is Associated With High Patient Satisfaction. Journal of Sexual Medicine 2021; 18(1): 201-8.
260. Srivastava A, Davis JP, Patel P, Daniel EE, Karkal S, Rice E. Polyvictimization, Sex Work, and Depressive Symptoms Among Transgender Women and Men Who Have Sex With Men. Journal of Interpersonal Violence 2021: 886260521990840.
261. Srivastava A, Davis JP, Patel P, Daniel EE, Karkal S, Rice E. Sex work, gender transition, family rejection and depressive symptoms among transgender women in India. International Journal of Transgender Health 2021.
262. Srivastava A, Rusow JA, Goldbach JT. Differential Risks for Suicidality and Mental Health Symptoms Among Transgender, Nonbinary, and Cisgender Sexual Minority Youth Accessing Crisis Services. Transgender Health 2021; 6(1): 51-6.
263. Stanton AM, Batchelder AW, Kirakosian N, et al. Differences in mental health symptom severity and care engagement among transgender and gender diverse individuals: Findings from a large community health center. PLoS ONE [Electronic Resource] 2021; 16(1): e0245872.
264. Steele S, Taylor V, Vannoni M, et al. Self-reported access to health care, communicable diseases, violence and perception of legal status among online transgender identifying sex workers in the UK. Public Health 2020; 186: 12-6.
265. Stogner J, Miller BL. Exploring the Relationships between Sexual Orientation and Gender Identity and Youth Synthetic Cannabinoid Use. Substance Use & Misuse 2021; 56(2): 327-32.
266. Storm M, Deuba K, Damas J, et al. Prevalence of HIV, syphilis, and assessment of the social and structural determinants of sexual risk behaviour and health service utilisation among MSM and transgender women in Terai highway districts of Nepal: findings based on an integrated biological and behavioural surveillance survey using respondent driven sampling. BMC Infectious Diseases 2020; 20(1): 402.
267. Stowell JT, Parikh Y, Tilson K, Narayan AK. Lung Cancer Screening Eligibility and Utilization Among Transgender Patients: An Analysis of the 2017-2018 United States Behavioral Risk Factor Surveillance System Survey. Nicotine & Tobacco Research 2020; 22(12): 2164-9.
268. Strang JF, Anthony LG, Song A, et al. In Addition to Stigma: Cognitive and Autism-Related Predictors of Mental Health in Transgender Adolescents. Journal of clinical child and adolescent psychology : the official journal for the Society of Clinical Child and Adolescent Psychology, American Psychological Association, Division 53 2021: 1-18.
269. Strang JF, Chen D, Nelson E, et al. Transgender Youth Executive Functioning: Relationships with Anxiety Symptoms, Autism Spectrum Disorder, and Gender-Affirming Medical Treatment Status. Child psychiatry and human development 2021.
270. Strauss P, Cook A, Watson V, et al. Mental health difficulties among trans and gender diverse young people with an autism spectrum disorder (ASD): Findings from Trans Pathways. Journal of Psychiatric Research 2021; 137: 360-7.
271. Strauss P, Cook A, Winter S, Watson V, Wright Toussaint D, Lin A. Mental Health Issues and Complex Experiences of Abuse Among Trans and Gender Diverse Young People: Findings from Trans Pathways. Lgbt Health 2020; 7(3): 128-36.
272. Strauss P, Cook A, Winter S, Watson V, Wright Toussaint D, Lin A. Associations between negative life experiences and the mental health of trans and gender diverse young people in Australia: findings from Trans Pathways. Psychological Medicine 2020; 50(5): 808-17.
273. Suarez NA, Peitzmeier SM, Potter J, Samandur A, Reisner SL. Preliminary findings for adverse childhood experiences and associations with negative physical and mental health and victimization in transmasculine adults. Child abuse & neglect 2021; 118: 105161.
274. Swartz JA, Ducheny K, Holloway T, Stokes L, Willis S, Kuhns LM. A Latent Class Analysis of Chronic Health Conditions Among HIV-Positive Transgender Women of Color. AIDS & Behavior 2021; 25: 52-63.
275. Tan KKH, Ellis SJ, Schmidt JM, Byrne JL, Veale JF. Mental Health Inequities among Transgender People in Aotearoa New Zealand: Findings from the Counting Ourselves Survey. International Journal of Environmental Research & Public Health [Electronic Resource] 2020; 17(8): 21.
276. Tan KKH, Treharne GJ, Ellis SJ, Schmidt JM, Veale JF. Enacted stigma experiences and protective factors are strongly associated with mental health outcomes of transgender people in Aotearoa/New Zealand. International Journal of Transgender Health 2021; 22(3): 269-80.
277. Tan KKH, Yee A, Veale JF. "Being Trans Intersects with My Cultural Identity": Social Determinants of Mental Health among Asian Transgender People. Transgender Health 2021.
278. Tantirattanakulchai P, Hounnaklang N. Associations between clusters of perceived social support level, depression, and suicidal ideation among transgender women: a latent class analysis. Journal of Public Health Research 2021.
279. Taylor J, Power J, Smith E, Rathbone M. Bisexual mental health and gender diversity: Findings from the 'Who I Am' study. Australian Journal Of General Practice 2020; 49(7): 392-9.
280. Tebbe EA, Simone M, Wilson E, Hunsicker M. A dangerous visibility: Moderating effects of antitrans legislative efforts on trans and gender-diverse mental health. Psychology of Sexual Orientation and Gender Diversity 2021.
281. Teixeira SL, Jalil CM, Jalil EM, et al. Evidence of an untamed HIV epidemic among MSM and TGW in Rio de Janeiro, Brazil: a 2018 to 2020 cross-sectional study using recent infection testing. Journal of the International Aids Society 2021; 24(6): e25743.
282. To M, Zhang Q, Bradlyn A, et al. Visual Conformity With Affirmed Gender or "Passing": Its Distribution and Association With Depression and Anxiety in a Cohort of Transgender People. Journal of Sexual Medicine 2020; 17(10): 2084-92.
283. Torres TS, Hoagland B, Bezerra DRB, et al. Impact of COVID-19 Pandemic on Sexual Minority Populations in Brazil: An Analysis of Social/Racial Disparities in Maintaining Social Distancing and a Description of Sexual Behavior. AIDS & Behavior 2021; 25(1): 73-84.
284. Treharne GJ, Riggs DW, Ellis SJ, Flett JAM, Bartholomaeus C. Suicidality, self-harm, and their correlates among transgender and cisgender people living in Aotearoa/New Zealand or Australia. International Journal of Transgender Health 2020; 21(4): 440-54.
285. Turban JL. Pubertal Suppression for Transgender Youth and Risk of Suicidal Ideation (vol 145, e20191725, 2020). Pediatrics 2021; 147(4).
286. Turban JL, King D, Li JJ, Keuroghlian AS. Timing of Social Transition for Transgender and Gender Diverse Youth, K-12 Harassment, and Adult Mental Health Outcomes. The Journal of adolescent health : official publication of the Society for Adolescent Medicine 2021.
287. Turner BJ, Robillard CL, Ames ME, Craig SG. Prevalence and Correlates of Suicidal Ideation and Deliberate Self-harm in Canadian Adolescents During the Coronavirus Disease 2019 Pandemic. Canadian journal of psychiatry Revue canadienne de psychiatrie 2021: 7067437211036612.
288. Twahirwa Rwema JO, Lyons CE, Herbst S, et al. HIV infection and engagement in HIV care cascade among men who have sex with men and transgender women in Kigali, Rwanda: a cross-sectional study. Journal of the International AIDS Society 2020; 23 Suppl 6: e25604.
289. Uaamnuichai S, Panyakhamlerd K, Suwan A, et al. Neovaginal and anal high-risk human papillomavirus DNA among Thai transgender women in gender-health clinics. Sexually Transmitted Diseases 2021; 01: 01.
290. Valente PK, Schrimshaw EW, Dolezal C, LeBlanc AJ, Singh AA, Bockting WO. Stigmatization, Resilience, and Mental Health Among a Diverse Community Sample of Transgender and Gender Nonbinary Individuals in the U.S. Archives of Sexual Behavior 2020; 49(7): 2649-60.
291. Van Cauwenberg G, Dhondt K, Motmans J. Ten years of experience in counseling gender diverse youth in Flanders, Belgium. A clinical overview. International journal of impotence research 2021.
292. van Heesewijk JO, Dreijerink KMA, Wiepjes CM, et al. Long-Term Gender-Affirming Hormone Therapy and Cognitive Functioning in Older Transgender Women Compared With Cisgender Women and Men. The journal of sexual medicine 2021; 18(8): 1434-43.
293. Vance SR, Jr., Boyer CB, Glidden DV, Sevelius J. Mental Health and Psychosocial Risk and Protective Factors Among Black and Latinx Transgender Youth Compared With Peers. JAMA Network Open 2021; 4(3): e213256.
294. Veronese V, Traeger M, Oo ZM, et al. HIV incidence and factors associated with testing positive for HIV among men who have sex with men and transgender women in Myanmar: data from community-based HIV testing services. Journal of the International AIDS Society 2020; 23(2): e25454.
295. Vi VTT, Long KQ, Hong L, et al. HIV Prevalence and Factors Related to HIV Infection Among Transgender Women in Vietnam: A Respondent Driven Sampling Approach. AIDS & Behavior 2020; 24(11): 3132-41.
296. Walker N, Parag V, Wong SF, et al. Use of e-cigarettes and smoked tobacco in youth aged 14-15 years in New Zealand: findings from repeated cross-sectional studies (2014-19). The lancet Public Health 2020; 5(4): e204-e12.
297. Wang H, Zeng C, Li X, et al. Violence Victimization and Condomless Anal Intercourse Among Transgender Women in China: Mediating Role of Depression. AIDS & Behavior 2021; 25(5): 1597-605.
298. Wang KH, McAvay G, Warren A, et al. Examining Health Care Mobility of Transgender Veterans Across the Veterans Health Administration. Lgbt Health 2021; 8(2): 143-51.
299. Wang Q, Chang R, Wang Y, et al. Correlates of alcohol and illicit drug use before commercial sex among transgender women with a history of sex work in China. Sexual Health 2020; 17(1): 45-52.
300. Wang Y, Yu H, Yang Y, et al. Mental Health Status of Cisgender and Gender-Diverse Secondary School Students in China. JAMA Network Open 2020; 3(10): e2022796.
301. Wansom T, Muangnoicharoen S, Nitayaphan S, et al. Risk Factors for HIV sero-conversion in a high incidence cohort of men who have sex with men and transgender women in Bangkok, Thailand. EClinicalMedicine 2021.
302. Waterschoot M, Hoebeke P, Verla W, et al. Urethral complications after metoidioplasty for genital gender affirming surgery. Journal of Sexual Medicine 2021; 18(7): 1271-9.
303. Wathelet M, Duhem S, Vaiva G, et al. Factors Associated With Mental Health Disorders Among University Students in France Confined During the COVID-19 Pandemic. JAMA Network Open 2020; 3(10): e2025591.
304. Wathelet M, Fovet T, Jousset A, et al. Prevalence of and factors associated with post-traumatic stress disorder among French university students 1 month after the COVID-19 lockdown. Translational psychiatry 2021; 11(1): 327.
305. Watson RJ, Fish JN, McKay T, Allen SH, Eaton L, Puhl RM. Substance Use Among a National Sample of Sexual and Gender Minority Adolescents: Intersections of Sex Assigned at Birth and Gender Identity. Lgbt Health 2020; 7(1): 37-46.
306. Wesson P, Vittinghoff E, Turner C, Arayasirikul S, McFarland W, Wilson E. Intercategorical and Intracategorical Experiences of Discrimination and HIV Prevalence Among Transgender Women in San Francisco, CA: A Quantitative Intersectionality Analysis. American Journal of Public Health 2021; 111(3): 446-56.
307. Wichaidit W, Assanangkornchai S, Chongsuvivatwong V. Disparities in behavioral health and experience of violence between cisgender and transgender Thai adolescents. PLoS ONE 2021; 16(5): e0252520.
308. Wiepjes CM, den Heijer M, Bremmer MA, et al. Trends in suicide death risk in transgender people: results from the Amsterdam Cohort of Gender Dysphoria study (1972-2017). Acta Psychiatrica Scandinavica 2020; 141(6): 486-91.
309. Wilkerson JM, Di Paola A, McCurdy S, Schick V. Covariates of hazardous alcohol use among sexual and gender minorities in Texas: Identifying the most vulnerable. Addictive Behaviors 2020; 105: 106327.
310. Williams EC, Chen JA, Frost MC, et al. Receipt of evidence-based alcohol-related care in a national sample of transgender patients with unhealthy alcohol use: Overall and relative to non-transgender patients. Journal of Substance Abuse Treatment 2021: 108565.
311. Williams EC, Frost MC, Rubinsky AD, et al. Patterns of Alcohol Use Among Transgender Patients Receiving Care at the Veterans Health Administration: Overall and Relative to Nontransgender Patients. Journal of Studies on Alcohol & Drugs 2021; 82(1): 132-41.
312. Wilson EC, Dhakal M, Sharma S, et al. Population-based HIV prevalence, stigma and HIV risk among trans women in Nepal. BMC Infectious Diseases 2021; 21(1): 128.
313. Wilson EC, Hernandez CJ, Scheer S, et al. Improved PrEP Awareness and Use among Trans Women in San Francisco, California. AIDS and behavior 2021.
314. Wilson EC, Jalil EM, Moreira RI, et al. High risk and low HIV prevention behaviours in a new generation of young trans women in Brazil. AIDS Care 2021; 33(8): 997-1001.
315. Witte TK, Kramper S, Carmichael KP, Chaddock M, Gorczyca K. A survey of negative mental health outcomes, workplace and school climate, and identity disclosure for lesbian, gay, bisexual, transgender, queer, questioning, and asexual veterinary professionals and students in the United States and United Kingdom. Journal of the American Veterinary Medical Association 2020; 257(4): 417-31.
316. Wolfe HL, Biello KB, Reisner SL, Mimiaga MJ, Cahill SR, Hughto JMW. Transgender-related discrimination and substance use, substance use disorder diagnosis and treatment history among transgender adults. Drug & Alcohol Dependence 2021; 223: 108711.
317. Wolford-Clevenger C. A daily diary study of intrusive PTSD symptoms and suicidal ideation among transgender and gender diverse adults. Psychological Trauma: Theory, Research, Practice, and Policy 2021.
318. Wolford-Clevenger C, Flores LY, Stuart GL. Proximal correlates of suicidal ideation among transgender and gender diverse people: A preliminary test of the three-step theory. Suicide & life-threatening behavior 2021.
319. Yan L, Yan Z, Wilson E, et al. Awareness and Willingness to use HIV Pre-exposure Prophylaxis (PrEP) Among Trans Women in China: A Community-Based Survey. AIDS & Behavior 2021; 25(3): 866-74.
320. Yi S, Chann N, Chhoun P, Tuot S, Mun P, Brody C. Social marginalization, gender-based violence, and binge drinking among transgender women in Cambodia. Drug & Alcohol Dependence 2020; 207: 107802.
321. Yockey A, King K, Vidourek R. Past-Year Suicidal Ideation Among Transgender Individuals in the United States. Archives of Suicide Research 2020: 1-11.
322. Yousuf T, Naz M, Roberson CB, Wise SM, Rowland DL. Depression as a Function of Social Support in Transgender and Cisgender Individuals with Sexually Transmitted Diseases. International Journal of Environmental Research & Public Health [Electronic Resource] 2021; 18(5): 03.
323. Yunihastuti E, Teeratakulpisarn N, Jeo WS, et al. Incidence, clearance, persistence and factors related with high-risk anal HPV persistence in South-East Asian MSM and transgender women. AIDS 2020; 34(13): 1933-41.
324. Zea MC, Barnett AP, Rio-Gonzalez AMD, et al. Experiences of Violence and Mental Health Outcomes among Colombian Men who have Sex with Men (MSM) and Transgender Women. Journal of Interpersonal Violence 2021: 886260521997445.
325. Zhang J, Wang P, Zhang F, Lo HHM. Using mental health screening instruments for understanding depression and personality profiles among Chinese transgender individuals. China Journal of Social Work 2020; 13(3): 283-98.
326. Zhang Z, Chien H-Y, Wilkins KK, Gorman BK, Reczek R. Parenthood, stress, and well-being among cisgender and transgender gay and lesbian adults. Journal of Marriage and Family 2021.
327. Zwickl S, Angus LM, Qi AWF, et al. The impact of the first three months of the COVID-19 pandemic on the Australian trans community. International Journal of Transgender Health 2021.
328. Zwickl S, Wong AFQ, Dowers E, et al. Factors associated with suicide attempts among Australian transgender adults. BMC Psychiatry 2021; 21(1): 81.
